# Genomic differences between the new *Fusarium oxysporum* f. sp. *apii* (*Foa*) race 4 on celery, the less virulent *Foa* races 2 and 3, and the avirulent on celery f. sp. *coriandrii*

**DOI:** 10.1186/s12864-020-07141-5

**Published:** 2020-10-20

**Authors:** Peter Henry, Sukhwinder Kaur, Quyen Anh Tran Pham, Radwan Barakat, Samuel Brinker, Hannah Haensel, Oleg Daugovish, Lynn Epstein

**Affiliations:** 1 Department of Plant Pathology, University of California, Davis, California, 95616-8680 USA; 2 USDA-ARS, 1636 East Alisal St., Salinas, CA 93905 USA; 3 Current address: Janssen Biopharma, Inc., 260 E Grand Ave., South San Francisco, CA 94080 USA; 4 Department of Plant Production & Protection, College of Agriculture, Hebron University, Hebron, Palestine; 5 University of California Cooperative Extension, 669 County Square Drive, Suite 100, Ventura, CA 93003 USA

**Keywords:** *Apium graveolens*, Celery, Cilantro, Coriander, *Coriandrum sativum*, Differentially expressed genes, *Fusarium oxysporum*, *Fusarium oxysporum* species complex, Fusarium yellows, Transposable elements

## Abstract

**Background:**

Members of the *F. oxysporium* species complex (FOSC) in the f. sp. *apii* (*Foa*) are pathogenic on celery and those in f. sp. *coriandrii* (*Foci*) are pathogenic on coriander (=cilantro)*. Foci* was first reported in California in 2005; a new and highly aggressive race 4 of *Foa* was observed in 2013 in California. Preliminary evidence indicated that *Foa* can also cause disease on coriander, albeit are less virulent than *Foci*. Comparative genomics was used to investigate the evolutionary relationships between *Foa* race 4, *Foa* race 3, and the *Foci*, which are all in FOSC Clade 2, and *Foa* race 2, which is in FOSC Clade 3.

**Results:**

A phylogenetic analysis of 2718 single-copy conserved genes and mitochondrial DNA sequence indicated that *Foa* races 3 and 4 and the *Foci* are monophyletic within FOSC Clade 2; these strains also are in a single somatic compatibility group. However, in the accessory genomes, the *Foci* versus *Foa* races 3 and 4 differ in multiple contigs. Based on significantly increased expression of *Foa* race 4 genes *in planta* vs. in vitro, we identified 23 putative effectors and 13 possible pathogenicity factors. PCR primers for diagnosis of either *Foa* race 2 or 4 and the *Foci* were identified. Finally, mixtures of conidia that were pre-stained with different fluorochromes indicated that *Foa* race 4 formed conidial anastomosis tubes (CATs) with *Foci*. *Foa* race 4 and *Foa* race 2, which are in different somatic compatibility groups, did not form CATs with each other.

**Conclusions:**

There was no evidence that *Foa* race 2 was involved in the recent evolution of *Foa* race 4; *Foa* race 2 and 4 are CAT-incompatible. Although *Foa* races 3 and 4 and the *Foci* are closely related, there is no evidence that either *Foci* contributed to the evolution of *Foa* race 4, or that *Foa* race 4 was the recent recipient of a multi-gene chromosomal segment from another strain. However, horizontal chromosome transfer could account for the major difference in the accessory genomes of *Foa* race 4 and the *Foci* and for their differences in host range.

## Background

The *Fusarium oxysporum* species complex (FOSC) contains thousands of clonal lineages. Individual strains typically cause disease in a limited number of plant hosts, which led to the *forma specialis* (f. sp.) designation, e.g., FOSC in f. sp. *apii* (*Foa*) cause disease in celery (*Apium graveolens var. dulce*) [1]. However, strains in the same *forma specialis* may be polyphyletic [2] as in the case of *Foa* races 2 and 4 [3]. The FOSC have a “multi-speed genome” with chromosomes that have conserved genes and fewer transposons, and “accessory” chromosomes that are often smaller in size, are transposon-rich, and harbor rapidly evolving pathogenicity factors [4]. Although the FOSC are asexual, they can acquire chromosomes from other strains [5].

What we now call *F. oxysporum* f. sp. *apii* (*Foa*) race 1 was recognized by the 1930’s as an economically important pathogen of the “yellow” celery cultivars that were grown at that time [6]; *Foa* race 1 isolates are virulent on cv. Golden Self Blanching but avirulent on “green” or Pascal-type celery cultivars such as Tall Utah 52–70 R Improved and Challenger [3]. However, observations of variation in culture morphology [6], vegetative (=somatic) compatibility groups [7], two-locus DNA sequences [2, 3] and high throughput sequencing [3] indicate that race 1 isolates are polymorphic. *Foa* race 2 was first reported in 1976 in California [8, 9] and subsequently spread to other production areas in North America in the 1980’s. *Foa* race 2 is virulent on both Tall Utah 52–70 R Improved and Golden Self Blanching, and isolates appear to be monomorphic [3]. In 1984, Puhalla [10] noted that in addition to *Foa* race 2, there was a *Foa* race 3 in California that was virulent on Tall Utah 52–70 R Improved but reportedly avirulent on Golden Self Blanching; importantly, *Foa* race 3 was in a different somatic compatibility group than *Foa* race 2. In a study that included pathogenicity tests and two-locus sequencing of isolates collected between 1993 and 2013 from symptomatic celery plants that were primarily from California, none of the 174 isolates were classified as *Foa* race 3 [3]. However one isolate from a culture collection that was deposited as the *Foa* “T” strain by California researcher Shirley Nash Smith in 1981 was classified as a “*Foa* race 3-type” [3].

Following the discovery of *Foa* race 2, resistance was identified in celeriac (*A. graveolens* var. *rapaceum*) and introgressed into celery [11]. The resulting commercial celery cultivars such as Challenger, Command, Green Bay, and Sabroso, have been the major tool for Fusarium yellows management since the early 2000’s. In 2013, race 4 of *Foa* was discovered in Camarillo in Ventura County, California; this race is highly virulent on the race 2-tolerant cultivars such as Challenger, the older cultivar Tall Utah 52–70 R Improved, and a variety of current cultivars in California [3]. Based on a 10 gene phylogeny and an analysis of 6898 single nucleotide polymorphisms from the core FOSC genome, Epstein et al. concluded that i) *Foa* is polyphyletic with *Foa* race 2 in FOSC Clade 3 and *Foa* races 1, 3, and 4 in FOSC Clade 2, and ii) the archaic and less virulent race 3 and the new highly virulent race 4 are very similar, suggesting that *Foa* race 3 may serve as a genomic control for an analysis of *Foa* race 4.

*F. oxysporum* f. sp. *coriandrii* (*Foci*)*,* which is pathogenic on coriander (=cilantro) but not on celery, was first reported in California in Santa Barbara County in 2005 [12]. Here, we further report that *Foa* race 4 has a broader host range than celery; it can also cause disease on coriander, although the *Foci* are more virulent on coriander than *Foa* race 4. After discovering that *Foci* are closely related to *Foa* races 3 and 4, we assembled high-quality genomes of *Foa* races 2, 3, and 4 and two isolates of *Foci*. We show evidence i) that *Foa* races 3 and 4 and the *Foci* are in a single somatic compatibility group and form a subclade with the FOSC Clade 2 based on 2718 aligned, conserved nuclear genes within the FOSC and the complete mitochondrial sequence; ii) that while the accessory genomes of *Foa* races 3 versus 4 are very similar, the *Foci* and the *Foa* in FOSC Clade 2 differ in approximately 37% of the accessory genome; and iii) that *Foa* race 4 apparently arose from a *Foa* race 3-like progenitor, and that neither the *Foci* nor *Foa* race 2 provided new DNA. Furthermore, we show that *Foa* races 2 and race 4 apparently do not form hetero-conidial anastomosis tubes, consistent with them being in separate species. We also identify i) previously undescribed effectors and pathogenicity factors that are up-regulated in *Foa* race 4 *in planta*; and ii) useful PCR primers for identifying *Foa* races 2 and 4 and the *Foci*.

## Results

### Assembled isolates

The isolates whose genomes were assembled are shown in Table 1. *Foa* race 1 was not sequenced because it is polymorphic and contemporary celery cultivars in the USA are not susceptible. Isolates that represent *Foa* races 2, 3, and 4 were described previously [3]. Two *Foci* isolates were sequenced: *Foci*3–2, which was from the same area in Ventura Co. as *Foa* race 4, and *Foci*GL306 which was the first reported *Foci* in California, and was isolated in 2004 from neighboring Santa Barbara County [12]. Further documentation on the collections are in Epstein et al. [3] and Additional file 1.

**Table 1:** The origin of *Fusarium oxysporum* isolates sequenced in this study

*forma specialis*	Race	Isolate name	Origin: City, County in California	Year collected	Original isolate ID
*apii*	*Foa* race 4	*Foa*R4	Camarillo, Ventura Co.^a^	2013	274.AC
*apii*	*Foa* race 3	*Foa*R3	Unknown^b^	Before 1981^b^	NRRL 38295
*apii*	*Foa* race 2	*Foa*R2	Santa Maria, Santa Barbara Co.^c^	2010	207.A
*coriandrii*	NA^d^	*Foci*3–2	Camarillo, Ventura Co.^e^	2016	3–2
*coriandrii*	NA	*Foci*GL306	Santa Barbara Co.^f^	2004	GL306

^a^GPS:34.212417, − 119.058611

^b^The isolate was submitted by California researcher Shirley Nash Smith as *F. oxysporum* f. sp. *apii* strain T to a culture collection

^c^GPS: 34.931793, −120.530553

^d^*NA* Not applicable. We are unaware of coriander cultivars with resistance

^e^GPS: 34.227416, −118.983724

^f^From Koike and Gordon [12]

### Pathogenicity characterization in celery and coriander

**Fig. 1:**
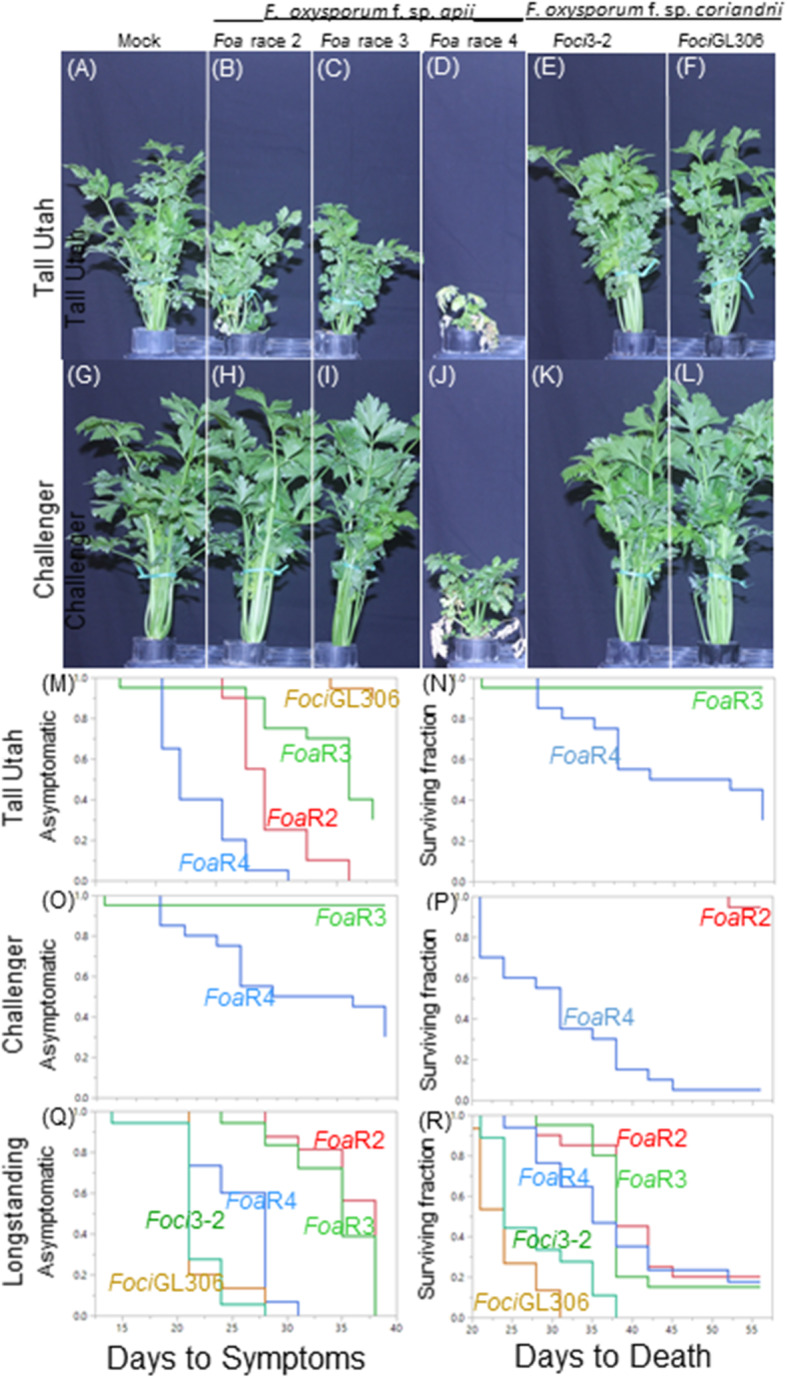
Virulence of strains of *F. oxysporum* f. sp. *apii* and f. sp*. coriandrii* on celery and coriander. **a**-**p** Two-month-old celery cultivars were transplanted into uninfested soil (mock) or soil infested with either *F. oxysporum* f. sp. *apii* (*Foa*) race 2 (*Foa*R2), *Foa* race 3 (*Foa*R3), *Foa* race 4 (*Foa*R4), or the *F. oxysporum* f. sp. *coriandrii* strains *Foci*3–2 and *Foci*GL306. (A-L) After 49 days, the median plant (*n* = 20) in height was photographed. (M-R) Kaplan-Meier plots of time to above-ground symptoms (left) and death (right) of the celery plants shown in either the photographs (**m**-**p**) or on coriander cv. Longstanding (**q**-**r**); coriander was direct-seeded. Treatments with 0% affected during the entire trial are not shown. Additional results from the same trial are shown in Additional file 3. For both days to symptoms and days to death, for each cultivar, *P* < 0.001 for the Log-Rank and Wilcoxon tests

The three differential celery cultivars were transplanted into either uninfested soil, or soil infested with either *Foa* race 2, 3 or 4 (Fig. 1 a-d and g-j, Additional file 2). *Foa* race 4 is highly virulent on all three cultivars. Cultivar Challenger is tolerant of *Foa* races 2 and 3 (Fig. 1 h-i and o-p) and Tall Utah 52–70 R Improved is susceptible to *Foa* races 2 and 3 (Fig. 1 b-c and m-n). Historically [13], *Foa* was described as causing symptoms on primary, i.e., celery, and several secondary hosts. Based on an assay of direct seeding of coriander into infested soil and completion of Koch’s postulates, coriander cv. Longstanding is a secondary host of *Foa* race 4; coriander is also a secondary host of *Foa* races 2 and 3, but these races are less virulent than *Foa* race 4 (Fig. 1 q-r, Additional file 3).

As reported previously [12], our results show that the *Foci* are pathogenic on coriander, but not on celery (Fig. 1 e-f and k-l, Additional file 3). The *Foci* are more virulent on coriander than any of the *Foa* races (Fig. 1 q-r). From the eight isolates from symptomatic coriander fields, seven were *Foci* (i.e., non-pathogenic on celery and pathogenic on coriander), and one (*Foa*R4V-7.5B) is a *Foa* race 4. This isolate had the pathogenicity phenotype of *Foa* race 4 and the *ef1* SNP variant that has so far only been found in one isolate of *Foa* race 4 from celery (*Foa*R4V-313–2.2).

### Whole genome sequencing and assembly

We performed PacBio (>50X) and Illumina (>95X) sequencing of DNA from *Foa* races 2, 3, and 4, and two *Foci* isolates. *Foa* race 4 was also analyzed with a Bionano optical map. Each assembly was completed to a similar level of contiguity, with most core chromosomes in a single contig, and accessory chromosomes in multiple fragments (Table 2; Additional file 4). Based on Benchmarking Universal Single-Copy Orthologs (BUSCO v2.0) with the Sordariomycota reference genes [14], all five of the genomes have > 98.7% of the expected single copy orthologs (Additional file 5) and meet or exceed the BUSCO parameters for quality of the *F. oxysporum* f. sp. *lycopersici* (*Fol*)4287 reference assembly (Genbank GCA_000149955.2 ASM14995v2).

**Table 2:** Statistics for the assembled *Fusarium oxysporum* f. sp. *apii* and f. sp. *coriandrii* genomes

*F. oxysporum forma specialis* / race	Isolate ID	No. contigs / scaffolds	Genome size, Mb	Maximum contig size, Mb	N_50_, Mb	Average PacBio RSII coverage	Illumina coverage (& technology^a^)	GenBank Assembly No.
*apii* race 4	*Foa*274.AC	74	67.4	7.0	4.4	70	120 (H), 35 (M)	JAAOOQ000000000
*apii* race 3	*Foa*R3^*b*^	76	65.4	7.0	4.0	72	107 (H)	JAAOOP000000000
*apii* race 2	*Foa*207.A	50	64.8	6.5	3.5	58	95 (H)	JAAOOO000000000
*coriandrii*	*Foci*3–2	50	65.5	6.7	5.0	52	329 (N)	JAAOON000000000
*coriandrii*	*Foci*GL306	51	65.1	6.8	5.0	50	273 (N)	JAAOOM000000000
*lycopersici*^c^	4287^d^	114	61.4	4.4	2.0	NA^e^	NA	GCA_000149955.2

^a^Paired-end reads were from ‘H’ (Hiseq 4000, 150 bp), ‘N’ (Novaseq, 150 bp), and/or ‘M’ (MiSeq, 250 bp)

^b^Synonym, NRRL 38295

^c^Used here as a reference genome [4, 16]

^d^Synonym, NRRL 34936

^e^*NA* Not applicable

### Phylogenetic analysis

**Fig. 2:**
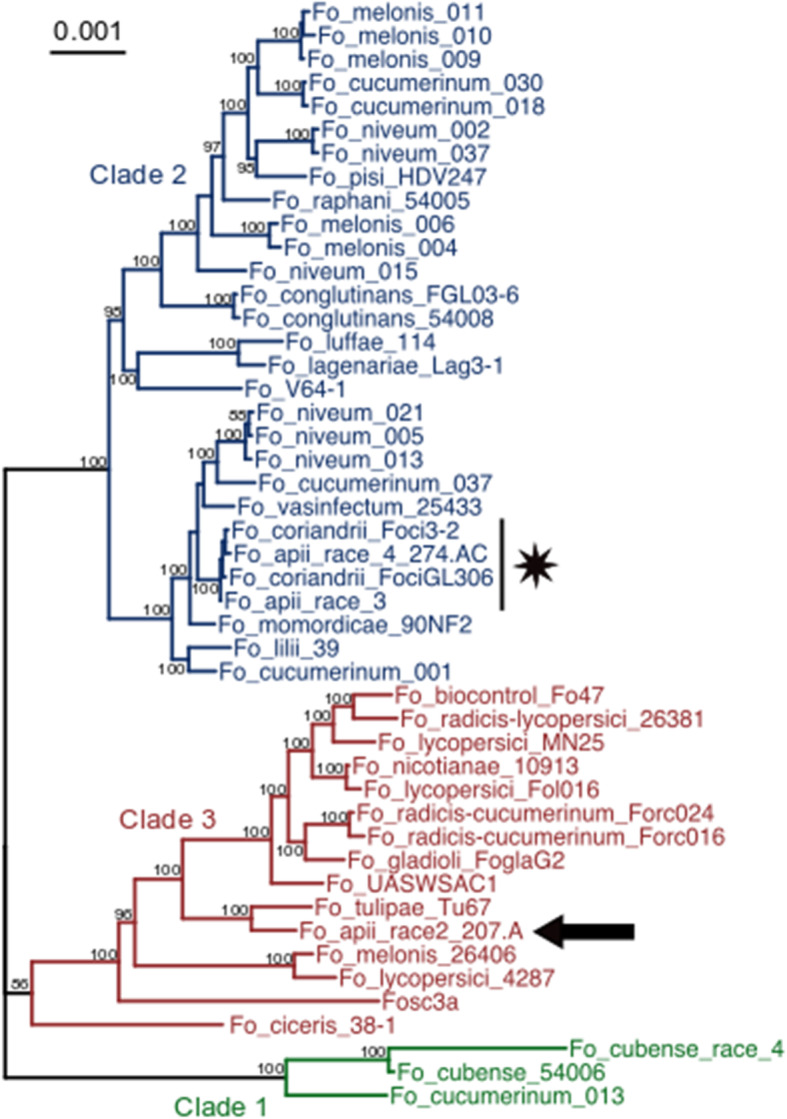
The phylogeny of the *Foa* and *Foci* strains within the FOSC. BUSCO v. 2.0 (Benchmark of Unique Single Copy Orthologs) was used to identify 2718 full-length, single copy genes that were in all strains. Sequences were aligned with MUSCLE and concatenated into a single ~ 5.5Mbp sequence. A phylogenetic tree was generated with RaxML with the general time reversible evolutionary model. Support for the tree is based on 1000 bootstrap replicates; bootstrap values below 70 are not shown. The branches corresponding to FOSC Clades 1, 3, and 2 are color coded with green, red, and blue, respectively. *Foa* race 2 is indicated with an arrow and the *Foa* races 3 and 4 and the *Foci* isolates are indicated with a star

Previously, using ten conserved genes in the FOSC, we placed *Foa* race 2 in FOSC Clade 3 and *Foa* races 1, 3 and 4 in FOSC Clade 2 [3]. Here, to further examine the relationship between our five, whole genome-sequenced strains and representative full-genome sequenced FOSC, we aligned 2718 BUSCO genes that were complete and single copy in all assemblies, concatenated the alignments, and generated a maximum likelihood phylogenetic tree. The two *Foci* strains and *Foa* races 3 and 4 are in a single, well-supported sub-clade (Fig. 2); the larger FOSC Clade 2 sub-clade that includes *Foci* and *Foa* races 3 and 4 also includes *F. oxysporum* f. sp. *vasinfectum* NRRL 25433.

We also compared the mitogenomes of our whole genome-sequenced strains to FOSC mitogenomes from Brankovics et al. [15]. Our five strains have a type I mitochondrial variable region [15] (Additional file 6); *Foa* races 3 and 4 and the two *Foci* strains contain ORF 2284. Remarkably, *Foa* races 3 and 4 and the two *Foci* strains have an identical 45,699 bp mitogenome haplotype. While these four mitogenomes are 97 to 99.97% identical to other FOSC Clade 2 with a type 1 variable region, to date, there are no other characterized mitogenomes with an identical haplotype. The 47,671 bp mitogenome of *Foa* race 2 is unique amongst the currently sequenced mitogenomes but has a 99.2–99.7% identity to the mitogenomes in other strains in FOSC Clade 3 that have a type I mitochondrial variable region.

### Core and accessory genome analyses

Based on the FOSC reference *Fol*4287 [16], which is in FOSC Clade 3, *F. oxysporum* have 11 core chromosomes (numbers 1, 2, 4, 5, and 7 through 13) with conserved genes and four accessory chromosomes that are highly variable between strains [4, 5]. We first used progressiveMauve software [17] to identify the contigs in the *Foa* and *Foci* strains that are part of core chromosomes (Additional file 4). In our five assemblies, 91% of the expected core chromosomes are represented by a single contig.

The assemblies indicate that there has been a major structural change to chromosomes within the *Foci* compared to *Foa* races 3 and 4. In both *Foci*3–2 and *Foci*GL306, the homologs of *Fol*4287 chromosomes 10 (3.1 M bp) and 11 (2.4 M bp) are in a single contig (6.2 and 6.1 M bp, respectively), suggesting that there was a fusion in just the *Foci* chromosomes. In contrast, chromosomes 10 and 11 assembled into separate contigs in *Foa* races 2, 3 and 4. To examine the fused region, we determined the chromosome juncture with progressiveMauve [17] and used Geneious to identify a 198 bp juncture region that was identical in both *Foci* strains and absent in the *Foa* strains. For both strains, read coverage in this region was >68X and > 47X for Illumina and PacBio, respectively. These data, combined with the observation that these chromosomes assembled identically at this locus in both *Foci* strains, support that a fusion event preceeded the emergence of the two clones.

Contigs with homology to a core chromosome frequently had transposon-rich regions at their ends that lacked homology with the *Fol*4287 reference. Consequently, we defined the conserved and accessory genome by marking the beginning and end of progressiveMauve alignments to core chromosomes (Additional file 4). All regions without homology to a *Fol*4287 core chromosome were classified as part of the accessory genome, and all contigs without homology to a core chromosome were classified as accessory contigs (Additional file 7).

Using pairwise average nucleotide distances (andi) [18] of core, accessory and total genomes, the data in Table 3 indicate that *Foa* race 2 is the most dissimilar strain compared to the four members of the FOSC Clade 2 (*P* < 0.0001). The accessory genomes are significantly more dissimilar than the core genomes (*P* < 0.001). The pair of *Foci* strains and the pair of *Foa* races 3 and 4 are more closely related to their *f. sp*. partner than they are to the other *f. sp*. (*P* < 0.001).

**Table 3:** ANchor DIstances between the complete, core and accessory genomes of the *Foa* and *Foci* strains^a^

Portion of genome^b^	Strain	*Fusarium oxysporum* strains			
		*Foa* race 2	*Foa* race 4	*Foa* race 3	*Foci*3–2
		Anchor distances on a scale from 0 (identical) to 1 (dissimilar)^c^			
Complete assembly	*Foa* race 4	1.69E-02			
	*Foa* race 3	1.69E-02	2.63E-04		
	*Foci*3–2	1.69E-02	1.03E-03	1.02E-03	
	*Foci*GL306	1.68E-02	1.04E-03	1.13E-03	1.37E-04
Core genome	*Foa* race 4	1.42E-02			
	*Foa* race 3	1.42E-02	1.94E-05		
	*Foci*3–2	1.43E-02	3.58E-05	4.11E-05	
	*Foci*GL306	1.42E-02	2.84E-05	2.97E-05	2.32E-05
Accessory genome	*Foa* race 4	2.62E-02			
	*Foa* race 3	2.66E-02	8.74E-04		
	*Foci*3–2	2.64E-02	3.62E-03	3.57E-03	
	*Foci*GL306	2.62E-02	3.54E-03	3.59E-03	3.79E-04

^a^ANchor DIstances (andi) are described in Haubold et al. [18]

^b^The core genome was identified by homologous colinear blocks [17] with the *F. oxysporum* f. sp. *lycopersici* 4287 reference. Portions of the genome that were not in the core were classified as the accessory genome

^c^To analyze the data with ANOVA, andi were log-transformed and analyzed by contrast analysis. Results are shown in the text

#### Identification of host-specific versus lineage-specific contigs in the accessory genome of *Foa* race 4 and *Foci*3–2

**Fig. 3:**
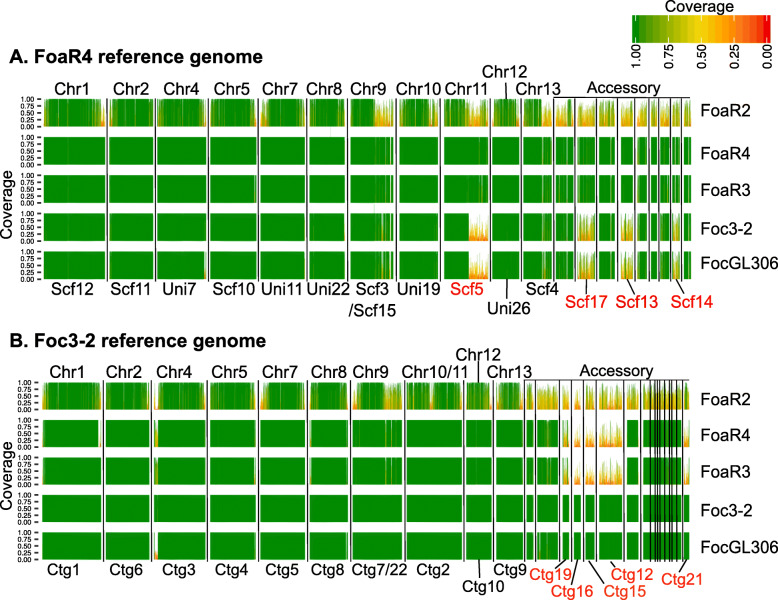
Coverage of *Foa* race 4 (*Foa*R4) and *Foci* 3–2 reference assemblies by Illumina reads from *Foa* and *Foci* strains. From each of the five strains, 6.5 Gbp (~100x coverage) of quality-filtered Illumina reads of each strain were mapped onto the *Foa* race 4 (**a**) and *Foci* 3–2 (**b**) reference assemblies. We calculated the proportion of coverage of each 10 kbp window in the reference assemblies. Here, only contigs with length greater than 150 kbp are shown, and are separated by vertical black lines. The darkest green sections of the histogram have a 100% coverage and the reddest sections have coverage close to 0%. Coverage of 0.5 (yellow) indicates that only 5 kbp of the 10 kbp segment had Illumina coverage. Contigs corresponding to core chromosomes are labeled above each plot. The IDs for contigs (Ctg) and scaffolds (Scf) that are associated with host specificity are lettered in red, below the plots. Each row corresponds to coverage from a single isolate, which is noted to the right of the graph (*Foa* race 2, *Foa*R2; *Foa* race 3, *Foa*R3). Additional files 8-9 have the quantification of the extent of Illumina-read coverage of each strain for each reference contig

We used a gene-independent, Illumina-mapping method to further examine the differences between the genomes, particularly in the accessory contigs in *Foa* race 4 versus *Foci*3–2. We selected *Foa* race 4 and *Foci*3–2 as reference strains, mapped filtered Illumina reads onto each contig, fragmented the references into 10 k bp segments, and then quantified the length of each segment with high-quality coverage. Compared to the *Foa* race 4 reference (Fig. 3 and Additional file 8), *Foa* race 3 has coverage over 99.7% of the genome, *Foci*3–2 and *Foci*GL306 have 94.5 and 94.2% coverage, respectively, and *Foa* race 2 has only 78.9% coverage. Compared to the *Foci*3–2 reference (Fig. 3 and Additional file 9), *Foci*GL306 has 99.2% coverage, *Foa* race 4 and *Foa* race 3 have 92.1 and 92.5% coverage, respectively, and *Foa* race 2 has only 78.2% coverage*.*

There are major diffences in some of the contigs in the accessory genomes in the *Foci* versus *Foa* races 3 and 4. In *Foa* race 4, the host-specific superscaffolds 17, 14, and 13, which represent 35% of the analyzed accessory genome, have less Illumina coverage in the *Foci* (from 49 to 57%) than from the *Foa* race 3 and 4 strains (from 87 to 95%) (Additional file 8). Similarly, in *Foci*3–2, the host-specific contigs 12, 15, 16, 19, 20, and 21, which represent 39% of the analyzed accessory genome, have less Illumina coverage from *Foa* races 3 and 4 (from 18 to 45%) than from the *Foci* (from 99 to 100%) (Additional file 9). Conservation of sequences on these contigs is associated, in the isolates tested, with host-specific differences.

#### Syntenic analysis

**Fig. 4:**
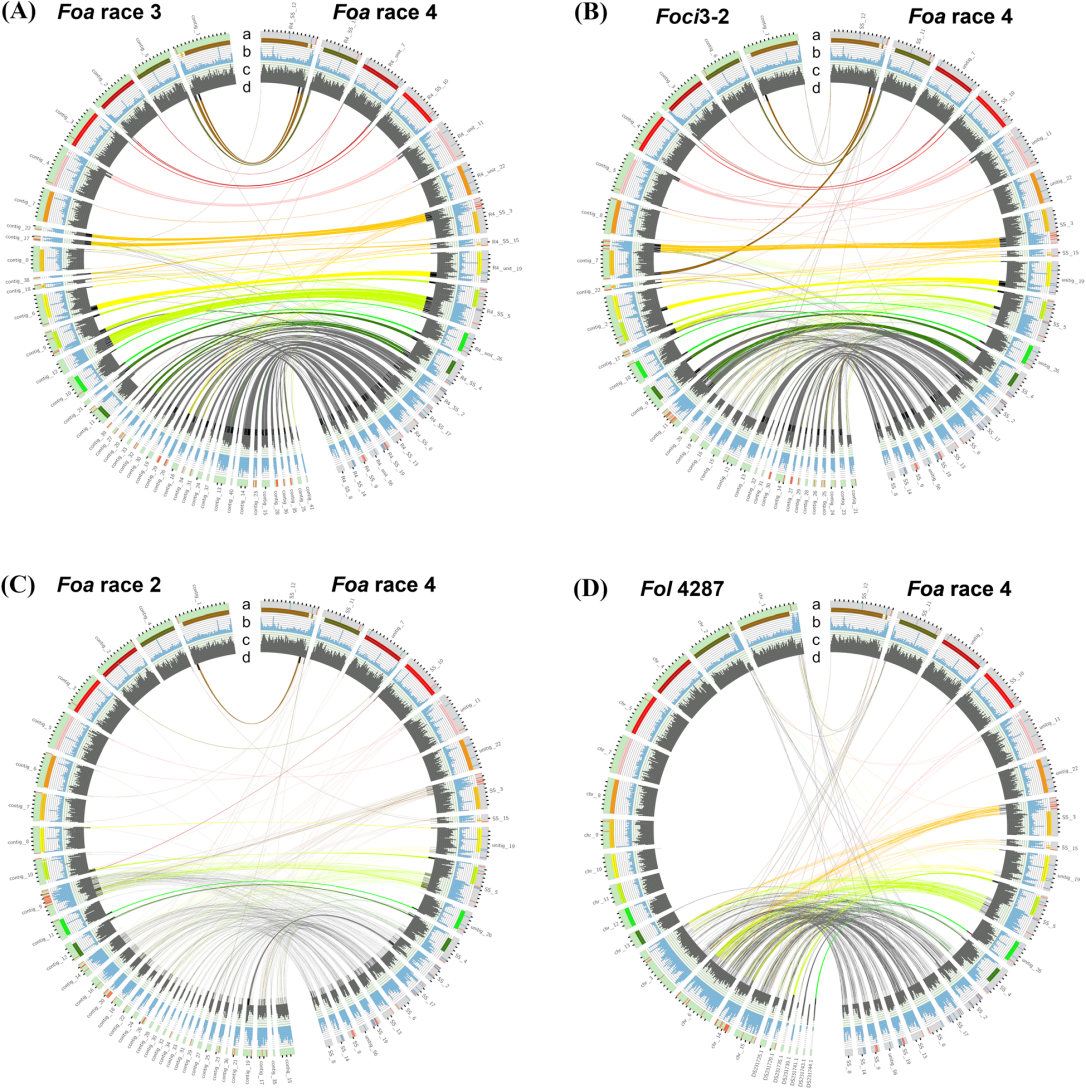
Synteny of the accessory genomes of *Foa* race 4 and other strains. Circos plot comparisons of “reciprocal best BLAST hits” (RBBH) homologs of genes in non-core (=accessory) regions of the *F. oxysporum* genome in *F. oxysporum* f. sp. *apii* (*Foa*) race 4 on the right side and on the left side *Foa* race 3 (**a**), *Foci*3–2 (**b**), *Foa* race 2 (**c**) and *Fol* 4287 (**d**). Contigs less than 150kbp are not shown. Non-core regions were determined using progressiveMauve with *Fol*4287 as a reference. Tic marks on ring a are 500 kb. Within ring a, red lines indicate miniature impala transposable elements (mimps), and blue lines indicate all genes with significantly (adjusted *P* < 0.05) increased expression *in planta* in celery crowns that were infected with *Foa* race 4 compared to *Foa* race 4 grown in vitro. In ring b, the solid colors within the upper portion denote a region with homology to one of the *Fol* core chromosomes. Genes within these regions are part of the core genome. Blue shows the density of repetitive elements with a full scale of 120 per 100 kb increment. In ring c, dark grey shows the density of gene models with a full scale of 50 per 100 kb increment. In ring d, the grey lines show genes in the accessory genome that have an RBBH with a > 80% identity over > 80% of the predicted nucleotide sequence. In the center, lines connect the RBBH in the accessory genome; genes connected by black lines are in accessory contigs and genes connected with other colors are in non-core regions of core chromosomes. The plots illustrate the most synteny between *Foa* races 4 and 3 in the accessory genome, less synteny between *Foa* race 4 and the *F. oxysporum* f. sp. *coriandrii* (*Foci*) strain 3–2, and the least synteny between *Foa* race 4 and either *Foa* race 2 or the f. sp. *lycopersici* reference *Fol*4287. Quantification of the number of homologs and their synteny are in Additional File 1

We used Circos (version 0.69.9) [19] to visualize the density of repetitive elements and gene models in the core and accessory contigs of our strains (Fig. 4, Additional files 10, 12A-C, and 13A-B). The core chromosomes have a higher gene density and a lower density of repetitive elements than the accessory regions of the genome. To examine synteny within the core genomes, we examined the BUSCO genes that were present as a full-length, single copy in each pairwise genome comparison. As shown in the Circos plots with comparisons of the BUSCO genes (Additional file 10A-D) > 96% of the genes are syntenic, even in pairs of a FOSC Clade 2 with a Clade 3 strain (Additional file 10C-D). As shown in Additional file 10D, the BUSCO genes are concentrated in only core chromosomes 1, 2, 4, 5, and 7 through 10; these contigs account for 60% of the length of the genome and contain 98.1% of the Sordariomycete BUSCO genes in *Foa* race 4. Core chromosomes 11, 12, and 13 account for 17% of the length of genome but only have 0.4% of the BUSCO genes and the accessory contigs only have 1.5% of BUSCO genes.

To compare the accessory (non-core) genomes between strains, we identified pairs of homologous gene models based on reciprocal best BLAST hit matching (with a minimum of an 80% reciprocal sequence identity and length). The Circos plots in Fig. 4 show the position of homologous genes in the accessory genomes. Of 6159 predicted genes in the accessory genome of *Foa* race 4, 64% had a homolog in the *Foa* race 3 accessory genome (Additional file 11). Of those, 90% were syntenic with *Foa* race 4. Fewer homologs of *Foa* race 4 accessory genes were identified in the accessory genomes of the *Foci* strains. For example, accessory genomes of *Foci*3–2 and *Foci*GL306 had homologs with 50 and 46%, respectively, of those in *Foa* race 4; of those, 84 and 82% were syntenic with *Foa* race 4. *Foa* race 2 and *Fol*4287 had the fewest homologs (15 and 14%, respectively), and of those, the least synteny (41 and 35%, respectively) with *Foa* race 4. The accessory genomes of the two *Foci* strains had the most similarity to each other (Additional files 9 and 13B-D); 75% of the accessory genes had homologs, and of those, 94% were syntenic.

**Fig. 5:**
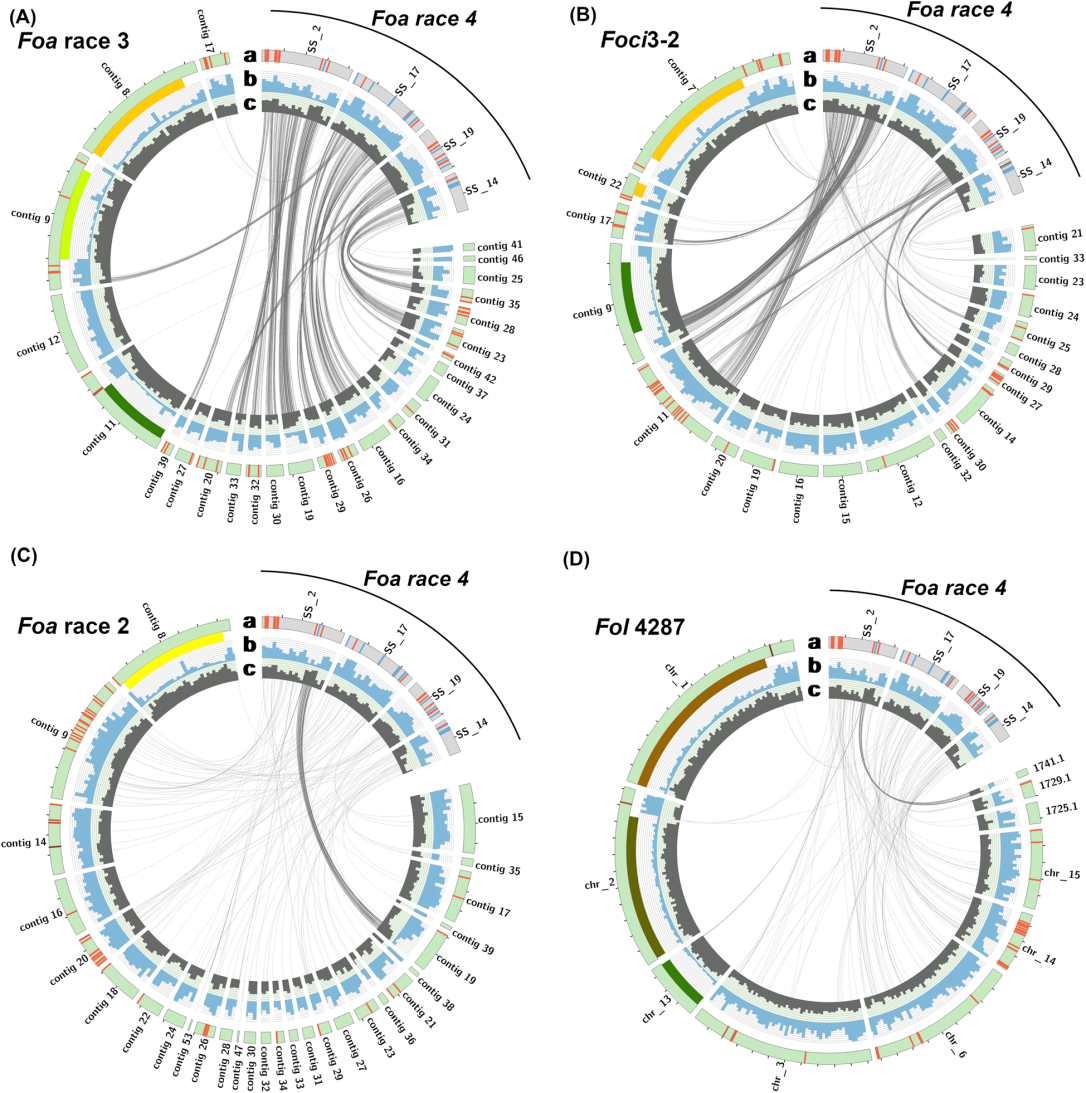
Synteny of gene models in four selected *Foa* race 4 accessory superscaffolds that have the most up-expressed genes *in planta* compared to in vitro*.* These four accessory contigs are shown on the top right, and contigs with homologous genes from the strain indicated in the top left are to the left and below in *Foa* race 3 (**a**), *Foci*3–2 (**b**), *Foa* race 2 (**c**) and *Fol* 4287 (**d**). Rings a-c are described in the legend for Fig. 4; the blue lines in ring a indicate all genes with significantly (adjusted *P* < 0.05) increased expression *in planta* in celery crowns that were infected with *Foa* race 4 compared to *Foa* race 4 grown in vitro*.* In the center, lines connect each “reciprocal best BLAST hit” (RBBH) with a > 80% identity over > 80% of the predicted nucleotide sequence. There are the following number of genes in each of the selected accessory *Foa* race 4 superscaffolds (SS): SS2, 523; SS17, 480; SS19, 320; and SS14, 204. In these selected accessory contigs, *Foa* race 3 (A) has homologs of 59 to 73% of these *Foa* race 4 gene models, depending on the contig (Additional file 14). *Foci*3–2 (B) has 70 and 58% of the homologs in the lineage-specific SS 2 and SS19 and only 8 and 25% of the homologs of the host-specific SS 17 and SS 14. Both *Foa* race 2 (C) and *Fol* 4287 (D), which are in FOSC Clade 3, have the fewest homologs, with only a total of 11 to 12% of the homologs in the four accessory contigs in *Foa* race 4

For a more focused comparison, we selected four accessory contigs in *Foa* race 4 with the most up-regulated genes *in planta* and visualized synteny of genes on just these contigs with their homologs in other strains (Fig. 5). The selected contigs were lineage–specific superscaffolds 2 and 19, and host-specific superscaffolds 17 and 14, which collectively had a total of 1527 gene models and represented 25% of the *Foa* race 4 accessory genome. Compared to *Foa* race 4, *Foa* race 3 has more homologs than any of the other strains, but only for 59 to 73% of the gene models (Additional file 14). *Foa* race 2 and *Fol*4287 had the fewest (5 to 20%) and similar percentages (i.e., within three percentage points) of homologs in each of the four superscaffolds. Consistent with the quantification of read coverage on accessory contigs (Fig. 3), *Foa* race 4 and either *Foci* strain had fewer homologous genes on host-specific contigs SS17 and SS14 than on lineage-specific contigs SS2 and SS19. In particular, the *Foci* strains had homologs for fewer than 9% of genes on SS17 and 25% of genes on SS14, whereas the *Foci* strains had homologs for between 41 and 70% of genes on SS2 and SS19.

We visualized the synteny and conservation of predicted genes in *Fol*4287’s pathogenicity chromosome 14 with genes on any contigs from *Foa* race 4 and *Foci*GL306 (Additional file 15). *Foa* race 4 and *Foci*GL306 have homologs of 28 and 23% of genes on chromosome 14, respectively, but they are distributed over 15 contigs in *Foa* race 4 and 22 contigs in *Foci*GL306. Although some genes from this pathogenicity chromosome appear to have homologs in *Foci* and *Foa* races 3 and 4, the distribution of homologs indicates that any horizontal transfer was ancient.

### Transcriptomic analysis and identification of predicted effectors

We quantified gene expression via TagSeq [20] using 3′ QuantSeq mRNA libraries (Lexogen, Inc.) prepared from *Foa* race 4 during plant infection (*in planta*) and growth in liquid media (in vitro) with *Foa* races 2, 3, and 4*.* From 51 to 52% of the approximately 20 thousand predicted genes were expressed in vitro for each isolate (Additional file 7). With *Foa* race 4, 76 genes were expressed significantly less *in planta* (adjusted *P* < 0.05) and 80 genes were expressed significantly more *in planta* than in vitro*.* Amongst the 80 up-regulated *Foa* race 4 genes *in planta*, 23 were house-keeping genes that are unlikely to be specifically associated with virulence per se (data not shown), and 22 genes accounted for < 0.1% of the *Foa* race 4 counts *in planta*, and consequently were classified as lower priority for review. The 35 remaining genes of interest (Table 4) included 23 putative effectors (Additional file 16, GenBank MT364384-MT364418).

**Table 4:** Thirty-five up-expressed potential effectors or pathogenicity/virulence factors in *Foa* race 4 *in planta*^a^

	EdgeR analysis of 3’TagSeq				Predicted protein			Genome location		Postulated function^f^
Gene	Avg. cpm^b^ *in planta*	Avg. cpm in vitro	LogFC	Adjusted *P-*value	Cellular local-ization^c^	Protein mass, kDa	No. cyste-ine resi-dues	Lineage-specific (LS) Accessory, host-specific (HS) Accessory, or Core chromosome no.^d^	Contig: start bp^e^	
NS.09678^g^	25,300	2284	2.46	2.5E-07	secr	12.8	3	Core chr13, OUT	SS4:3084010	Effector
NS.05815	18,471	1.2	13.2	1.3E-08	secr	30.0	8	HS Acc	SS14:228927	SIX1 effector
PGN.06282^g^	18,379	0.1	15.4	4.5E-09	secr	5.6	0	HS Acc	SS17:11497	Effector
NS.06742	14,139	5.3	10.0	2.2E-09	secr	29.0	8	LS Acc	SS19:499478	Effector
NS.14450	12,854	598	3.20	1.7E-04	intra	3.6	5	Core chr10, W/IN	uni19:1108440	Unknown, but not secreted
NS.06525^g,i^	11,596	3.1	10.7	1.4E-09	secr	12.8	3	HS Acc	SS17:1662847	Effector
PGN.05952	9116	2.7	10.5	4.5E-09	secr	12.5	6	HS Acc	SS14:222108	Effector
NS.05812^g^	7873	5.3	9.5	2.1E-10	intra	15.2	0	HS Acc	SS14:223943	Unknown, but not secreted
PGN.20363	7809	0.4	12.95	4.8E-08	secr	10.8	8	Core chr4, W/IN	uni7:4951240	Effector
PGN.05922	7745	36	6.5	1.4E-09	secr	11.9	6	HS Acc	SS14:122611	Effector
PGN.15680	6730	57	5.65	1.5E-09	secr	12.4	8	Core chr10, W/IN	uni19:3727811	Effector
PGN.06635^g^	6133	0.2	13.5	5.5E-10	secr	13.8	6	HS Acc	SS17:1461842	Effector
NS.06362^g^	5038	0.1	13.2	2.0E-08	secr	9.9	6	HS Acc	SS17:1000217	Effector
PGN.09917	4632	480	2.23	1.3E-08	secr	12.8	7	Core chr13, OUT	SS4:3084950	Effector
NS.05829	4407	0.3	12.1	6.4E-08	secr	30.5	8	HS Acc	SS14:278932	SIX1 effector
PGN.07042	4099	1.1	10.7	1.2E-09	secr	13.7	6	LS Acc	SS19:912650	Effector
NS.06821^g,h^	3935	0.1	13.1	7.1E-09	intra	31.3	0	LS Acc	SS19:758783	Abhydrolase_1
NS.10798	3622	158	3.33	5.5E-06	secr	45.2	12	Core chr11, OUT	SS5:2865460	Metalloproteinase
NS.17257	2979	2.3	9.17	2.5E-10	secr	51.2	3	Core chr12, W/IN	uni26:1845597	Amine oxidase or dehydrogenase
PGN.06691^g,i^	2938	0.0	13.0	4.6E-09	secr	12.5	7	HS Acc	SS17:1661252	Effector
NS.12180^j^	2830	14.0	6.5	6.0E-08	intra	28.0	3	Acc	uni111:57177	Transposase
NS.01422	2533	121	3.01	8.6E-05	secr	25.2	7	Core chr5, W/IN	SS10:4596446	Effector
NS.09643	2463	0.3	11.07	5.4E-08	secr	29.3	8	Core chr13, OUT	SS4:2943454	Effector; *F. oxysporum* f. sp. *vasinfectum* Pep1 homolog
NS.16793	2461	0.2	11.61	1.5E-07	secr	18.2	14	Core chr12, W/IN	uni26:670796	Effector
NS.06528	2038	55.7	3.8	1.6E-07	secr?	15.1	9	HS Acc	SS17:1671928	Effector
NS.06820^h^	1874	0.1	12.3	4.0E-08	intra	33.9	3	LS Acc	SS19:758056	Alcohol dehydrogenase GroES-like domain & NAD(P) binding (Panther)
PGN.06376^g^	1768	0.2	11.6	1.9E-08	secr	12.3	8	HS Acc	SS17:415228	Effector
NS.14958	1743	1.6	8.82	1.3E-07	TM	69.0	11	Core chr10, W/IN	uni19:2687638	Polyol transporter
NS.07235	1665	45.3	4.1	2.3E-07	intra	81.2	3	LS Acc	SS2:1462982	Catalase/peroxidase
NS.06359	1636	1.0	9.4	2.6E-09	secr	73.2	12	HS Acc	SS17:990399	Extracellular glucosidase
NS.17886	1589	4.5	6.1	5.7E-04	Intra/nuclear	106.7	9	LS Acc	uni56:301050	Unknown, but not an effector
NS.15045	1545	95	2.93	4.0E-08	secr	28.8	0	Core chr10, W/IN	uni19:2886040	Effector; related to early nodulin 75 precursor
NS.11338^k^	1262	2.7	6.7	8.5E-04	intra	119.6	11	LS Acc	SS6:142150	Retrotransposon
PGN.05959	1060	0.8	9.4	8.2E-08	secr	9.8	4	HS Acc	SS14:239989	Effector
PGN.06650	1040	0.0	11.6	4.6E-09	secr	12.3	8	HS Acc	SS17:1511396	Effector

^a^Genes 1) had significantly (adjusted *P* < 0.05) higher expression *in planta* in celery crowns than in vitro; 2) accounted for more than 0.1% of the total fungal reads *in planta* and 3) were not “house-keeping” genes

^b^cpm, normalized as the number of reads per million fungal reads

^c^intra, intracellular, based on WoLF PSORT and the absence of a secretion signal and a transmembrane domain; secr, secreted based on SignalP-5.0; secr?, based on the absence of a secretion signal by SignalP but an extrascellular localization by WoLF PSORT; TM, transmembrane based on Geneious TMHMM prediction

^d^Based on comparisons with progressiveMauve of *Foa* race 4 with the *Fol* 4287 reference. Contigs were categorized as follows: core, on contigs that are homologs of *Fol* chromosomes; or Acc, accessory contigs (no homology to a core chromosome). Genes in core chromosomes were further designated as W/IN, within the core region, i.e., within a progressiveMauve colinear block, or OUT, outside of the core region, i.e., between colinear blocks

^e^*SS* Superscaffold; *uni* Unitig

^f^DNA and predicted amino acid sequences were BLASTed on GenBank and analyzed with Geneious InterProScan and Panther. Genes that were identified as putative effectors were secreted (SignalP-5.0, 2), had a molecular mass < 35 kDa, and have no known biochemical function

^g^The terminus of a miniature impala (mimp) transposable element is within 2.5 kb upstream of the ORF start

^h^NS.06820 and NS.06821 are adjacent to each other

^i^PGN.06691 and NS.06525 are adjacent to each other

^j^The translated gene model was annotated by Panther as a Tc1_like DDE_3 and is contained within a RepeatMasker-annotated TcMar-Tc1

^k^The translated gene model was annotated by Panther as an integrase/ribonuclease Gag-Pol related retrotransposon/DNA-RNA polymerase with reverse transcriptase_2. The gene model is contained within a RepeatMasker-annotated 5614 bp Long Terminal Repeat/Copia transposon that is bounded by 95% identical 140 bp terminal repeats

These putative effectors were predicted to have a signal peptide with a cleavage site, an extracellular localization, a relatively small molecular mass (< 35 kDa with a median of 12.8 kDa), and a median of 7 cysteine residues (Table 4). Accessory contigs contained 65% of these predicted highly-expressed effectors. All have an identical DNA sequence in *Foa* races 3 and race 4, but 65% have a duplication within *Foa* race 4 compared with race 3 (Additional file 16). *Foa* race 2 had homologs for 74% of these genes, although none had identical sequences to those found in *Foa* race 4. Forty-three percent of these predicted effectors had an identical sequence in both *Foci* isolates and *Foa* race 3 and 4.

For potential effectors associated with pathogenicity on celery, there are four predicted effectors (PGN.05952 and PGN.15680/PGN.06376/PGN.06650) that are present in all three *Foa* races and absent in the *Foci.* Of these, the predicted 122 aa protein in *Foa* race 2 is 93% identical to the *Foa* race 4 PGN.05952, and based on the NCBI nr database (as of 25 Aug. 2020), 97% identical to a predicted protein from *F. oxysporum* f. sp. *melonis* 26406 [21]. The 114 aa predicted protein of the other three putative effectors in *Foa* race 4 (PGN.15680/PGN.06376/PGN.06650) are 97–99% identical; each has an identical predicted protein in *Foa* race 3. The single *Foa* race 2 predicted protein is 96 and 94% identical to a hypothetical protein of *F. oxysporum* f. sp. *conglutinans* [22] and f. sp. *cepae* [23], respectively.

The only *Secreted in Xylem* (*SIX*) effector homolog with evidence of expression in *Foa* race 4 was *SIX1* (NS.05815 and NS.05829, GenBank MT364385 and MT364398) [24–27]. Based on the genome assemblies, all the *Foa* strains have two *SIX1* orthologs, whereas the *Foci* strains have only one (Additional file 17). Both *SIX1* homologs were significantly up-regulated *in planta;* no expression was detected in vitro in the three *Foa* that were assayed (Data not shown). The two *SIX1* orthologs in *Foa* race 3 and 4 are similar distances apart (46,973 and 50,005 bp, respectively), which suggests the ancestral *SIX1* was duplicated and inverted in the same chromosome. There is evidence of divergence after duplication; the two orthologs share 86% of nucleotide sequence identities. Based on the GenBank non-redundant and *Fusarium oxysporum* “wgs” database, the FOSC Clade 2 *Foa SIX1* have unique DNA sequences, with a maximum identity of 89% with other FOSC strains.

A curious association between effectors and miniature impala (*mimp*) non-autonomous, Class II transposable elements (TEs) and has been observed in FOSC *formae speciales lycopersici*, *melonis*, *cucumerinum*, and *niveum* [28, 29]. Remarkably, a total of seven effectors (of 23 up-regulated and highly expressed *in planta*) were within 2.5 kbp downstream of a *mimp* in the *Foa* race 4 reference assembly. Six of these effectors were located on lineage-specific Superscaffold 17, and were within 1.1 kbp of 8 of the 14 *mimps* identified on this scaffold (Additional file 16). Superscaffold 17 has 480 predicted genes in a length of 1.9 Mbp; the six *mimp*-associated effectors are distributed over 1.6 Mbp. The other effector in proximity to a *mimp* was on Superscaffold 4. The two *SIX1* homologs were not within 2.5 kbp of a mimp.

### Two-locus haplotypes and PCR primers for diagnosis

Except for *Foa* race 1 isolates, the *Foa* and *Foci* isolates in our collection (Additional file 18) were in three, *ef1/igs* haplotypes. First, the single isolate of *Foa* race 3, forty-five of forty-six isolates of *Foa* race 4 from celery, and seven of eight isolates from coriander have an identical two-locus haplotype (GenBank Accessions *ef1*: FJ985371.1, KX619213.1, KX619215.1, KX619220.1-KX619227.1, KX619229.1; *igs*: FJ985604.1, KX619387.1, KX619389.1, KX619394.1-KX619401.1, KX619403.1). Second, the other isolate of *Foa* race 4 (*Foa*R4V-313–2.2) and one of the isolates from coriander (*Foa*R4V-7.5B) differ by a single common SNP in an *ef1* intron (GenBank Accessions MT295485, MT295484). As of 13 February 2020, none of entries in the Fusarium MLST (http://fusarium.mycobank.org/) and as of 30 September 2020, none of the *F. oxysporum* in the *Fusarium* spp. whole genome databases in GenBank at NCBI (Additional file 19) and only one f. sp. *melonis* (strain NRRL 22518, GenBank FJ985265 and FJ985447.1) in the nt database have the identical two-locus sequence of either the major or single-SNP variant haplotype. Third, the twenty-two pathogenic isolates of *Foa* race 2 have an identical haplotype. Each of the *Foa* race 1 isolates had a unique two-locus haplotype and were never implicated as the primary pathogen in a contemporary California cultivar.

Four new primer pairs were designed that can be used to identify either the *Foa* race 2 haplotype or *Foa* race 4 and/or *Foci* (Additional file 20). The primers can be used on DNA extracts from either cultures or from infected crown tissue. We did not develop primers for *Foa* race 3 because we have never isolated this strain from symptomatic celery [3].

Both empirical and in silico tests indicate that our previous *Foa* race 4 primers (NS3875–2) and our new *Foa* race 4 primers (FOAR4–447) cross-react with off-target FOSC strains (Additional files 18 and 20). However, because each individual primer pair amplifies different off-target isolates, they can together be used as a barcode to identify *Foa* race 4. The only observed false positive for this combination is *Foci* isolate 10T.

Most *Foci* isolates can be identified by amplification with the new FOCI-g_c31 and FOCI2–21 primers. Again, the exception to this test is *Foci* isolate 10T, which amplifies with both the older (NS3875–2) and the new FOAR4–447 *Foa* race 4 primers. It is important to note that we collected *Foa* race 4 from diseased coriander plants in one field and show that *Foa* race 4 causes disease on coriander. Therefore, if an isolate from coriander is positive for both *Foa* race 4 primers and negative for both *Foci* primers, a pathogenicity test is required to differentiate between a *Foci*10T-type and a *Foa* race 4.

We identified a new and more specific primer pair (FOAR2-76 k) that can be used to identify the *Foa* race 2 haplotype. Previously [3], we used pathogenicity testing on celery cultivars Tall Utah 52–70 R Improved and Challenger and *ef1*/*igs* two-locus sequencing to identify 22 *Foa* race 2 isolates that were amplified with the previous primer pair N4851 for *Foa* race 2. All these isolates were amplified with FOAR2-76 k. We also identified 18 isolates that had been isolated from celery with symptoms of Fusarium yellows that had the same *ef1*/*igs* two-locus sequence as *Foa* race 2 (e.g. GenBank KX619102.1 and KX619276.1), but were non-pathogenic on both celery cultivars; they also amplified with both the older and the new *Foa* race 2 primers. Three of the non-pathogenic isolates were collected before 1994; loss of pathogenicity in *Foa* during storage is common. We postulate that the other 15 non-pathogenic FOSC with the *Foa* race 2 haplotype are also isolates that have lost pathogenicity. None of the other 66 FOSC strains that we tested empirically or the 437 whole genome-sequenced FOSC at NCBI were amplified with FOAR2-76 k and none have an *ef1*/*igs* two-locus haplotype that is identical to *Foa* race 2.

### Evaluation of hyphal and conidial anastomosis compatibility

Because we have isolated *Foa* race 2 and race 4 from the same celery plant (data not shown), we assessed whether the two strains could anastomose via either hyphae or conidial anastomosis tubes (CATs). Hyphal anastomosis compatibility was tested by pairwise combinations of *nitM* and *nit1* mutants of *Foa* races 2, 3, and 4 and the two *Foci* strains (3–2, and GL306). The results indicate that *Foa* races 3 and 4 and the *Foci* strains are in the same somatic compatibility group, and that *Foa* race 2 is in a different somatic compatibility group.

**Fig. 6:**
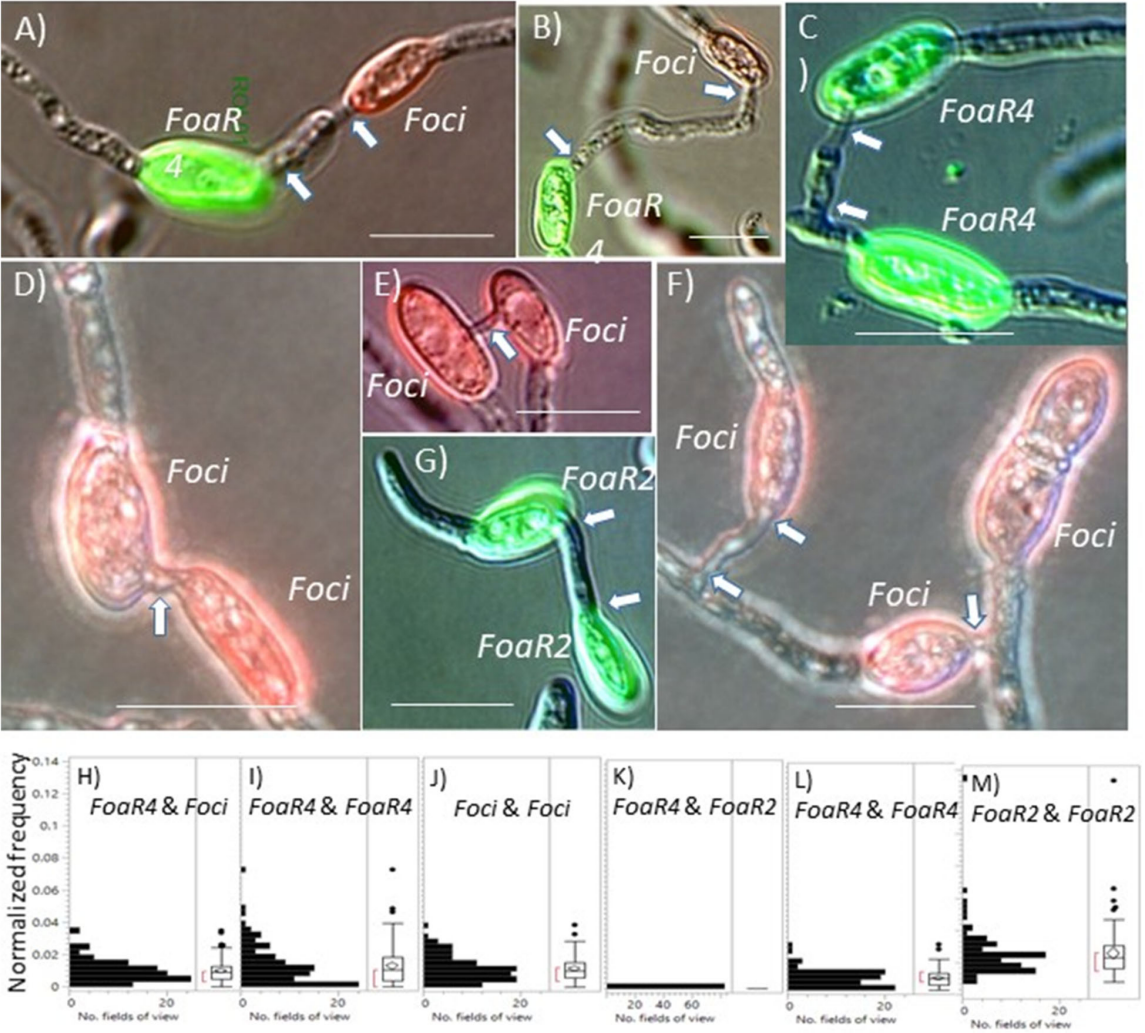
Homo- (within strain) and hetero-(between strain) conidial anastomosis tubes**. a**-**g** Micrographs. *F. oxysporum* f. sp. *apii* race 4 (*FoaR4*) conidia were pre-stained with wheat germ agglutinin that was conjugated to either Alexa Fluor 488 or Alexa Fluor 594 (not shown) and then mixed with differently labeled *F. oxysporum* f. sp. *coriandrii* (*Foci*)3–2 or *F. oxysporum* f. sp. *apii* race 2 (*FoaR2*). After conidia were incubated on polystyrene in conditions conducive for conidial anastomosis tube (CAT) formation, a Leica with epifluorescent GFP and rhodamine filters, and a 63X objective with differential interference contrast optics, was used to record the images. Bars represent 10 μm. Short CATs are indicated with a single arrow and longer CATs are indicated with an arrow at each end of the CAT. **a**-**b** *Foa*R4 and *Foci* form hetero-CATs. **c**-**g** *FoaR4*, *FoaR2* and *Foci* form homo-CATs. Hetero-CATs between *FoaR4* and *FoaR2* were never observed. **f** Three conidia form a CAT cluster. **h**-**m** Frequency of formation of homo- and hetero-CATs in mixtures of strains. **h**-**j** *Foa*R4 and *Foci.* **k**-**m** *Foa*R4 and *Foa*R2. CATs that could be categorized as hetero- or homo-CATs were recorded, as were the numbers of each of the labeled conidia in the same field of view. For each field of view in which there was at least one score-able CAT, the normalized frequency of the CAT category was computed to adjust for the number of possible pairs of each type. Data from three independent trials with similar results are shown here; a total of 741 and 695 CATs were evaluated in (**h**-**j**) and (**k**-**m**), respectively. A non-parametric Kruskall-Wallis test indicates that normalized frequencies of homo-*Foa* race 4, homo-*Foci*, and hetero-CATs of *Foa* race 4 and *Foci* were statistically indistinguishable (*P* = 0.41). In contrast, *Foa* race 4 did not form CATs with *Foa* race 2

To examine formation of conidial anastomosis tubes (CATs), *Foa* race 4 conidia that were pre-stained with wheat germ agglutinin that was conjugated to either Alexa Fluor 488 or 594 were mixed with a differently-stained strain, either *Foci*3–2 or *Foa* race 2. All three tested strains (*Foa* race 4, *Foci*, and *Foa* race 2) form “homo” CATs (Fig. 6). According to the non-parametric Kruskal-Wallis test, the normalized frequencies of hetero-CATs between *Foa* race 4 and *Foci* and both the homo-CATs were statistically indistinguishable (*P* = 0.41), i.e., *Foa* race 4 and *Foci* form hetero-CATs as readily as either does with its own strain. In contrast, in mixtures with *Foa* races 4 and 2, hetero-CATs were never observed either when a total of 695 CATs were scored or when CATs were examined but not recorded. In a Kruskall-Wallis analysis of the normalized frequencies of the *Foa* races 4 and 2 hetero- and homo-CATs, there were highly significant differences (*P* < 0.0001) between the strains; the non-parametric Steel-Dwass multiple comparison test found highly significant differences (*P* < 0.0001) between all pairs, i.e., there were highly significantly fewer (apparently non-existent) hetero-CATs than either homo-CATs, and there were highly significantly more homo-CATs of *Foa* race 2 than homo-CATs of *Foa* race 4. Consequently, *Foa* race 4 and *Foci* readily form hetero-CATs, but *Foa* races 4 and 2 are CAT-incompatible. We note that while most CATs are formed between two conidia, we also observed homo- and hetero-CAT clusters with more than two conidia. Figure 6f shows a homo-CAT cluster in which the middle conidium is connected to two other conidia; the conidium on the left has formed a CAT with the middle conidium and the middle conidium has formed a CAT with the conidium on its right.

## Discussion

Here, we release the first DNA assemblies of *F. oxysporum* f. sp. *apii* (*Foa*) races 2, 3, and 4 and *F. oxysporum* f. sp. *coriandrii* (*Foci*) genomes. *Foa* races 3 and 4 and *Foci* form a here-to-fore unexplored sub-clade of the FOSC Clade 2; they are in the same somatic compatibility group. This is apparently the first example within the FOSC of two *formae speciales* that are in the same somatic compatibility group. *F. oxysporum* f. sp. *apii* were amongst the first plant pathogenic fusaria that were recognized by Wollenweber in 1917 (as *F. orthoceras*) and were codified as a *F. oxysporum forma specialis* by Snyder and Hansen in 1940 [1]. In the U.S., *Foa* race 2 has been an economically important pathogen of celery since the mid-1970’s; highly susceptible varieties are no longer grown. Currently in California, *Foa* race 4 in celery and *Foci* in coriander have the characteristics of emerging infectious plant diseases: the pathogens are spreading, yield losses can be severe, and there are no economical solutions for their control. We propose that the disease caused by *Foa* race 4 be called Fusarium wilt of celery in order to better describe the disease and to differentiate it from *Foa* race 2, causal agent of Fusarium yellows on celery.

We speculate that *Foa* race 4 evolved recently for the following reasons: the disease first appeared in a single field in approximately 2011 [3]; this highly virulent strain has never been reported elsewhere; and it is closely related to, but distinct from, another strain (*Foa* race 3) that we obtained from a culture that presumably originated in California.

Because the apparently new and hyper-virulent *Foa* race 4 is overall highly similar and syntenic with the archaic *Foa* race 3, we speculate that *Foa* race 4 arose from a *Foa* race 3-like strain, which may have arisen from one of the polyphyletic *Foa* race 1 strains. Although horizontal chromosome transfer has been demonstrated in *F. oxysporum* f. sp. *lycopersici* [30], our analyses of short read coverage across contigs (Fig. 3, Additional files 8-9), synteny of gene models with Circos plots (Figs. 4-5 and Additional file 12), and colinear blocks with progressiveMauve (not shown), do not support the conclusion that the virulence of *Foa* race 4 is due to the acquisition of an entire chromosome from either *Foa* race 2 or a *Foci*. Nonetheless, we cannot rule out the possibility that *Foa* race 4 acquired a smaller DNA fragment from a member of the FOSC or another microorganism.

Our bioinformatic analyses suggest that horizontal chromosome transfer was a key evolutionary event in the separation of the *Foci* from *Foa* races 3 and 4. *Foa* races 3 and 4 and the *Foci* have highly similar DNA in the core genome and are all in the same somatic compatibility group. However, in accordance with horizontal chromosome transfer, the *Foci* differ from these *Foa* overall in ca. 37% of the accessory genome. Thus, we postulate that either these *Foa* and/or the *Foci* have acquired one or two different host-specific chromosomes via horizontal chromosome transfer [4, 30]. These chromosomes would have originated in isolates that were not sequenced in this study.

*F. oxysporum* CATs can be formed between strains in two different somatic compatibility groups [31], and we investigated whether *Foa* race 4 formed CATs with *Foa* race 2. *Fusarium oxysporum* can form microconidia in xylem vessels [32], and if a plant is co-infected by two strains (such as we have observed with *Foa* race 2 and race 4), conidial anastomosis tubes (CATs) could be a portal for DNA exchange between these somatically incompatible isolates [33]. First, we demonstrated that we could pre-label microconidia with wheat germ agglutinin (WGA) conjugated with one of two fluorophores, mix conidia with the two different fluorophores, allow the conidia to form conidial anastomosis tubes, and then score CATs as either homo-CATs (between two conidia of the same strain) or hetero-CATs (between two conidia of different strains). Second, we demonstrated that *Foa* race 4 and *Foci*3–2, which are in the same somatic compatibility group, form hetero-CATs as readily as either strain forms homo-CATs. Therefore, both hyphal and CAT fusion could enable DNA exchange between *Foa* races 3 and 4 and *Foci* in nature. As far as we know, this is the first demonstration of CATs between two strains within FOSC Clade 2. Third, we demonstrated that *Foa* races 4 and 2, which are in different FOSC clades, do not form hetero-CATs in conditions in which they form homo-CATs (Fig. 6).

Shahi et al. [31] showed that CATs were formed between two somatically incompatible strains (*F. oxysporum* f. sp*. lycopersici* strain 4287 and the biocontrol strain *F. oxysporum* Fo47), which are both in FOSC Clade 3. They and others have suggested that heterokaryon incompatibility is at least partially suppressed during CAT fusion in *F. oxysporum* and *Colletotrichum lindemuthianum* [34]. However, our results indicate that barriers to CAT-mediated nuclear exchange exist within the FOSC, at least between strains in different FOSC clades. This CAT-incompatibility between a strain in FOSC Clades 2 and 3 is further evidence that these FOSC clades are indeed different species [2].

The potential for gene flow between distinct *formae speciales* complicates efforts to develop durable tools for molecular identification. In addition to hyphal and CAT compatibility, these *Foa* and *Foci* share a common geographic range, and celery and coriander crops are commonly rotated in the same fields in California. We have detected genetic variation within the *Foci* that may suggest that genetic exchange between these phenotypically defined groups has occurred in nature. Specifically, in contrast to six of the *Foci* (including the two whole genome sequenced strains), one of the *Foci* (*Foci*10T) has an *ef1/igs* haplotype that is associated with a minor *Foa* R4 variant, is positive for the intended *Foa* race 4 marker FOAR4–447 and negative for the intended *Foci* markers FOCI2–21 and FOCI-g_c31 (Additional files 18 and 20). This observation is most easily explained by gene flow between these *formae speciales*; chromosomes carrying the marker sequences, but not all the genes required for virulence on celery, may have been transferred. Perhaps as a consequence, we were not able to develop a test that specifically differentiates all *Foci* from *Foa* race 4*.*

In addition to causing disease in the greenhouse, *Foa* race 4 can cause disease of coriander in the field. That is, coriander is a secondary, symptomatic host of *Foa* race 4. However, *Foa* race 4 was recovered from only one out of seven coriander fields surveyed, presumably because *Foa* race 4 is less virulent than *Foci* on coriander.

Both the two hosts (celery and coriander) and pathogens (*Foa* races 3 and 4 and the *Foci*) are closely related. Celery and coriander are both in the subfamily Apioideae in the family Apiaceae. This suggests that these closely related hosts may share susceptibility factors, as has been postulated elsewhere [35], and that co-evolution between host and pathogen may have occurred in these pathosystems. Future characterization of the mechanisms of resistance by celery to *Foci* and pathogenicity determinants in these *formae speciales* is necessary to test this hypothesis.

This is the first examination of putative effectors in the *Foa* and *Foci*. Notably, the *Foa* in both FOSC Clade 2 (races 3 and 4) and FOSC Clade 3 (*Foa* race 2) have two *Secreted in Xylem 1 (SIX1*) homologs but the *Foci* only have one. Both *SIX1* homologs are highly expressed in the crowns of *Foa* race 4-infected celery but are essentially not expressed when any of the three *Foa* races are grown in vitro. Other FOSC strains have multiple *SIX1* homologs [25], which are virulence factors in multiple *formae speciales*, including *F. oxysporum* f. sp. *lycopersici* [36], f. sp. *conglutinans* [26], and f. sp. *cubense* tropical race 4 [37]. In f. sp. *lycopersici*, *SIX1* also serves as an avirulence gene in eliciting host defense in tomatoes with the *I-3* gene [24]. Other *formae speciales* that have a *SIX1* homolog include strain Fo5176 in Arabidopsis [38], *betae* in sugar beet [39], *canariensis* in date palm [40], *lini* in flax [29], *melonis* in melon [41], *pisi* in pea [42], *cepae* in onion [43], and *physali* in cape gooseberry [44]. The conservation of *SIX1* in many pathosystems suggests that either directly or indirectly it targets a conserved plant protein. Interestingly, *SIX1* in *Foa* race 4 and the *Foci* are on host-specific accessory contigs. In addition to *SIX1*, all of the other 23 up-regulated and highly expressed putative effectors shown in Table 4 have homologs in other FOSC strains (Additional file 16).

Interestingly, four of the 23 putative effectors identified in *Foa* race 4 (PGN.05952 and a family containing PGN.15680, PGN.06376, and PGN.06650) have homologous DNA in *Foa* races 2 and 3, that is absent in the *Foci* assemblies (Additional file 16); the two homologs in Foa race 2 are located in a single accessory 3.3 Mb chromosome-sized contig (no. 9). Whether these two effectors in particular are involved in pathogenicity in celery remains to be determined [45]. Regardless, van Dam et al. [41] provided support for the hypothesis that even polyphyletic *f. sp.* share distinctive effector profiles. Here, we note that, based on DNA homology at the NCBI GenBank whole genome database for *Fusarium* spp. (Additional file 19), there is 100% identity of the *Foa* race 4 homolog with strains of the multiple ff. spp.: PGN.05952 and f. sp. *spinaciae*; and PGN.15680 and f. sp. *niveum*. PGN.06376/PGN.06650 has greater than 98% identity with strains in f. sp. *lini*, *vasinfectum*, *niveum*, and *albidinis*. This suggests that there may be an active shuffling of pangenomic DNA across *ff. spp*. in the FOSC [46] that are CAT-compatible; although individual FOSC strains only cause disease in a narrow host range, the FOSC can infect “non-hosts” [35] and consequently could share DNA in environments where CAT can form.

Using transcriptomics, we detected increased expression of two putative transposons in *Foa* race 4 *in planta* (NS.12180 and NS.11338 in Table 4); both were amongst the 35 selected genes that had significantly increased expression *in planta* than in vitro, accounted for > 0.1% of the cDNA counts *in planta*, and did not encode for a “housekeeping gene.” The quantity of TEs (Additional file 21) particularly in the accessory genome (as shown in the Circos plots) suggest that they are involved in the evolution of all the *Foa* and *Foci*. There is also evidence of expression of other transposons in vitro (data not shown). Consistent with the observed association of *mimps* and promoter regions of effector genes in *Fol*4287 pathogenicity chromosome 14 [47], we show that in *Foa* race 4 superscaffold 17, six of eight putative effectors that are up-regulated *in planta* have one or more mimps within 1.1 kbp of the start codon. Whether *mimps* are active and important in these FOSC remains to be determined.

## Conclusions

We report the first full-genome assemblies of *F. oxysporum* f. sp. *apii* (*Foa*) races 2, 3 and 4 and two strains of the related *F. oxysporum* f. sp. *coriandrii* (*Foci*), each with fewer than 75 contigs*. Foa* races 3 and 4 (which are primarily pathogenic on celery) and the *Foci* (which are only pathogenic on coriander) are in the same somatic compatibility group; both celery and coriander are in the plant family Apiaceae. Our bioinformatic and biological comparisons lead to the following conclusions. 1) Consistent with Epstein et al. [3], *Foa* is polyphyletic with race 2 in FOSC Clade 3 and races 3 and 4 in FOSC Clade 2, i.e., *Foa* races 2 and 4 are comparatively unrelated. 2) Based on analyses of both the core and accessory genomes, the older and less virulent *Foa* race 3 is a useful genomic control for the new and highly virulent *Foa* race 4, i.e., *Foa* race 4 presumably arose from a *Foa* race 3-like progenitor, although apparently not from our *Foa* race 3 isolate. 3) *Foa* races 3 and 4 and the *Foci* are in a well-supported FOSC Clade 2 sub-clade based on the fact that they are in the same somatic compatibility group, share a mitochondrial DNA haplotype, and have highly similar core genomes. However, within the FOSC Clade 2 *Foa*-*Foci* subclade, the *Foa* and the *Foci* have distinguishable accessory genomes, and, based on total length, differ in approximately 37% of the accessory contigs. Consequently, horizontal chromosome transfer of a pathogenicity chromosome is presumably responsible for the main difference between *Foa* race 4 and the *Foci*. 4) Although the literature [31] indicates that two *F. oxysporum* strains that are in different somatic compatibility groups within the same FOSC can form conidial anastomosis tubes (CAT) that allows chromosome transfer, *Foa* race 2 and *Foa* race 4, which are in different somatic compatibility groups, do not form hetero-CATs, but do form homo-CATs. Thus, in accordance with a lack of evidence of transmission of *Foa* race 2 alleles into *Foa* race 4, there may be no mechanism of horizontal chromosome transfer across FOSC Clades from *Foa* race 2 into a progenitor of *Foa* race 4. 5) Although *Foa* race 4 and *Foci* are CAT-compatible, there is similarly no bioinformatic evidence that novel *Foci* alleles contributed to the evolution of *Foa* race 4. More generally, there is no current bioinformatic evidence that *Foa* race 4 was the recipient of a relatively large chromosomal segment from another strain. 6) Based on significantly increased expression *in planta* vs. in vitro with RNA TagSeq of *Foa* race 4, we identified 23 putative effectors and 12 other pathogenicity factors including two presumably active transposons; two of the putative effectors are encoded by *Secreted in Xylem* (*SIX1*) genes. 7) We selected and verified diagnostic PCR primers for *Foa* races 2 and race 4.

## Methods

### Isolate culture and storage conditions

Celery and coriander plants with symptoms of *F. oxysporum* infections were collected and taken to the laboratory. Symptomatic plants with visible rotting were not sampled to avoid isolation of secondary organisms. The geographic origin of the sequenced isolates is indicated in Table 1. After *F. oxysporum* was cultured from symptomatic plant tissue, cultures were single-cell purified and stored as described previously [3].

### Virulence tests

To produce *F. oxysporum* inoculum for soil infestation, millet seeds were hydrated overnight. One hundred cc of drained seeds per 500 ml flask were autoclaved, and then infested with either plugs from one-week-old cultures grown on potato dextrose agar (PDA) or dried conidia that had been stored on filter paper. Cultures were incubated under approx. 5000 lx cool-white fluorescent lights at 22 °C for 8 to 10 days; cultures were shaken vigorously every other day for more uniform colonization of the substrate.

For uninfested planting media in the greenhouse, we mixed steam-sterilized University of California Davis greenhouse soil (GHS) as a 3:1 (v/v) mix of perlite: GHS. The perlite: GHS mix was placed in either 10 cm diam pots or 6.4 cm diam planting tubes. For celery in infested soil, the bottom ¾ of pot or tube was filled with the perlite: GHS mix and the upper ¼ was filled with a thoroughly mixed preparation of a 1:15 (v/v) ratio of inoculum to the perlite-GHS mix. Uninfested controls had neither inoculum nor millet seeds.

Celery seeds were obtained from the following: Golden Self Blanching and Tall Utah 52–70 R Improved from Burpee Seed Co. (Warminster, PA, USA) and Challenger from Syngenta (Woodland, CA, USA). Two-month-old celery was transplanted into the infested soil. Golden Self Blanching is an heirloom yellow cultivar, Tall Utah 52–70 R Improved is a green Pascal-type cultivar, and Challenger was first marketed in 1999 as a *Foa* race 2-resistant, Pascal-type cultivar. In keeping with agricultural practice, coriander cv. Longstanding (Ferry Morse, Fulton, KY, USA) was direct-seeded. For coriander, we used the same perlite: GHS mix, but used a uniformly infested soil with a 1:60 (v/v) ratio inoculum to the perlite: GHS mix throughout the entire pot. Germination occurred after 8 to 10 days. All plants were maintained in a greenhouse that was maintained between 27 and 29 °C. Foliar symptoms were recorded weekly.

Harvested plants were washed and scored for typical vascular discoloration on a 0 to 5 severity scale: 0, asymptomatic: 1, some discoloration in the lateral root vasculature; 2, some discoloration in the main root vasculature; 3, some discoloration in the crown vasculature; 4, extensive discoloration of the crown vasculature; and 5, plant dead. Based on the mock-inoculated controls, isolates with a mean of < 1.0 were rated as nonpathogenic. To confirm that symptoms were caused by pathogens, Koch’s postulates were completed on the isolates that were not previously characterized in Epstein et al. [3].

### Two locus sequencing

Isolates that were not previously sequenced in the *ef1* and IGS rDNA amplicons were Sanger-sequenced as described previously [3]. New NCBI GenBank accession numbers for *ef1* are MT295484-MT295492 and for IGS are MT295475-MT295483.

### High throughput sequencing

Total genomic DNA was purified as described previously [48]. Illumina libraries were prepared and sequenced by either the University of California at Davis DNA Technologies Core Facility or the Michigan State University Genomics Core Facility. Library quality was confirmed before sequencing using the Agilent 2100 Bioanalyzer (Agilent Techonologies). The Illumina platform used for sequencing each isolate is listed in Table 2. PacBio SMRTbell libraries were prepared at the University of California at Davis DNA Technologies Core Facility and sequenced on the PacBio RSII platform. Sequence coverage is indicated in Table 2.

### Genome assemblies

#### *F*. *oxysporum* f. sp. *apii* (*Foa*) race 4

PacBio RSII reads were assembled with the HGAP3 pipeline in smrtanalysis v2.3.0 with default parameters and an estimated genome size of 50 Mb. This initial assembly had an average coverage of 70.2X and contained 135 contigs. Two hundred fifty bp paired-end Illumina MiSeq reads (35X) coverage [3] were initially used for error correction. These reads were trimmed to a minimum Phred score of 20 with Trimmomatic (v0.33), mapped to the assembly with Bowtie2, and indels/snps were corrected with Pilon v1.18 (as specified by the “--fix bases” parameter).

To correct the assembly, Bionano optical mapping was conducted by the Luo Lab (Plant Sciences, UC Davis) using the Irysview (version 2.5.1) software. First, the sequence assembly was used to generate an in silico map of target sites for BspQI and BssSI restriction endonucleases. A complete double digestion had a predicted 116,009 DNA molecules greater than 150,000 bp in length (The N50 for molecule length was 253.3 k bp). The experimentally-generated optical map had a total length of 97.25 M bp and an N50 of 0.74 M bp. The sequence-based and optical maps were aligned to identify chimeric sequences and to scaffold un-assembled contigs. This comparison revealed 6 chimeric sequences that were disassembled into 13 contigs, and 59 unassembled contigs that were stitched into 16 scaffold sequences.

For further error correction, we removed 23 contigs that were likely to be sequencing artifacts: contigs with low (< 19X) coverage with PacBio reads compared to the assembly average of 70X; no evidence for existence in the optical map; and less than 55 kbp in length. We next performed an additional error correction by mapping 150 bp paired-end Illumina Hiseq reads (~120X coverage) with Bowtie 2 (using ‘--end-to-end’ alignment only) and Pilon (version 1.18) error correction. The mapping/Pilon error correction process was repeated four times until few new errors were identified.

#### Other genome assemblies

PacBio RSII reads of *Foa* races 2 and 3 and *Foci*3–2 and GL306 were assembled by Falcon (version 0.4.2). The resulting assemblies were polished with PacBio reads using the quiver consensus module smrtanalysis (version 2.3.0). Additional error correction was conducted by mapping 150 bp paired end Illumina reads to assemblies with Bowtie 2 (using ‘--end-to-end’) and polishing with Pilon (version 1.18). This process was repeated four times.

### Annotation

Genomes were annotated with CodingQuarry (version 2.0) using gene models pre-trained on the *Fol*4287 version 2 genome with in vitro and *in planta* RNAseq reads [41, 49]. SignalP (version 5.0) was used to predict secretion signals, and WoLF PSORT was used to confirm extracellular localization. Transposons were annotated by Repeatmodeler (version 1.0.11) and RepeatMasker (version 4.0.8) [50]; results are summarized in Additional file 21. Miniature impala (*mimp*) transposable elements were annotated by TIRmite (version 1.1.3) using four profile hidden Markov models built from terminal inverted repeats of mimps identified using the regular expression pattern “..CAGTGGG..GCAA[TA]AA” (https://github.com/Adamtaranto/TIRmite). A script for running TIRmite for *mimp* discovery is provided at https://github.com/SamuelBrinker/Repertoire_v6.

### Phylogenetic analyses

The following FOSC assemblies from GenBank (https://www.ncbi.nlm.nih.gov/) and the designation in Fig. 2 are as follows: GCA_001702695.2, Fo_radicis-cucumerinum_forc016; GCA_000259975.2, Fo_lycopersici_MN25; GCA_001757345.1, Fo_ciceris_38–1; GCA_000260075.2, Fo_pisi_HDV247; GCA_000260155.3, Fo_radicis-lycopersici_26,381; GCA_000260175.2, Fo_vasinfectum_25,433; GCA_000260195.2, Fo_cubense_54,006 (synonym, *F. odoratissimum*); GCA_000260215.2, Fo_conglutinans_54,008; GCA_000260235.2, Fo_raphani_54,005; GCA_002318975.1, Fo_melonis_26,406; GCA_000271705.2, Fo_biocontrol_Fo47; GCA_000271745.2, Fosc_3a; GCA_000350365.1, Fo_cubense_race 4 (synonym, *F. odoratissimum*); GCA_000733055.2, FoUASWSAC1; GCA_003315725.1, Fo_lycopersici_4287; GCA_001702495.1, Fo_cucumerinum_013; GCA_001702505.1, Fo_niveum_005; GCA_001702515.1, Fo_cucumeriunum_001; GCA_001702545.1, Fo_cucumerinum_018; GCA_001702615.1, Fo_cucumeriunum_030; GCA_001702635.1, Fo_cucumeriunum_037; GCA_001702725.1, Fo_radicis-cucumerinum_ forc024; GCA_001702745.1, Fo_niveum_002; GCA_001702775.1, Fo_niveum_013; GCA_001702795.1, Fo_niveum015; GCA_001702845.1, Fo_niveum_037; GCA_001702865.1, Fo_niveum_021; GCA_001702995.1, Fo_lycopersici_016; GCA_001703215.1, Fo_melonis_004; GCA_001703255.1, Fo_melonis_006; GCA_001703265.1, Fo_melonis_009; GCA_001703295.1, Fo_melonis_011; GCA_001703305.1, Fo_melonis_010; GCA_002233795.1, Fo_momordicae_90nf2; GCA_002233805.1, Fo_tulipae_Tu67; GCA_002233895.1, Fo_gladioli_FoglaG2; GCA_002234045.1, Fo_nicotianae_10913; GCA_002234105.1, Fo_luffae_114; GCA_002234115.1, Fo_lilii_39; GCA_002234135.1, Fo_lagenariae_31; GCA_002711405.2, Fo_conglutinans_FGL03–6; and GCA_900096695.1, Fo_V64. Simão et al. [14] selected 3735 single copy BUSCO genes in Sodariomycetes. We selected 2718 single copy, full-length orthologues that were present in all strains, aligned the sequences with MUSCLE (version 3.8), concatenated the genes into a single, ~ 5.5 Mbp sequence, and then used RAxML (version 8.2.12) to generate a maximum likelihood phylogeny using the general time reversible evolutionary model with gamma correction and 1000 bootstrap replicates [51, 52].

For phylogeny of the mitochondrial DNA (mitogenome), we used 35 annotated mitochondrial sequences from Brankovics et al. [15] with *F. commune* JCM11502 (LT906348.1 in GenBank) as an outgoup. Brankovics et al.’s [15] four FOSC clade 1 strains and their GenBank mitogenomes are as follows: f. sp. *cubense* race 4 II5 (=NRRL 54006) (LT906347.1) and B2 (LT571433.1); f. sp. *cucumerinum* Foc013 (LT906309.1) and f. sp. *vasinfectum* Fov24500 (LT906346.1). The 19 FOSC Clade 2 reference strains and their GenBank mitogenomes are as follows: f. sp. *conglutinans* race 2 PHW808 (=NRRL 54008) (LT906357.1); f. sp. *cubense* race 1 N2 (LT906350.1); f. sp. cucumerinum Foc001 (LT906307.1), Foc030 (LT906313.1), Foc035 (LT906314.1), and Foc037 (LT906315.1); Fom006 (LT906327.1), Fom009 (LT906328.1), Fom010 (LT906329.1), Fom011 (LT906330.1); f. sp. *niveum* Fon002, (LT906334.1), Fon005 (LT906335.1), Fon013 (LT906337.1), Fon015 (LT906338.1), Fon019 (LT906339.1), Fon021 (LT906341.1); f. sp. *pisi* NRRL 37622 (LT906354.1); f. sp. f. sp. *raphani* NRRL 54005 (= PHW815) (LT906356.1) and f. sp. *vasinfectum* NRRL 25433 (LT906351.1). The 10 FOSC Clade 3 reference strains and their GenBank mitogenomes are as follows: f. sp. *lycopersici* DF023 (LT906301.1), Fol016 (LT906319.1), race 24,287 (=NRRL 34936) (LT906324.1), race 3 NRRL 54003 (= MN25) (LT906355.1); f. sp. *melonis* (NRRL 26406) **(**LT906353.1**)**; f. sp. *radicis-cucumerinum* Forc016 (LT906342.1), f. sp. *radicis-lycopersici* NRRL 26381 (=CL57)(LT906352.1), and the *F. oxysporum* NRRL 54002 (= Fo47) biocontrol strain from soil (LT906306.1), the UASWS AC1 strain **(**LT906358.1), and the FOSC3-a human pathogenic strain (LT906345.1). To assemble the mitogenomes of our three *Foa* and two *Foci* strains, we extracted the mitogenomes from the Illumina assemblies [15] using the Geneious 11.1.5 software. In order to detect artifacts in the assembly associated with the expectation of a linear rather than a circular DNA molecule, we mapped the Illumina reads to the mitogenome, and circularized the DNA starting at the ‘ATG’ start codon of the *nad2* gene. To determine if an apparent SNP within a homopolymer of 11A’s was an artifact, we amplified the homopolymer that preceeded *nad2* with primers FOSC-pre-nad2-HomoP 5’AGAATTCGATTTTCTCCTAAGGCTCGC3′ (forward) and 5’ACCACCGTGTAAACCTACTCCTTTAGT3′ (reverse). We used Mafft 1.3.7 in Geneious Prime 2020.0.4 to align the sequences and manually checked the alignment. A phylogenetic tree was generated in Geneious with RaxML with the general time reversible evolutionary model GAMMA. Rapid bootstrapping and search for the best-scoring maximum parsimony tree was done with 1000 bootstrap replications.

### Identification of core and accessory contigs

For our five assemblies (Table 2), we identified homologs of the 11 core *Fol* 4287 chromosomes (Genbank GCA_003315725.1) with progressiveMauve [17]. Contigs, or portions of contigs, that did not share a colinear block with the *Fol* 4287 reference, were classified as either accessory contigs or non-core regions on a core chromosome, respectively.

### Pairwise genomic comparisons of the *Foa*, *Foci* and *Fol* 4287 reference strains

Average nucleotide distances of the core and accessory genomes (as identified by progressiveMauve) were computed by andi [18]. andi values were log-transformed and analyzed by contrast analysis in ANOVA. Circos (version 0.69.9) was used to visualize synteny and specific genomic features [19]. Synteny was quantified using a program at https://github.com/objetora/lepstein. After the selection of pairs of homologous genes, each contig of the reference strain was sorted by locus. Then, each reference contig was broken into blocks with a constant target contig. A target gene in that block was considered syntenic if it belonged to either an ascending or a descending run of at least three target genes within the block.

### Identification of strain-specific regions in *Foa* races 3 and 4 and *Foci*3–2 by read mapping

Raw Illumina reads were filtered for quality with the following HTStream (version 1.0.0; https://github.com/ibest/HTStream) functions: hts_SuperDeduper (to remove PCR duplicates); hts_AdapterTrimmer (to remove adapter sequences); hts_SeqScreener (to remove adapter and phiX sequences), hts_QWindowTrim (to trim read ends with a PHRED quality value less than 20), and hts_NTrimmer (to keep only reads with no Ns). Only reads that were greater than 90 bp were retained (−M 90). From the filtered reads, 6.5 Gbp (~100x coverage) were aligned to the reference genome with BWA MEM (version 0.7.17-r1188) [53, 54]. Read coverage of the alignments was calculated with Bedtools ‘genomecov’ function (version 2.29.0), and regions with coverage less than 10x were discarded [54]. The Bedtools ‘coverage’ function was then used to calculate the proportion of bases with coverage for each 10kbp window in the genome. Coverage was visualized in R (version 3.5.2) using the package ggplot2 [55, 56]. To quantify an overall Illumina-coverage as a proxy for genome similarity, we calculated a weighted average of the coverage relative to the reference strain; the calculations were based on the contigs shown in Additional files 8 and 9.

### Differential expression experiments and analysis

For in vitro treatments, five replicates of *Foa* race 2, 3 and 4 were grown in 0.17% yeast nitrogen base without amino acids, 3% sucrose and 100 mM potassium nitrate at 100 rpm at 27 °C for 72 h. For each of five replicates, eight celery cultivar Tall Utah 52–70 R Improved plants were either transplanted into uninfested soil or soil infested with *Foa* race 4 and incubated for 21 days. Crown tissue was first coarsely ground with a pestle at the greenhouse in RNAlater (ThermoFisher Scientific, Waltham MA) at 4 °C and then finely ground in a liquid N_2_-cold mortar with 5 volumes of sterile sand and 20 mg polyvinylpolypyrrolidone/crown. The RNA was extracted at 65 °C in a RNAase-free buffer with 3% CTAB, 100 mM Tris-HCl (pH 8), 1.4 M NaCl, 20 mM EDTA, 5% polyvinylpyrrolidone, and (freshly added) 1.4% mercaptoethanol [57]. RNA was purified in chloroform:isoamyl alcohol and chloroform, precipitated in lithium chloride, and washed in 75% ethanol. DNA was digested with a TURBO DNA-*free* kit (ThermoFisher Scientific). RNA was quantified with Qubit and integrity was assessed with an Agilent Bioanalyzer. The University of California at Davis DNA Technologies Core Facility prepared 3’ QuantSeq mRNA libraries (Lexogen, Inc.) using a protocol with 14 PCR cycles [20]. At the same facility, the five replicate samples/treatment were 90 or 100 bp single-end sequenced on a HiSeq 4000. Raw reads were quality filtered with HTStream as previously described with minor modifications: PCR duplicates were not removed, and we used hts PolyATTrim to trim poly-A/T regions from the beginning or ends of reads. Filtered reads were aligned to the respective fungal reference genome with STAR (version 2.7.0) with a maximum allowed intron size of 6000 bp [58]. A 1000 bp UTR feature was added to the 3’ end of each gene using a custom python script (https://github.com/bnjenner/Publications/blob/master/Global_Fof_Henry_2020/TAGseq_gtf_annotation/tag_annotation.py). Read counts per gene were calculated by htseq-count (HTSeq version 0.6.1) from both CDS and UTR features.

For the in vitro replicates, there were 4.6 + 0.4 (SEM), 5.0 + 0.2, and 4.4 + 0.4 million sequenced reads for races 2, 3, and 4, respectively. However, because approx. 99.6% of the *in planta* reads were celery (data not shown), we performed additional sequencing of one of the *in planta Foa* race 4 replicates so that there 8.4 X 10^5^ mapped reads; the other four replicates had 1.7 + 0.2 X 10^4^ mapped reads. For *Foa* race 4, differential expression was calculated in pairwise comparisons between in vitro and *in planta* samples with ‘EdgeR’ [59]. First, all annotated genes were filtered to include only those with sufficient counts for statistical analysis using three counts per replicate in the in vivo samples and the (function = filterByExpr). The remaining genes were Voom transformed (function: voom) and fit to a model matrix (function: model.matrix). For each gene, contrasts were calculated between in vitro and *in planta* samples (function: makeContrasts) and analyzed using the contrasts fit function. The ‘eBayes’ function was used for empirical Bayes smoothing of standard errors.

Because the QuantSeq-identified, up-regulated, *in planta* gene PGN.06282 was only present in the *Foa* race 4 assembly, we designed PCR primers (5’CCATAGGCTTAGAAAGGTAAGTC3’ and 5’TTTCTTCAGTGGTCTCACTATG3’), and found an amplicon in *Foa* race 3; we then discovered that the *Foa* race 4 PGN.06282 was present in the raw reads (but not the final assembly) of *Foa* race 3.

### *Foa* race 2 and race 4 and *Foci* diagnostic PCR assays

We identified potential targets for diagnostic primer design with the ‘novel region finder’ function in Panseq [60]. *Foa* race 2, *Foa* race 4 and the *Foci* were compared with all genomes included in the whole genome phylogenetic tree. Potential diagnostic loci were then manually compared to sequences on GenBank in the whole genome shotgun database for *F. oxysporum* and the non-redundant database. Primers were designed with the Primer3Plus software (primer3plus.com*)*, analyzed in silico, and empirically tested with DNA from the following (Additional file 18): Six *Foa* race 1, twenty-two *Foa* race 2, one *Foa* race 3 type, twelve *Foa* race 4, eight pathogenic isolates from coriander, one non-pathogenic isolate that had the same two-locus haplotype as a *Foa* race 1 isolate, 18 non-pathogenic isolates that had the same two-locus haplotype as the *Foa* race 2, 19 non-pathogenic FOSC from celery, six non-pathogenic *F. commune* from celery, and 21 isolates from hosts other than celery or coriander. We used the 772 isolates in the GenBank *Fusarium* whole genome shotgun database to determine in silico if amplicons of interest were present*.* Because we had both DNA extracts and whole genome sequences from 12 of the 21 hosts other than celery or coriander, we were able to confirm that all of these in silico and empirical results concurred. Primers for elongation factor 1α (EF1/EF2) and/or rDNA (ITS1F/ITS4) were used as a positive control for amplification [3].

### Conidial and hyphal anastomosis experiments

Experiments testing for hyphal anastomosis between the *F. oxysporum* f. sp. *apii* and *coriandrii* isolates sequenced in this paper were conducted as described in Henry et al. [61]. Conidial anastomosis tube (CAT) formation was tested for *Foa* race 4 with either *Foci*3–2 or *Foa* race 2 using assays that were modified from those in Kurian et al. [33]. Five to 10-day old microconidia were harvested in water from PDA dishes, poured through a 40 μm mesh sieve, and washed with water. Conidia were then suspended in 10% potato dextrose broth (PDB) (1.25 X 10^6^ conidia/ml) and incubated for 3 h on a vertical rotary wheel in order to facilitate subsequent staining in wheat germ agglutinin (WGA). After conidia were again washed in water and poured through a 40 μm sieve, conidia were stained with WGA conjugated to either 20 μg Alexa Fluor 488 /ml water or to 24 μg Alexa Fluor 594/ml water (ThermoFisher Scientific, Waltham, MA). After 35 min on the rotary wheel, the conidia were precipitated by centrifugation and washed with 50 mM MgCl_2_ six times**.** After a PAP pen was used to delimit a one cm diam circle, a total of 9 X 10^4^ conidia in a 1:1 mixture of two strains of conidia, each with a different dye, in 70 μl of 0.25% PDB and 25 mM NaNO_3,_ pH 5.4, were deposited within the circle, and incubated in a humid chamber at 25 °C for 14 to 16 h. After wicking off moisture, cells were mounted in 75% glycerol.

CATs were evaluated with a 40X objective with differential interference contrast on a Leica DM500B epifluorescent microscope with GFP and rhodamine filters. Before scoring, the conidia of all CATs were carefully checked with both filters. Based on the fluorescent labels, CATs were classified as either hetero-CATs between two different strains or as one of the two types of homo-CATs with the same strain. Conidia that were either unstained or that were too clumped to be evaluated were not categorized. For each microscopic field of view in which there was at least one CAT that could be classified into one of the three categories, we recorded all score-able CATs and the total number of conidia with each of the two labels. Because there are twice the number of potential hetero-CATs as each type of homo-CAT in a 1:1 mixture of two strains, and because there were some deviations from the 1:1 mixture, CATs were quantified as a normalized fraction of the maximum number of potential CATs in the field of view. For each field of view, if A = number of conidia of one strain and B = number of conidia of the other strain, the number of hetero-CATs in that field was normalized by dividing by AB, which is the number of possible hetero-CATs. The number of homo-CATs was normalized by dividing by (A*(A-1))/2 and (B*(B-1))/2, respectively, i.e., the numbers of the possible homo-CATs.

Each experiment was conducted as three independent trials with statistically identical conclusions. Pooled trial results are shown; these represent a total of 102 fields of view with 741 CATs in the mixture of *FoaR4* and *Foci,* and 83 fields of view with 695 CATs with *Foa*R4 and *Foa*R2. Because the frequencies are not normally distributed, we used a Kruskal-Wallis rank sums test, and when *P* < 0.05, a Steel-Dwass nonparametric multiple comparison for all pairs in JMP Pro 14 (SAS Institute).

Images of CATs in Fig. 6 were captured with a 63X oil objective with differential interference contrast (DIC) on a Leica SP8 confocal microscope with GFP and rhodamine epifluorescent filters and z-stack capabilities. Overlay projections were made with Leica LAS X software. We note that some fuzziness in the images is the result of using DIC on spores on polystyrene; polystyrene is better than glass for inducing CATs, but is not ideal for DIC.

## Supplementary information

**Additional file 1.** Isolate collection

**Additional file 2 **Virulence of *F. oxysporum* f. sp. *apii* (*Foa*) races in three differential celery cultivars

**Additional file 3 **Virulence of *F. oxysporum* f. sp. *apii* and f. sp. *coriandrii* in celery and coriander

**Additional file 4 **Locations of the core genome in the *Foa* and *Foci* assemblies

**Additional file 5.** Percentage of the 3725 Benchmarking Universal Single-Copy Orthologs (BUSCO) in Sordariomycetes in the sequenced strains

**Additional file 6 **A cladogram of the mitochondrial genomes of the *Foa*, *Foci*, and 34 FOSC strains

**Additional file 7.** The numbers of genes and the sizes of the core and accessory genomes

**Additional file 8 **Classification of contigs in the *Foa* race 4 accessory genome as either lineage- or host-specific

**Additional file 9 **Classification of contigs in the *Foci3–2* accessory genome as either lineage- or host-specific

**Additional file 10 **Conserved synteny of BUSCOs in *Foa* race 4 and other *Foa*, *Foci* and a reference

**Additional file 11.** The number of homologs and their synteny in the accessory genomes

**Additional file 12 **Synteny between *Foci*GL306 and *Foa* race 4 in the conserved and accessory genomes

**Additional file 13 **Synteny between the two *Foci* strains in the conserved and accessory genomes.

**Additional file 14 ***Foa* race 4 putative accessory chromosomes: percentage of genes that have homologs in other strains

**Additional file 15 **Homologs of gene models from *Fol*4287 chromosome 14 in *Foa* race 4 and *Foci*GL306

**Additional file 16 **Up-expressed *in planta* RNA TagSeq-predicted effectors in *Foa* race 4: sequences, distribution, and mimp associations

**Additional file 17 **The percentage identity of the *Secreted In Xylem 1* (*SIX1*) orthologs in the *Foa, Foci,* and reference strain

**Additional file 18 **Test of PCR primers for *Foa* races 2 and 4 and *Foci* on a diversity of *Fusarium* spp.

**Additional file 19 **Whole genome-sequenced *Fusarium* spp. included in analyses in GenBank wgs

**Additional file 20 **Diagnostic PCR primers for *Foa* race 2 haplogroup, *Foa* race 4, and *Foci*

**Additional file 21 **Percentage of the genome with transposons and repeats in *Foa, Foci,* and the *Fol* reference

## Data Availability

The Whole Genome Shotgun projects have been deposited as assemblies at DDBJ/ENA/GenBankas JAAOOQ000000000 for *Foa* race 4, JAAOOP000000000 for *Foa* race 3, JAAOOO000000000 for *Foa* race 2, JAAOON000000000 for *Foci*3–2, and JAAOOM000000000 for *Foci*GL306 (Table 2). The version described in this paper is v1. The BioProject is PRJNA591157 with BioSamples SAMN13353346 and SAMN13353348-SAMN13353351. The PacBio reads are available from the Sequence Read Archive (SRA) under accessions: SRR10566868 - SRR10566878 (*Foci*) and SRR10533047-SRR10533072 (*Foa*). Illumina whole genome shotgun sequence reads are available from the SRA under accession numbers SRR10662418-SRR10662424. The TagSeq reads are available from the SRA under accessions SRR11347464 through SRR11347501. Much of the data generated or analysed during this study are included in this published article and its supplementary information files. Other data and materials are available from the corresponding author. Other DNA and predicted amino acid sequence data that was used in this research was obtained from and is available from GenBank at NCBI using the accession numbers shown in either the main text, Materials and Methods, or Additional files. Sequences for the phylogenomic study were obtained from https://www.ncbi.nlm.nih.gov/genome/browse/#!/eukaryotes/707/ and https://www.ncbi.nlm.nih.gov/genome/browse/#!/eukaryotes/Fusarium%20odoratissimum. Additional file 19 has a list of the whole genome sequenced *F. oxysporum* and *Fusarium spp*. in the GenBank wgs database as of September 30, 2020.

## Abbreviations

BUSCO: Benchmarking universal single-copy orthologs
CATs: Conidial anastomosis tubes
*Foa*: *Fusarium oxysporum* f. sp. apii
*Foci*: *Fusarium oxysporum* f. sp. coriandrii
*FOSC*: *Fusarium oxysporum* species complex
SIX1: Secreted in xylem 1
TE: Transposable elements
