# Supplementary material for: Genomic differences between the new Fusarium oxysporum f. sp. apii (Foa) race 4 on celery, the less virulent Foa races 2 and 3, and the avirulent on celery f. sp. coriandrii
Source: BMC Genomics. 2020 Oct 20;21:730. doi: 10.1186/s12864-020-07141-5 (PMC7576743; doi:10.1186/s12864-020-07141-5)
Supplement: Supplementary file 1 — Additional file 1. Isolate collection [file 12864_2020_7141_MOESM1_ESM.docx]

**Additional file 1.** Isolate collection

Isolates of *F. oxysporum* f. sp. *apii* from symptomatic celery were characterized by pathogenicity testing and two-locus sequence typing with *ef1* and *igs*; there were two isolates of *Foa* race 1*,* 22 isolates of *Foa* race 2, one isolate of *Foa* race 3-type from a culture collection, and 11 isolates of *Foa* race 4 [3]. Anecdotal evidence suggests that celery in “Field 0” for *Foa* race 4 were infected in ca. 2011. In 2013, we identified *Foa* race 4 in three fields in the Camarillo-Oxnard area in Ventura County in California. Between 2014 and 2019, as the pathogen spread to an increasingly larger area within Ventura County, we identified 46 *Foa* race 4 isolates from an additional 38 celery fields.

Between 2016 and 2018, we observed a syndrome in coriander and isolated *F. oxysporum* from symptomatic plants from seven fields in California; eight isolates were characterized by pathogenicity on coriander and celery, two-locus sequence typing (*ef1*/*igs*), and by PCR primers that are described later in this paper. *Foci*3-2 was selected for genome assembly (Table 1), as was *Foci*GL306, which was previously isolated in 2004 from neighboring Santa Barbara County [12].

 C. Response of celery after 56 days

| *Foa* race | Celery cultivar | Vascular discoloration-based rating from 0, asymptomatic to 5, dead | Aerial symptoms, % | Dead, % |
| --- | --- | --- | --- | --- |
| *Foa* race 2 | Golden Self Blanching | 1.8 d | 60 abc | 0 c |
|  | Tall Utah | 2.0 d | 50 bc | 0 c |
|  | Challenger | 0.4 e | 22 cd | 0 c |
| *Foa* race 3 | Golden Self Blanching | 2.9 c | 92 ab | 2 c |
|  | Tall Utah | 1.7 d | 18 cd | 0 c |
|  | Challenger | 1.4 d | 44 c | 0 c |
| *Foa* race 4 | Golden Self Blanching | 5.0 a | 100 a | 100 a |
|  | Tall Utah | 4.1 b | 90 ab | 62 b |
|  | Challenger | 4.4 ab | 100 a | 56 b |
| Uninfested control | Golden Self Blanching | 0.1 e | 0 d | 0 c |
|  | Tall Utah | 0.0 e | 0 d | 0 c |
|  | Challenger | 0.0 e | 0 d | 0 c |
