# Supplementary material for: Genomic differences between the new Fusarium oxysporum f. sp. apii (Foa) race 4 on celery, the less virulent Foa races 2 and 3, and the avirulent on celery f. sp. coriandrii
Source: BMC Genomics. 2020 Oct 20;21:730. doi: 10.1186/s12864-020-07141-5 (PMC7576743; doi:10.1186/s12864-020-07141-5)
Supplement: Supplementary file 2 — Additional file 2 Virulence of F. oxysporum f. sp. apii (Foa) races in three differential celery cultivars [file 12864_2020_7141_MOESM2_ESM.docx]

**Additional file 2.** Virulence of *F. oxysporum* f. sp. *apii* (*Foa*) races in three differential celery cultivars. In each of two trials, each with 25 replicate plants per treatment, cv. Golden Self Blanching (GSB), Tall Utah 52-70 R Improved (TU), and Challenger (CH) were transplanted in the greenhouse into either uninfested soil (U) or soil infested with *Foa* races 2 (R2), 3 (R3) or 4 (R4) and monitored weekly. A-B) Kaplan-Meier curves over 52 days. Treatments with 0% affected during the entire trial are not shown. A) The fraction of asymptomatic plants. Symptoms included yellowing (most frequently), stunting and/or wilting (least frequently). A log rank test (Chi-square=922, df=11, *P*<0.0001) and a Wicoxon (Chi-square=825, df=11, *P*<0.0001) indicate highly significant differences between cultivar-strain combinations. The combined days to symptoms was 40 with SEM=0.5. B) The fraction of survivors. A log rank test (Chi-square=664, df=11, *P*<0.0001) and a Wicoxon (Chi-square=646, df=11, *P*<0.0001) indicate highly significant differences between cultivar-strain combinations. The combined days to symptoms was 49 with SEM=0.3. C) ANOVA of symptoms at 56 days post-transplant. Within a column, for analyses with a significant F test, means followed by the same letter are not significantly different (α=0.05) by Tukey’s HSD. Vascular discoloration was scored on a scale of 0, asymptomatic; 1, some discoloration in the vasculature in lateral roots; 2, some discoloration in the vasculature of the main roots; 3, some discoloration in the vasculature of the crown; 4, extensive discoloration of the crown vasculature; and 5, plant dead. Percentage data were analyzed using trials as replicates.
