# Supplementary material for: Genomic differences between the new Fusarium oxysporum f. sp. apii (Foa) race 4 on celery, the less virulent Foa races 2 and 3, and the avirulent on celery f. sp. coriandrii
Source: BMC Genomics. 2020 Oct 20;21:730. doi: 10.1186/s12864-020-07141-5 (PMC7576743; doi:10.1186/s12864-020-07141-5)
Supplement: Supplementary file 3 — Additional file 3 Virulence of F. oxysporum f. sp. apii and f. sp. coriandrii in celery and coriander [file 12864_2020_7141_MOESM3_ESM.docx]

**Additional file 3.** Virulence of *F. oxysporum* f. sp. *apii* and f. sp. *coriandrii* in celery and coriander^a^.

| *F. oxysporum* strain | Celery cv. Tall Utah | Celery cv. Challenger | Coriander cv. Longstanding |
| --- | --- | --- | --- |
|  | Vascular discoloration-based rating from 0=asymptomatic to 5=dead^b^ | | |
| *Foa* race 2 | 3.8 b | 0.4 b | 4.4 a |
| *Foa* race 3 | 2.2 c | 0.4 b | 4.6 a |
| *Foa* race 4 | 4.7 a | 4.3 a | 4.7 a |
| *Foci*3-2 | 0.1 d | 0 b | 5.0 a |
| *Foci*GL306 | 0.1 d | 0 b | 5.0 a |
| Uninfested | 0 d | 0 b | 0 b |

^a^The pathogenicity and virulence of *F. oxysporum* f. sp. *apium* (*Foa*) races 2, 3, and 4 and two *F. oxysporum* f. sp. *coriandrii* (*Foci*) isolates in either the celery cultivars Tall Utah 52-70 R Improved or Challenger, and in the coriander cultivar Longstanding.

^b^These data were collected 56 days after either transplanting the celery or seeding the coriander. n=20. The scoring system is 0, asymptomatic; 1, some discoloration in the vasculature in lateral roots; 2, some discoloration in the vasculature of the main roots; 3, some discoloration in the vasculature of the crown; 4, extensive discoloration of the crown vasculature; and 5, plant dead. Within a column, for analyses with a significant F test, means followed by the same letter are not significantly (α=0.05) different by Tukey’s HSD.
