# Supplementary material for: Genomic differences between the new Fusarium oxysporum f. sp. apii (Foa) race 4 on celery, the less virulent Foa races 2 and 3, and the avirulent on celery f. sp. coriandrii
Source: BMC Genomics. 2020 Oct 20;21:730. doi: 10.1186/s12864-020-07141-5 (PMC7576743; doi:10.1186/s12864-020-07141-5)
Supplement: Supplementary file 4 — Additional file 4 Locations of the core genome in the Foa and Foci assemblies [file 12864_2020_7141_MOESM4_ESM.docx]

**Additional file 4.** Locations of the core genome in the *Foa* and *Foci* assemblies^a^

| *Fol* reference chromosome no. | *Fusarium oxysporum* strain | | | | | |
| --- | --- | --- | --- | --- | --- | --- |
|  | *Foa* race 4 | *Foa* race 3 | *Foa* race 2 | | *Foci*3-2 | *Foci*GL306 |
|  | Contig, Unitig (U) or Superscaffold (SS): bp_start_ – bp_end_^b^ | | | | | |
| 1 | SS12:72573-6058608 | 1:29926-5981626 | | 1:65662-6035196 | 1:162038-6137829 | 1:179442- 6153615 |
| 2 | SS11:396569-5061175 | 5:3057497-1489  5:3060960-3180414  5:4654206-3184969 | | 4:4719646-9761 | 6:4664059-739  6:4818032-4913602 | 6:4657892-90 |
| 4 | U7:5282662-61072 | 2:5266514-47724 | | 2:166795-5451515 | 3:453038-5645653 | 3:5276569-67101 |
| 5 | SS10:120094-5137752 | 3:87531-5082748 | | 3:4899109-40504 | 4:131063-5127215 | 4:5036752-41672 |
| 7 | U11:283068-421629  U11:739260-5088771 | 4:1-4282420  4:4729699-4602449 | | 5:4633510-56578 | 5:5050022-555610 | 5:77842-4576828 |
| 8 | U22:4026363-60183 | 7:1-3933658 | | 6:96225-4205359 | 8:145491-4270898 | 7:127323-4095931 |
| 9 | SS3:2854310-78881  SS12:6421088-6323837  SS12:6427888-6586853 | 8:32899-2806522  18:530595-153433  1:6260911-6523362 | | 7:3466954-138153 | 7:3554426-469659  22:514515-164628 | 8:3556918-468550;  26:89705-448060 |
|  | SS15:447349-516576 |  | |  |  |  |
|  | SS15:612952-820663 |  | |  |  |  |
| 10 | U19:145997-3107921 | 6:3857323-890704 | | 8:3076403-113319 | 2:6046770-3088076^c^ | 2:5956519-2988178 ^d^ |
| 11 | SS5:313551-2541971 | 9:302789-2524023 | | 10:464068-2622175  10:2698091-2642939  10:2809707-3043442 | 2:279367-2715249^c^ | 2:288703-2616039^d^ |
| 12 | U26:2613327-160962 | 10:2614471-305965 | | 11:210336-2393423 | 10:262376-2575949 | 14:293931-1188452  30:361203-10502  11:1364702-333626 |
| 13 | SS4:471270-2296753 | 11:1810057-25921 | | 12:389064-2232701 | 9:465874-2327815 | 9:2537220-674297 |

^a^The core genome is conserved amongst *F. oxysporum* strains. We identified conserved colinear blocks using progressiveMauve and the *F. oxysporum* f. sp. *lycopersici* (*Fol*) 4287 reference.

^b^SS denotes a Superscaffold that was constructed with Bionano. Sequences where the bp_start_ is greater than the bp_end_ are in reverse orientation relative to the *Fol* 4287 reference.

^c^A subsequent analysis identified a 198 bp juncture between Chromosomes 11 and 10 in *Foci*3-2 contig 2:2399802-2399999.

^d^ A subsequent analysis identified a 198 bp juncture between Chromosomes 11 and 10 in FociGL306 contig 2:2498982-2499179.
