# Supplementary material for: Genomic differences between the new Fusarium oxysporum f. sp. apii (Foa) race 4 on celery, the less virulent Foa races 2 and 3, and the avirulent on celery f. sp. coriandrii
Source: BMC Genomics. 2020 Oct 20;21:730. doi: 10.1186/s12864-020-07141-5 (PMC7576743; doi:10.1186/s12864-020-07141-5)
Supplement: Supplementary file 5 — Additional file 5. Percentage of the 3725 Benchmarking Universal Single-Copy Orthologs (BUSCO) in Sordariomycetes in the sequenced strains [file 12864_2020_7141_MOESM5_ESM.docx]

**Additional file 5.** Percentage of the 3,725 Benchmarking Universal Single-Copy Orthologs (BUSCO) in Sordariomycetes in the sequenced strains^a^**.**

| *Fusarium oxysporum* strain^b^ | Full-length genes present in assembly, % | Genes correctly assembled as a full-length, single copy, % | Genes complete but present as two or three homologs, % | Genes frag-mented, % | Genes missing, % |
| --- | --- | --- | --- | --- | --- |
| *Foa* race 4 | 98.8 | 98.1 | 0.8 | 0.8 | 0.4 |
| *Foa* race 3 | 98.8 | 98.1 | 0.6 | 0.9 | 0.4 |
| *Foa* race 2 | 98.7 | 97.6 | 1.1 | 0.9 | 0.4 |
| *Foci*3-2 | 98.7 | 98.0 | 0.8 | 0.9 | 0.4 |
| *Foci*GL306 | 98.8 | 97.9 | 0.9 | 0.9 | 0.4 |
| *Fol*4287 reference^c^ | 95.6 | 94.8 | 0.9 | 3.2 | 1.2 |

^a^BUSCO v2.0 [14]

^b^Strains of *Fusarium oxysporum* f. sp. *apii* (*Foa*)*,* f. sp. *coriandrii* (*Foci*) and the f. sp. *lycopersici* (*Fol*) reference are described in Table 1.

^c^Genbank [GCA_000149955.2 ASM14995v2](https://www.ncbi.nlm.nih.gov/assembly/475711)
