# Supplementary material for: Genomic differences between the new Fusarium oxysporum f. sp. apii (Foa) race 4 on celery, the less virulent Foa races 2 and 3, and the avirulent on celery f. sp. coriandrii
Source: BMC Genomics. 2020 Oct 20;21:730. doi: 10.1186/s12864-020-07141-5 (PMC7576743; doi:10.1186/s12864-020-07141-5)
Supplement: Supplementary file 6 — Additional file 6 A cladogram of the mitochondrial genomes of the Foa, Foci, and 34 FOSC strains [file 12864_2020_7141_MOESM6_ESM.docx]

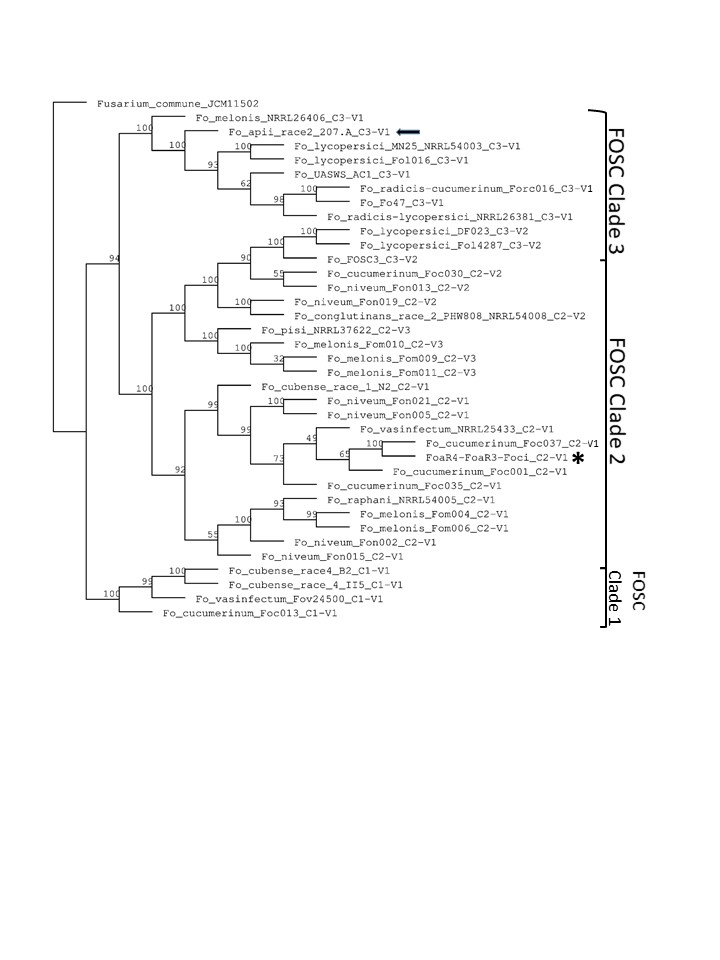


**Additional file 6.** A cladogram of the mitochondrial genomes of the *Foa*, *Foci*, and 34 FOSC strains. The 34 *Fusarium oxysporum* species complex (FOSC) references are from NCBI GenBank [15]. *F. commune* was used as an out-group. *F. oxysporum* f. sp*. apii* (*Foa*) races 3 and 4 and *F. oxysporum* f. sp. *coriandrii* strains *Foci* GL306 and *Foci*3-2 have an identical mitochondrial sequence and were collapsed into a single entry; they are shown with an asterisk. *Foa* race 2 is shown with an arrow. After the name of each strain, the value indicates the FOSC Clade and the V value indicates the Mitochondrial Variable Region Type. The tree was generated in Geneious (v.2020.0) with RaxML with the general time reversible evolutionary model and a search for the best-scoring maximum parsimony tree. Support for the tree is based on 1,000 bootstrap replicates.
