# Supplementary material for: Genomic differences between the new Fusarium oxysporum f. sp. apii (Foa) race 4 on celery, the less virulent Foa races 2 and 3, and the avirulent on celery f. sp. coriandrii
Source: BMC Genomics. 2020 Oct 20;21:730. doi: 10.1186/s12864-020-07141-5 (PMC7576743; doi:10.1186/s12864-020-07141-5)
Supplement: Supplementary file 7 — Additional file 7. The numbers of genes and the sizes of the core and accessory genomes [file 12864_2020_7141_MOESM7_ESM.docx]

**Additional file 7.** The numbers of genes and the sizes of the core and accessory genomes^a^

| Strain^b^ | Total no. of predicted coding nuclear genes | No. genes detected by 3’ RNA TagSeq^b^ | DNA in the core genome, Mbp | DNA in the accessory genome, Mbp |
| --- | --- | --- | --- | --- |
| *Foa* race 4 | 20,528 | 10,556 | 42.1 | 25.3 |
| *Foa* race 3 | 20,165 | 10,250 | 41.8 | 23.5 |
| *Foci*3-2 | 20,287 | NA^c^ | 42.5 | 23.0 |
| *Foci*GL306 | 20,208 | NA | 42.1 | 22.9 |
| *Foa* race 2 | 20,334 | 10,430 | 42.3 | 22.5 |
| *Fol* 4287^d^ | 20,925 | NA^c^ | 43.4 | 18.0 |

^a^The core genome was selected based on homology to the *F. oxysporum* f. sp*. lycopersici* 4287 core chromosomes 1, 2, 4, 5, and 7 through 13. The specific regions on the core contigs in our strains are indicated in Additional file 4.

^b^All detected genes had a minimum of a total of 10 or more “hits” in broth, or in the case of *Foa* race 4, in either broth and/or *in planta* libraries.

^c^NA, not applicable.

^d^*F. oxysporum* f. sp. *lycopersici* 4287 reference (Genbank [GCA_000149955.2 ASM14995v2](https://www.ncbi.nlm.nih.gov/assembly/475711))
