# Supplementary material for: Genomic differences between the new Fusarium oxysporum f. sp. apii (Foa) race 4 on celery, the less virulent Foa races 2 and 3, and the avirulent on celery f. sp. coriandrii
Source: BMC Genomics. 2020 Oct 20;21:730. doi: 10.1186/s12864-020-07141-5 (PMC7576743; doi:10.1186/s12864-020-07141-5)
Supplement: Supplementary file 9 — Additional file 9 Classification of contigs in the Foci3–2 accessory genome as either lineage- or host-specific [file 12864_2020_7141_MOESM9_ESM.docx]

**Additional file 9.** Classification of contigs in the *Foci*3-2 accessory genome as either lineage- or host-specific.

| *Fol* homo-log^a^ | *Foci*3-2 contig^b^ | *Foci*3-2 contig length, Mb | Strain^c^ | | | | | Differentiation between *Foci vs.* *Foa* races 3 and 4?^e^ |
| --- | --- | --- | --- | --- | --- | --- | --- | --- |
|  |  |  | *Fo* f. sp. *apii* race | | | *Foci* | |  |
|  |  |  | 2 | 4 | 3 | 3-2 | GL306 |  |
|  |  |  | Fraction of the *Foci* 3-2 assembly with highest-quality Illumina read coverage from the indicated strain^d^ | | | | |  |
| Chr1 | 1 | 6.7 | 0.92 | 0.94 | 0.98 | 1.00 | 1.00 | No, a core chromosome |
| Chr2 | 6 | 5.0 | 0.93 | 1.00 | 1.00 | 1.00 | 1.00 | No, a core chromosome |
| Chr4 | 3 | 5.7 | 0.87 | 0.97 | 0.99 | 1.00 | 0.94 | No, a core chromosome |
| Chr5 | 4 | 5.2 | 0.92 | 0.99 | 0.98 | 1.00 | 0.99 | No, a core chromosome |
| Chr7 | 5 | 5.2 | 0.84 | 1.00 | 1.00 | 1.00 | 1.00 | No, a core chromosome |
| Chr8 | 8 | 4.4 | 0.90 | 0.97 | 0.97 | 1.00 | 0.98 | No, a core chromosome |
| Chr9 | 7 | 4.9 | 0.79 | 0.98 | 0.98 | 1.00 | 1.00 | No, a core chromosome |
| Chr9 | 22 | 0.6 | 0.65 | 0.98 | 0.98 | 1.00 | 1.00 | No, a core chromosome |
| Chr10&11 | 2 | 6.2 | 0.86 | 1.00 | 1.00 | 1.00 | 1.00 | No, a core chromosome |
| Chr12 | 10 | 2.9 | 0.80 | 0.99 | 0.99 | 1.00 | 1.00 | No, a core chromosome |
| Chr13 | 9 | 3.0 | 0.74 | 0.99 | 0.99 | 1.00 | 1.00 | No, a core chromosome |
| Acc | 11 | 2.4 | 0.54 | 0.96 | 0.97 | 1.00 | 0.96 | No, LS |
| Acc | 12 | 1.7 | 0.35 | 0.30 | 0.29 | 1.00 | 1.00 | Yes, HS |
| Acc | 13 | 1.1 | 0.55 | 1.00 | 1.00 | 1.00 | 1.00 | No, LS |
| Acc | 14 | 1.1 | 0.56 | 1.00 | 1.00 | 1.00 | 1.00 | No, LS |
| Acc | 15^f^ | 1 | 0.54 | 0.30 | 0.30 | 1.00 | 1.00 | Yes, HS |
| Acc | 16 | 1 | 0.47 | 0.23 | 0.23 | 1.00 | 1.00 | Yes, HS |
| Acc | 17 | 0.8 | 0.61 | 0.61 | 0.61 | 0.61 | 0.61 | Unclear |
| Acc | 19 | 0.8 | 0.22 | 0.18 | 0.18 | 0.99 | 0.99 | Yes, HS |
| Acc | 20 | 0.6 | 0.41 | 0.45 | 0.44 | 1.00 | 1.00 | Yes, HS |
| Acc | 21 | 0.6 | 0.65 | 0.35 | 0.35 | 1.00 | 1.00 | Yes, HS |
| Acc | 23 | 0.6 | 0.60 | 1.00 | 1.00 | 1.00 | 1.00 | No, LS |
| Acc | 24 | 0.6 | 0.51 | 0.99 | 0.98 | 0.99 | 0.99 | No, LS |
| Acc | 25 | 0.4 | 0.51 | 1.00 | 0.96 | 1.00 | 1.00 | No, LS |
| Acc | 26 | 0.4 | 0.61 | 0.95 | 0.95 | 1.00 | 1.00 | No, LS |
| Acc | 27 | 0.3 | 0.52 | 0.99 | 0.99 | 0.99 | 0.99 | No, LS |
| Acc | 28 | 0.3 | 0.54 | 0.97 | 0.97 | 0.98 | 0.98 | No, LS |
| Acc | 29 | 0.2 | 0.47 | 1.00 | 1.00 | 1.00 | 1.00 | No, LS |
| Acc | 30 | 0.2 | 0.52 | 1.00 | 1.00 | 1.00 | 1.00 | No, LS |
| Acc | 31 | 0.2 | 0.75 | 0.99 | 0.99 | 1.00 | 0.99 | No, LS |
| Acc | 32 | 0.2 | 0.50 | 0.85 | 0.87 | 0.99 | 0.99 | Somewhat |

^a^Homologs of *F. oxysporum* f. sp. *lycopersici* (*Fol*) chromosomes were assigned with progressiveMauve. Chr, chromosome; Acc, Classified as part of the **acc**essory genome, i.e., without a homolog in the *Foa* and *Foci* strains.

^b^Only contigs with length greater than 150k bp were evaluated.

^c^*F. oxysporum* f. sp. *apii* race 2 is in *F. oxysporum* species complex (FOSC) Clade 3. *F. oxysporum* f. sp. *apii* races 3 and 4, and *F. oxysporum* f. sp. *coriandrii* (*Foci*) are in FOSC Clade 2.

^d^6.5 Gbp (≈100X) of quality-filtered (Phred score >20) Illumina reads were mapped onto the reference assembly and sequences were considered to be present in the query strain if greater than 10x coverage was observed. Here, the most saturated green indicates the most similar DNA with the *Foci*3-2 reference, yellow indicates moderately similar DNA and the most saturated red indicates the most dissimilar DNA.

^e”^No, a core chromosome” is expected. In the accessory (Acc) contigs, “No” indicates that the contig is “lineage-specific” (LS), i.e., shared between the *Foci* and *Foa* races 3 and 4. “Yes” indicates that the *Foci* versus *Foa* races 3 and 4 have different accessory contigs, i.e., these contigs are “host-specific” (HS).

^f^Has the *SIX1* homolog.
