# Supplementary material for: Genomic differences between the new Fusarium oxysporum f. sp. apii (Foa) race 4 on celery, the less virulent Foa races 2 and 3, and the avirulent on celery f. sp. coriandrii
Source: BMC Genomics. 2020 Oct 20;21:730. doi: 10.1186/s12864-020-07141-5 (PMC7576743; doi:10.1186/s12864-020-07141-5)
Supplement: Supplementary file 10 — Additional file 10 Conserved synteny of BUSCOs in Foa race 4 and other Foa, Foci and a reference [file 12864_2020_7141_MOESM10_ESM.docx]

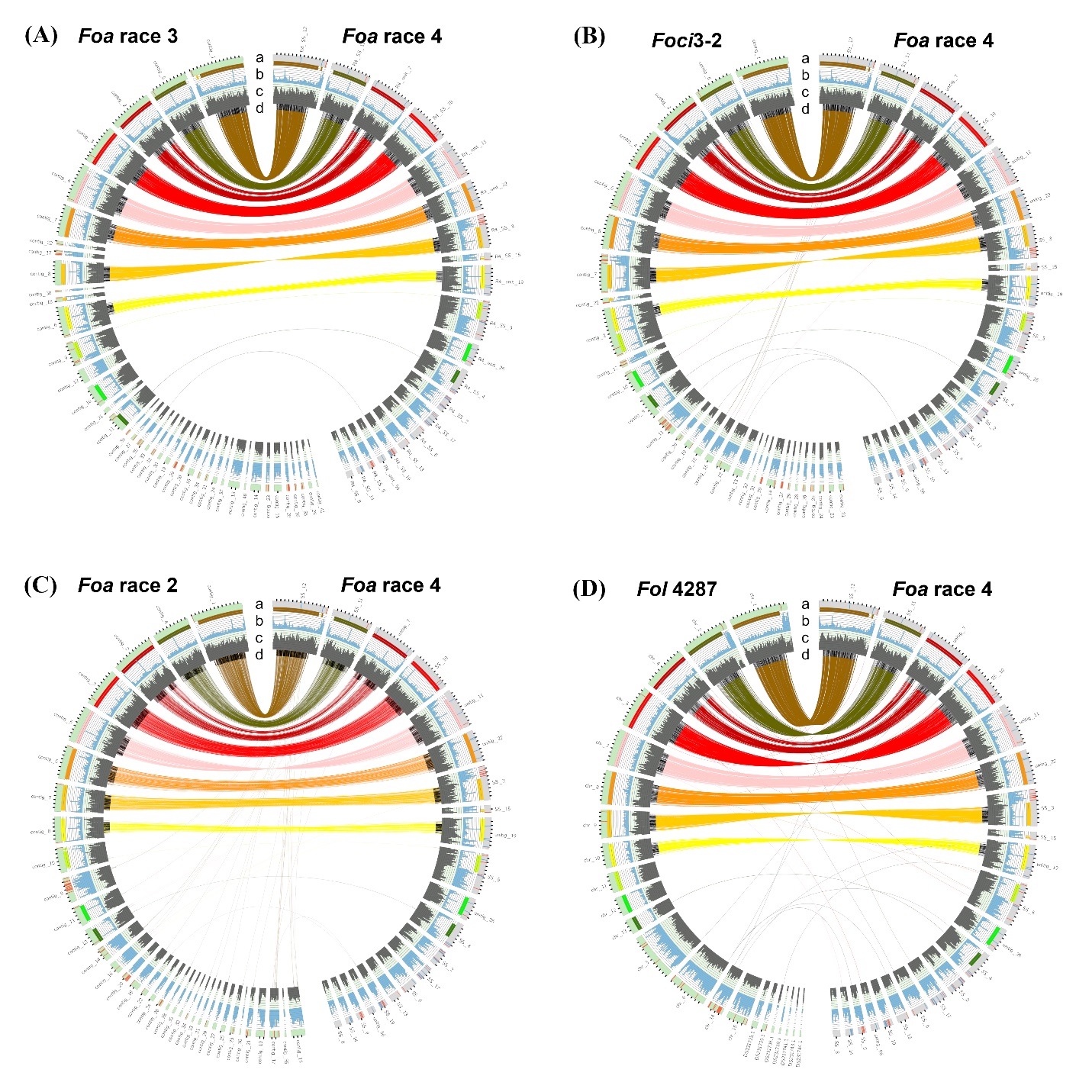


**Additional file 10.** Conserved synteny of BUSCOs in *Foa* race 4 and other *Foa*, *Foci* and a reference. An average of 3633 + 16 full-length Benchmarking Universal Single-Copy Orthologs [14] were analyzed per pair. *Fusarium oxysporum* f. sp. *apii (Foa)* race 4 is shown on the right side and, on the left side, the indicated strain. Contigs less than 150k bp are not shown. Tic marks on ring “a” are 500 kb apart. Within ring a, red lines indicate miniature impala transposable elements (mimps), and blue lines indicate all genes with significantly (adjusted *P*<0.05) increased expression *in planta* in celery crowns that were infected with *Foa* race 4 compared to *Foa* race 4 grown *in vitro*. In ring b, the solid colors within the upper portion denote a region with homology to one of the *Fol* core chromosomes. Blue shows the density of repetitive elements with a full scale of 120 per 100 kb increment. In ring c, dark grey shows the density of gene models with a full scale of 50 per 100 kb increment. In ring d, the grey lines show predicted BUSCO genes, and links between BUSCO genes indicate the position of the homolog in the other strain. Links connecting BUSCO homologs in core chromosomes are color coded, and BUSCO genes in accessory contigs are connected by black lines. The plots illustrate the high degree of synteny in all four pairs of strains in the core chromosomes, even across FOSC Clade 2 (*Foa* race 4) and FOSC Clade 3 (the *Fol* 4287 reference and *Foa* race 2). Note that the BUSCO genes are concentrated in orthologs of only core chromosomes 1,2,4,5, and 7-10 and not in core chromosomes 11-13.
