# Supplementary material for: Genomic differences between the new Fusarium oxysporum f. sp. apii (Foa) race 4 on celery, the less virulent Foa races 2 and 3, and the avirulent on celery f. sp. coriandrii
Source: BMC Genomics. 2020 Oct 20;21:730. doi: 10.1186/s12864-020-07141-5 (PMC7576743; doi:10.1186/s12864-020-07141-5)
Supplement: Supplementary file 11 — Additional file 11. The number of homologs and their synteny in the accessory genomes [file 12864_2020_7141_MOESM11_ESM.docx]

Additional file 11: The number of homologs and their synteny in the accessory genomes^a^

| Reference strain | Target  Strain | Homologous gene models in the accessory genome, % of the total in the reference genome^b^ | No. homologous genes in the accessory genomes^b^ | No. syntenic genes in the accessory genome of the target strain^c^ | Syntenic genes in the common accessory genome, % | |
| --- | --- | --- | --- | --- | --- | --- |
| *Foa* race 4 | *Foa* race 3 | 64 | 3949 | 3546 | 90 |  |
| *Foa* race 4 | *Foci*3-2 | 50 | 3011 | 2523 | 84 |  |
| *Foa* race 4 | *Foci*Gl306 | 46 | 2854 | 2342 | 82 |  |
| *Foa* race 4 | *Foa* race 2 | 15 | 943 | 390 | 41 |  |
| *Foa* race 4 | *Fol*4287^d^ | 14 | 853 | 302 | 35 |  |
| *Foci*GL306 | *Foci*3-2 | 75 | 4317 | 4079 | 94 |  |
| *Foci*GL306 | *Fol*4287 | 15 | 853 | 274 | 32 |  |

^a^The accessory genomes include all gene models that are not in the *F. oxysporum* core genome (Additional file 4) and are on contigs larger than 150 kbp.

^b^Homologous pairs of gene models have > 80% identity over > 80% of the predicted nucleotide sequence. When a single gene model could have multiple partners, only the “reciprocal best BLAST” (RBBH) is selected. The lines in the centers of Fig. 4, and Additional files 12 and 13B-D connect the homologous pairs.

^c^After the genes in each reference contig were sorted by locus, a gene in the target strain of a homologous pair was considered syntenic if belonged to an ascending or a decending run of at least three target genes within a single target contig.

^d^*F. oxysporum* f. sp. *lycopersici* 4287 reference (Genbank [GCA_000149955.2 ASM14995v2](https://www.ncbi.nlm.nih.gov/assembly/475711)).
