# Supplementary material for: Genomic differences between the new Fusarium oxysporum f. sp. apii (Foa) race 4 on celery, the less virulent Foa races 2 and 3, and the avirulent on celery f. sp. coriandrii
Source: BMC Genomics. 2020 Oct 20;21:730. doi: 10.1186/s12864-020-07141-5 (PMC7576743; doi:10.1186/s12864-020-07141-5)
Supplement: Supplementary file 12 — Additional file 12 Synteny between FociGL306 and Foa race 4 in the conserved and accessory genomes [file 12864_2020_7141_MOESM12_ESM.docx]

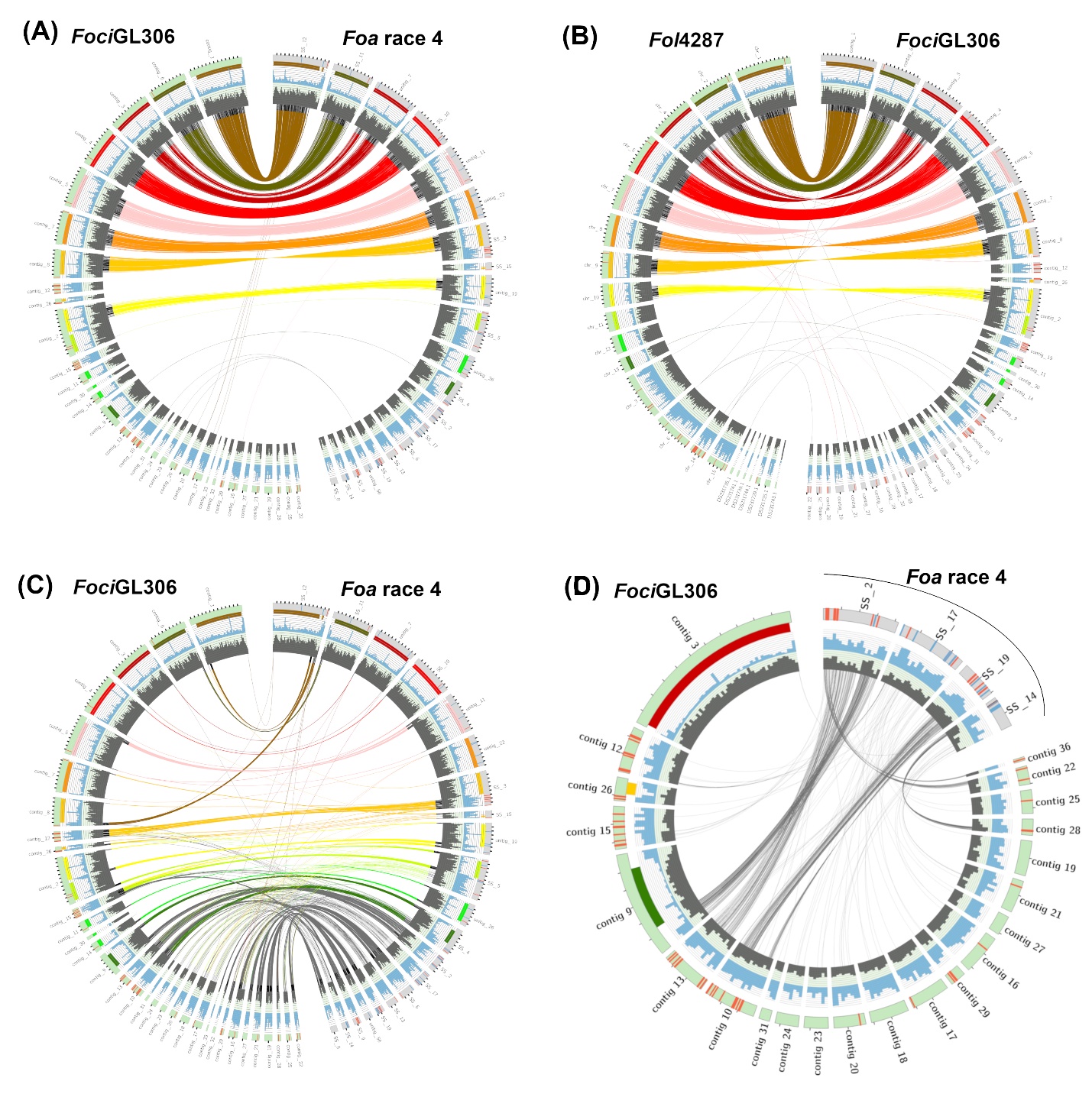
 **Additional file 12.** Synteny between *Foci*GL306 and *Foa* race 4 in the conserved and accessory genomes. A, C, and D) *Foa* race 4 contigs are on the right and *F. oxysporum* f. sp. *coriandrii* *Foci*GL306 contigs are on the left. B) *Foci*GL306 contigs are on the right and contigs of the *Fol*4287 reference are on the left. A, B) “reciprocal best BLAST hits” (RBBH) of full-length, single copy BUSCO Sordariomycete genes. C) RBBH of genes in the non-core/accessory regions of the *F. oxysporum* genome, and D) Best BLAST hits (BBH) of genes in the four accessory contigs of *Foa* race 4 that have the most up-expressed genes *in planta* compared to *in vitro*. Within ring a, red lines indicate miniature impala inverted-repeat transposable elements (mimps). In ring b, the solid colors within the upper portion of ring b denote a region with homology to one of the *Fol* core chromosomes. Blue shows the density of repetitive elements with a full scale of 120 per 100 kb increment. In ring c, dark grey shows the density of gene models with a full scale of 50 per 100 kb increment. In ring d, the grey lines show genes that have a BBH with > 80% identity over > 80% of the predicted nucleotide sequence. In the center, lines connect the BBH; genes connected by black lines are in accessory contigs and genes connected with other colors denote the particular core chromosome. Genes that have a grey line in ring d but no connecting line have a BBH in a contig that is not shown in that figure. The plots indicate that the synteny of *Foci*GL306 to both *Foa* race 4 and *Fol*4287 are similar to those observed with *Foci*3-2.
