# Supplementary material for: Genomic differences between the new Fusarium oxysporum f. sp. apii (Foa) race 4 on celery, the less virulent Foa races 2 and 3, and the avirulent on celery f. sp. coriandrii
Source: BMC Genomics. 2020 Oct 20;21:730. doi: 10.1186/s12864-020-07141-5 (PMC7576743; doi:10.1186/s12864-020-07141-5)
Supplement: Supplementary file 13 — Additional file 13 Synteny between the two Foci strains in the conserved and accessory genomes. [file 12864_2020_7141_MOESM13_ESM.docx]

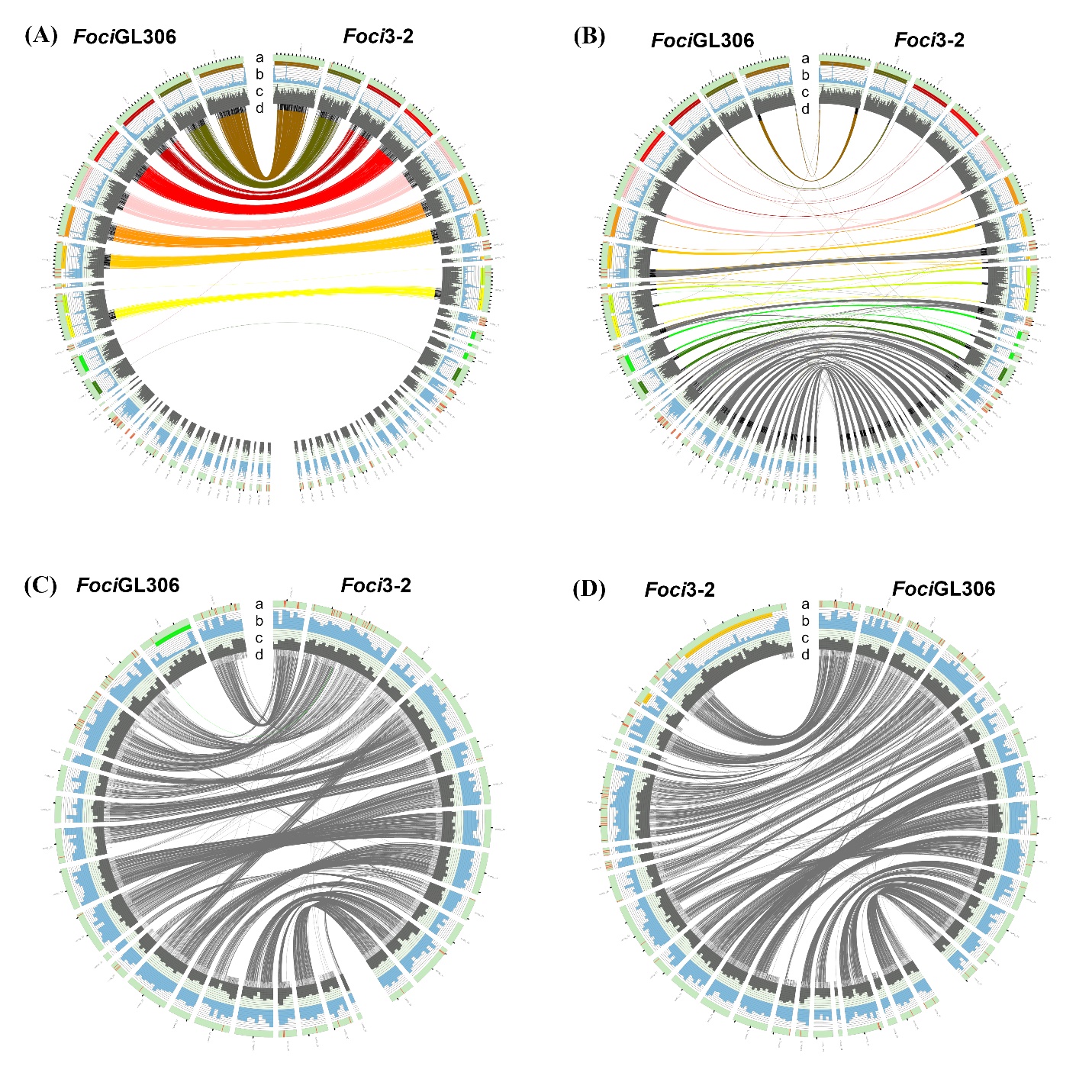


**Additional file 13.** Synteny between the two *Foci* strains in the conserved and accessory genomes. Circos plot comparisons of homologs of A-C) *F. oxysporum* f. sp. *coriandrii* (*Foci*3-2) on the right side and, on the left side to *Foci*GL306. A-B) Contigs less than 150 kb are not shown. A*)* 3,650 and 3,645 single copy, full-length, single-copy BUSCO Sordariomycete genes in *Foci*3-2 and *Foci*GL306, respectively, B) “Reciprocal best BLAST hits” (RBBH) with > 80% identity over > 80% of the nucleotide sequence in non-core regions of the *F. oxysporum* genome. C-D) Contigs less than 100k bp are not shown. C) RBBH of genes in the 12 accessory contigs of *Foci*3-2. D) RBBH of genes in the 14 accessory contigs of *Foci*GL306 are shown on the right with *Foc*3-2 on the left. Within ring a, red lines indicate miniature impala transposable elements (mimps). In ring b, the solid colors within the upper portion of ring b denote a region with homology to one of the *Fol* core chromosomes. Blue shows the density of repetitive elements with a full scale of 120 per 100 kb increment. In ring c, dark grey shows the density of gene models with a full scale of 50 per 100 kb increment. In ring d, the grey lines indicate genes that have a RBBH. In the center, lines connect the RBBH; black lines connect genes in accessory contigs and lines in other colors denote the particular core chromosome. Genes that have a grey line in ring d but no connecting line have a RBBH in a contig that is not shown in that figure. The plots show that *Foci*3-2 and *Foci*GL306 are highly syntenic in both the conserved and accessory contigs. Nevertheless, there are some rearrangements between the genomes, most notably in the accessory contigs, as shown in C and D.
