# Supplementary material for: Genomic differences between the new Fusarium oxysporum f. sp. apii (Foa) race 4 on celery, the less virulent Foa races 2 and 3, and the avirulent on celery f. sp. coriandrii
Source: BMC Genomics. 2020 Oct 20;21:730. doi: 10.1186/s12864-020-07141-5 (PMC7576743; doi:10.1186/s12864-020-07141-5)
Supplement: Supplementary file 14 — Additional file 14 Foa race 4 putative accessory chromosomes: percentage of genes that have homologs in other strains [file 12864_2020_7141_MOESM14_ESM.docx]

**Additional file 14.** *Foa* race 4 putative accessory chromosomes: percentage of genes that have homologs in other strains

| Strain | Selected *Foa* race 4 accessory superscaffolds (SS)^a^ | | | |
| --- | --- | --- | --- | --- |
|  | SS2 | SS17 | SS19 | SS14 |
|  | Percent of predicted genes with a reciprocal Best Blast Hit^b^ | | | |
| *Foa* race 3 | 63 | 59 | 73 | 61 |
| *Foci*3-2 | 70 | 8 | 58 | 25 |
| *Foci*GL306 | 56 | 9 | 41 | 25 |
| *Foa* race2 | 20 | 7 | 6 | 12 |
| *Fol*4287 reference | 17 | 7 | 5 | 11 |

^a^The four superscaffolds that were selected for the Circos plots in Fig. 5 are part of the accessory genome and have a minimum length of 800 kbp. SS2 (2.1 Mbp) and SS19 (1.0 Mbp) are lineage-specific contigs (see Additional File 8). SS17 (1.9 Mbp) and SS14 (0.9 Mbp) are host-specific contigs. Of the 23 highly-expressed putative effectors in Table 4, SS17 has eight, SS14 has five and SS19 has two.

^b^To be considered a reciprocal Best Blast Hit, gene X must have a BLAST hit with gene Y that has the lowest E-value and > 80% identity over > 80% of the predicted nucleotide sequence, and gene Y must also have a BLAST hit with gene X that has the lowest E-value and >80% identity over 80% of the sequence.
