# Supplementary material for: Genomic differences between the new Fusarium oxysporum f. sp. apii (Foa) race 4 on celery, the less virulent Foa races 2 and 3, and the avirulent on celery f. sp. coriandrii
Source: BMC Genomics. 2020 Oct 20;21:730. doi: 10.1186/s12864-020-07141-5 (PMC7576743; doi:10.1186/s12864-020-07141-5)
Supplement: Supplementary file 15 — Additional file 15 Homologs of gene models from Fol4287 chromosome 14 in Foa race 4 and FociGL306 [file 12864_2020_7141_MOESM15_ESM.docx]

**
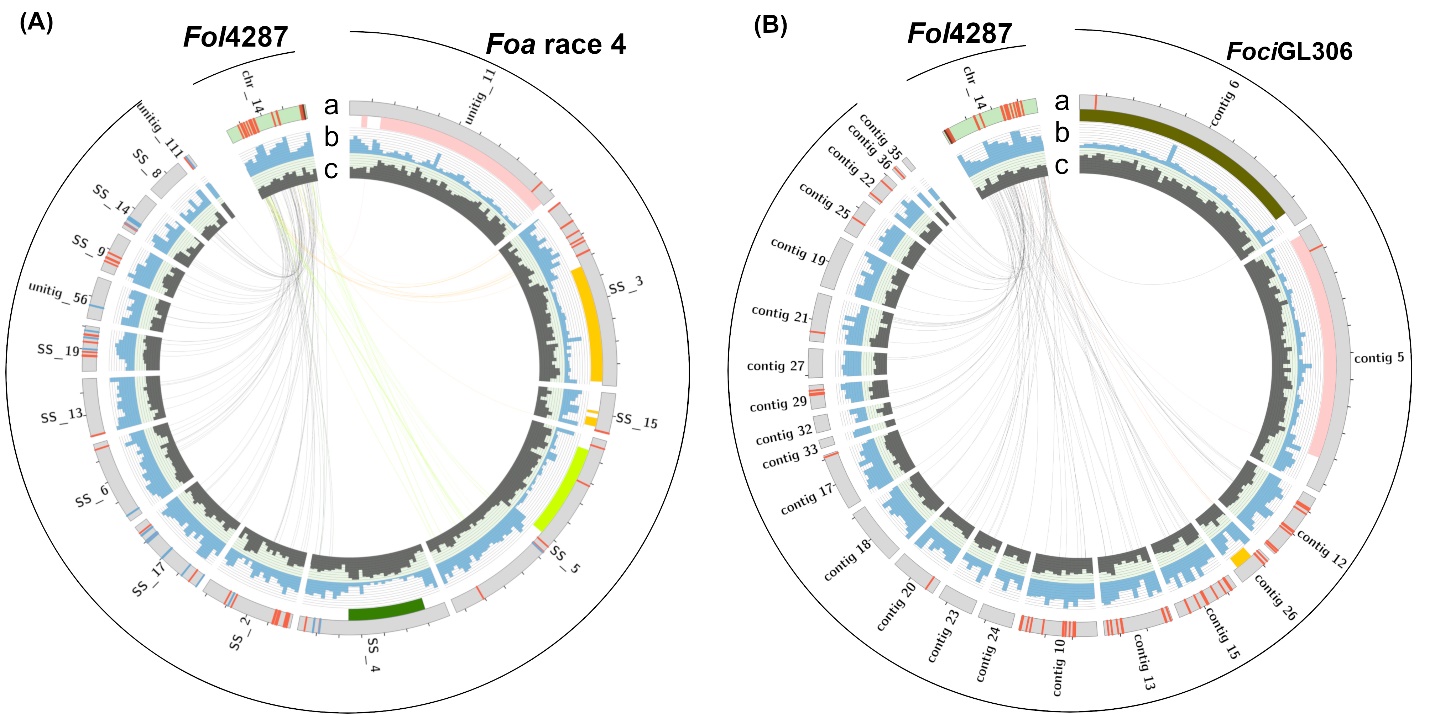
**

**Additional file 15.** Homologs of gene models from *Fol*4287 chromosome 14 in *Foa* race 4 and *Foci*GL306. A Circos plot of homologs of genes in the pathogenicity chromosome 14, which is in the accessory genome of *F. oxysporum* f. sp. *lycopersici* (*Fol*) 4287, in contigs > 150 kbp of A) *Fusarium oxysporum* f. sp. *apii* (*Foa*) race 4 and B) *Foci*GL306. Tic marks on ring a are 500 kb. Within ring a, red lines indicate miniature impala transposable elements (mimps), and blue lines indicate all genes with significantly (*P*<0.05) increased expression *in planta* in celery crowns that are infected with *Foa* race 4 than in *Foa* race 4 grown *in vitro*. Withn ring b, the solid colors on the upper portion denote a region with homology to one of the *Fol* core chromosomes (shown in Additional files 10D and 12B); the blue represents the density of repetitive elements with a full scale of 120 per 100 kb increment. In ring c, dark grey shows the density of gene models with a full scale of 50 per 100 kb increment. In ring d, the grey lines show genes that have a “best BLAST hit” (BBH) with a > 80% identity over > 80% of the predicted nucleotide sequence. In the center, lines connect the BBHs. Black lines connect BBH genes in both accessory genomes; colored lines denote a color-coded core chromosome. *Fol*4287 chromosome 14 has 414 genes; based on the BBH criterion, *Foa* race 4 and *Foci*GL306 have homologs of 28 and 23% of these genes, respectively.
