# Supplementary material for: Genomic differences between the new Fusarium oxysporum f. sp. apii (Foa) race 4 on celery, the less virulent Foa races 2 and 3, and the avirulent on celery f. sp. coriandrii
Source: BMC Genomics. 2020 Oct 20;21:730. doi: 10.1186/s12864-020-07141-5 (PMC7576743; doi:10.1186/s12864-020-07141-5)
Supplement: Supplementary file 16 — Additional file 16 Up-expressed in planta RNA TagSeq-predicted effectors in Foa race 4: sequences, distribution, and mimp associations [file 12864_2020_7141_MOESM16_ESM.docx]

**Additional file 16**. Up-expressed *in planta* RNA TagSeq-predicted effectors in *Foa* race 4: sequences, distribution, and mimp associations^a^

| Gene | Predicted amino acid sequence | DNA identity in *Foa &Foci* strains^b^ | Distance of mimp(s) from ORF start, bp^c^ |
| --- | --- | --- | --- |
| NS.09678 | MVKHIQLPGLSLAATAMLAARGADSKVVCVNPNKEVVADTKCENVKAPGNFYMVRSMSDSLAPGSLVSADADMNDAFYPIDRANALFPPDMTSGGFGKRDCGGSGANGGNGSGGRGGTVIVGGGYHGG | 100% in all FOSC Clade 2; 95% identity in *Foa* race 2; 2^nd^ copy in *Foa* races 3 & 4 | 480 |
| NS.05815 | MAPYSMVLLGTLSILGFSAYAQEAAVPEPQVFFNLTYAEHLEKVAASSGSVPDNSDLPWEDTIPWNGTDDGVQTETGSSLSRRGRIFNLGKREPVGGETRNDAVTNDMLQALHEMCVERFGTGHRATNGRCRGRHKQVECGHPDVAGRVGQVGKSCAEGQECTTFQAVNFRNRRATFPVCGPRIEVKERHDIGRHTEWEGTWYPESPKSPGTYDSFAQMAGSLNGYFDFNGVYSSGEGMSSRGSGHSWSCIACPGGKLTITSTLRATWAIGYTSP | 100% in *Foa* races 3 and 4; 100% within the *Foci;* two copies (one is NS.05829) in all *Foa* | NA^d^ |
| PGN.06282 | MHVKNAIVLLFAASAISMPLESVHNSGHGAIGLERRDISHSETTEEKRELIG | Assembly indicates that this sequence is only present as two copies in *Foa* race 4, but PCR and raw reads indicate that there it is present in *Foa* race 3; absent in *Foa* race 2 and *Foci* | 979^e^ |
| NS.06742 | MIARTSTVLSLALTALSLGAYAAPGPAPNNDVSQALRSPLEARASLCCAVATNNRYIQTVCQYMYESCRGWNKCLKGLPNDSDWCHYCVVVHPEDKACLTKTWPPVGHAPISKRGIDDATILPPEDSAKDNAETAAPVSRRSLDMAPGSDDSSNAAPDLEDFRDTTSKHLDKRAWSDQNIHTRNLHNEAQSYRRAIGLVTIRIIISASNVMTWSVQNSGATDVAFHVIDRVSGWKVERTIHAGQTDGGAPGSQVLAQGGDTFTVGIQR | 100% in all FOSC Clade 2; three copies in *Foa* race 2 | NA |
| NS.06525 | MVKHIQLPGLSLAATAILAARGADSKVVCVNPNKEVVADTKCEDVKAPGNFYMVRSMSDNLALGSLVSADAEMNDAFYPIDRANALFPPDMTSGGFGKRDCGGSGGNGGNGSGGRGGTVVIVGGGYGG | 100% in *Foa* races 3 and 4; 100% within the *Foci;* two copies in *Foa* races 3 and 4; one copy in *Foci* and *Foa* race 2 | 764 and 1100 |
| PGN.05952 | MLLNTIWKVVAFSPAAFAAIRARESHGWCFCARKGIEGNDGLDWGLTSVVCGDFPVSVSFDQKDHKCYTTSGESRIDGDTWEAHCKSYASDGYEFEGVKYKWNARDIKGKCS | 100% in *Foa* races 3 and 4; one copy in *Foa* race 2; absent in *Foci* | NA |
| PGN.20363 | MRAYVSLTVFSLFLRVSAWNKCYCTGSDQAVADTTANTCCTDGDGVSFPHTKGDVKGRWDSSNKICVFSGQVLSQNSENDATAAFAACCRPTGGSTIWGGACS | 100% in all FOSC Clade 2; absent in *Foa* race 2 | NA |
| PGN.05922 | MRFATFGLLVVLPTFSLAKDCGIFYDWSGTSHLDVWRDLKAVSMCSDIGGDIPNDELALKNGNGVHRCAVCRNARGGTKDYTRTITQQNDKIIYSVRCGWFGKGKCSA | 100% in all FOSC Clade 2 with a 2^nd^ copy in *Foa* race 3; absent in *Foa* race 2 | NA |
| PGN.15680 | MHPFTALSATFVAIMSPALINAQSAVSTAVAPAPTGMGCICMAPTSSGKDEAMYDRTWRCCRQEQGHMRSTNDFWGRGSFYCNFEKGINPAEWDGNCCRRVFGDGTYGFCNKAV | 100% in each of 3 copies of *Foa* races 3 and 4; one copy in *Foa* race 2; absent in *Foci* | NA |
| PGN.06635 | MSASMRISTFGIFAVLPILGLAKDCAVYYDYVGDVVKVGSSMGFAETDPDLNEKRRATASKICTHDIGGHINSPGGWAPEAGITNGHNPVNRCTICRGARGGTRDYDKAADGITYSIRCGYFGPYLCSAK | 100% in *Foa* races 3 and 4; 100% in the *Foci* and a 2^nd^ copy in *Foa* races 3 and 4. Absent in *Foa* race 2. | 383 |
| NS.06362 | MKFITLTLLFGTGILGVKGDWDLYRDVQCNYKKSSVPLWHNCFVNGKEPYTDNDCGLACARAGMNEGNKFVHGSNYNGGQSCGLICYFK | 100% in *Foa* races 3 and 4; one copy in *Foci*;*.*  two copies with 79.5 and 79.9% identity over 99% in Foa race 2 | 183 |
| PGN.09917 | MKFSSLLLGVPFLVRIHAAECPRASVSGNAITGFRYFNDCTTWTWRSRDKGTTVTLSPDCILRQAWPNPQNVWAVCIRLEGGEYQCFQTGANGAECSVPSPWCSTTAKIANMWGW | 100% in *Foa* races 3 and 4; 100% in the *Foci*; *Foa* races 3 and 4 have an identical 2^nd^ copy, and *Foa* race 3 has a 3^rd^ copy. Foa race 2 has one copy | NA |
| NS.05829 | MAPYSMVLLGALSILGSAAYAQEAAVQEPQIFFNLTYTEYLDKVAAASGRSPPENSDLPWDDTMSSLPLNETDDDVQPEIASSLSRRGRIFNLGKREPVGGETRNDAVTNDMLQALHDLCVERHGTGWRATGGRCDGRTRRINCGNPGIGQSIRRVSSACPQNQECTTFEAINFRRSRTNFPVCGPRIEVAEKHDIGSHTEWEGTWYPESPKSPGTYDSFAQMAGSLNGYFDFNGVYSNGNGMSSRGSGHSWSCIACPGGKLTITSTYRSTWAVGYTSPH | 100% in *Foa* races 3 and 4; these strains have an identical 2^nd^ copy; *Foa* race 2 has two copies. *Foci* have one copy with 79.5% identity. | NA |
| PGN.07042 | MSASMRISTFGIFAVLPILGLAKDCAVYYDFVGDVVKVGSSMGFAETDPGLNEKRRATASKICTHDIGGHVNSPGGWAPEAGITNGNNPVNRCTICRGARGGTRDYDKAADGITYSIRCGYFGPYLCSAK | 100% in all FOSC Clade 2; *Foa* races 3 and 4 have an identical 2^nd^ copy; absent in *Foa* race 2 | NA |
| PGN.06691 | MKFSSLLLGAPFLVRIHAAECPRASVSGNAITGFKYFNYCTTWTWRSRDKGTTVTLSPDCILRQAWPNPQNVGAVCVRLEGGGDQCFQTGANGAECSVPSPWCSTTAKIANMWGW | 100% in *Foa* races 3 and 4; All FOSC Clade 2 have a 2^nd^, identical copy; *Foa* race 3 has a 3^rd^ copy; *Foa* race 2 has one copy | 270 and 605 |
| NS.01422 | MFGSISTYFVTLLAAASTVANAAATSKNPVYTGLGTRYGDSDGCTEEDCWQKGACSFVDYKLPAGIDGTTCVSEDIWKDGANCGGCIQVSYKGKSLKIMVTNKTGGDKNHLDMTPATWSKLTSGMTGGGVDGIKWKWIACPLKSPLQVHMHGGASKYWFAATIENITHRVKAVEVSSDSGKTWKATTLKDPNMWILKGTLPNDTAWVRVTSVNNKKVIVKNVALKSGVVTKGTSNF | 100% in all FOSC Clade 2; Foa race 2 has one copy | NA |
| NS.09643 | MLYPRFQSATVAVIAAVLTPLVLGAATPTPQPSIQWETTTKDILLSEIGPFDLESQLTSSGSTGTSSTMNKRSSYSAGVCFAIPFKPQTGGAWGFKQAWCDRTGTDVNTFRVDCFGGRNYIETLPNRKGACGKGQWCVDYHGRNSKGDAADDVLCVNRKDIHTWVANTQTRPVEDKVTCSSGWRNDYKQSAKATFEVDVMDSAGINRIAPENVYYILNQKRIGVSRSNDAEVGSGYITIPPGGAIQACVTAKVAQNQILNMLGAITSFKLL | 100% in all FOSC Clade 2; Foa races 3 and 4 have an identical 2^nd^ copy; the *Foci* have an identical 2^nd^ & 3^rd^ copy; *Foa* race 2 has 17 copies | NA |
| NS.16793 | MKLLTLISAFATATLVSADQRAQLSAPDGSVHHLSARDGTCPRPMCKTPASQGPNDPPACGDSYAACKFDQFPCDEHFSPKVTDTHHCYCILANKKAMDAYCQERGFKSGTNPWKYYYAVECHGAVSNQVCNKDCHDQGRGNGRIDKAHPNGACACDKPNPPYDTCKA | 100% in all FOSC Clade 2; *Foa* race 2 has two copies | NA |
| NS.06528 | MEHLGIATNSSTPNSLYGQQILRFRCDTRQHRLLYKMHITKFVVAVALPLLAAANEHVGCKCNTGDATCLEVACNSYSAAGVFFNKPKGHENSVFSQTQDGKCYAVYNNGQEYLTFKGLGGKEWLQQCEAHCGGGSTC | 100% in *Foa* races 3 and 4; all FOSC Clade 2 have three other copies except *Foci*GL306, which has four; *Foa* race 2 has two copies | NA |
| PGN.06376 | MHPFTALSATFVAIMSPALINAQSAVSTAVAPAPTGMGCICMAPTSSGKDEAMYDRTSRCCRQEQGHMRSTNDFWGRGSFYCNFEKGINPAEWDGNCCRRVFGDGTYGFCNKAV | 100% in *Foa* races 3 and 4; 100% in two other copies in *Foa* races 3 and 4; *Foa* race 2 has one copy; absent in *Foci* | 215 |
| NS.15045 | MKLSAVTLLTLATGILAAPVAEANYDVSYSSYEAPKAPKPHYEKPKPKPHHEKPKHEYPAPHHEKPKPKPHPKPHYEKPKKPEYQAPKPHYEKPKPKPHPKPAYEAPKPAPKPHKPEYTKPKAPKPHKPEYTAPKPVKPKYTKPEAPKPHKPEYTKPKAPKPHPKPAYEAPKPAPKPHKPEYTKPKAPKPHKPEYTAPKPHHEKPKPHPKPTYQAPKPAPKPHKPEYTKPKTEKPKPAPKPEYKAPTYQAPHY | 100% in all FOSC Clade 2; one copy in *Foa* race 2 | NA |
| PGN.05959 | MKITSIAILAFLSATATAQTGSKYLSYCKGTSQDLGQSLCKKKGGTWGPRTDAPAEYRSRSGYYCLGAGWWGTDPCPAEYGKGFQVVNFNT | 100% in *Foa* races 3 and 4; absent in *Foa* race 2 and *Foci* | NA |
| PGN.06650 | MHPFTALSATFVAIMSPALINAQSAVSTAVPPAPTGMGCICMAPTSSGKDEAMYDRTSRCCRQEQGHMRSTNDFWGRGGFYCNFEKGINPAEWDGNCCRRVFGDGTYGFCNKAV | 100% in *Foa* races 3 and 4; 100% in two other copies in *Foa* races 3 and 4; one copy in *Foa* race 2; absent in *Foci* | NA |

^a^Based on 3’RNA QuantSeq read mapping, all genes 1) had significantly (adjusted *P*<0.05) higher expression *in planta* in celery crowns than *in vitro* and 2) accounted for more than 0.1% of the total fungal reads *in planta,* i.e., were relatively highly expressed *in planta*. Based on DNA sequence analysis, all predicted proteins were 1) secreted into the plant, 2) had a predicted mol wt of < 35 kDa, and 3) were neither “house-keeping” nor nutrition-associated genes.

^b^The DNA sequence of predicted effector genes of *Foa* race 4 were used as a reference for the other strains: *Foa* races 2 and 3, *Foci*3-2, and *Foci*GL306. Identical sequences are indicated as 100%. Sequences with DNA identities >80<100% over the full length are called copies, unless specifically identified as > 70% identity. Absence is defined as an e>0.1. *Foa* races 3 and 4 and the two *Foci* are in FOSC Clade 2.

^c^Miniature impala (*mimp*) transposable elements were identified with TIRmite (version 1.1.3). Using the sequence “..CAGTGGG..GCAA[TA]AA,” hidden Markov models were used to identify terminal inverted repeats using a four-time reiterative process (<https://github.com/SamuelBrinker/Repertoire_v6>). *Foa* race 4 has 117 *mimps*.

^d^NA, no mimps are within 2.5 kb upstream of the start of the predicted ORF.
