# Supplementary material for: Genomic differences between the new Fusarium oxysporum f. sp. apii (Foa) race 4 on celery, the less virulent Foa races 2 and 3, and the avirulent on celery f. sp. coriandrii
Source: BMC Genomics. 2020 Oct 20;21:730. doi: 10.1186/s12864-020-07141-5 (PMC7576743; doi:10.1186/s12864-020-07141-5)
Supplement: Supplementary file 17 — Additional file 17 The percentage identity of the Secreted In Xylem 1 (SIX1) orthologs in the Foa, Foci, and reference strain [file 12864_2020_7141_MOESM17_ESM.docx]

**Additional file 17.** The percentage identity of the *Secreted In Xylem 1* (*SIX1*) orthologs in the *Foa, Foci,* and reference strain^a^.

| Strain_Location^b^ | *SIX1* ortholog by strain_location for those with two orthologs | | | | | | | |
| --- | --- | --- | --- | --- | --- | --- | --- | --- |
|  | *melonis*^c^ | *Foa*R2_ | *Foa*R3_ | *Foa*R4_ | *Foa*R3_ | *Foa*R4_ | *Foa*R2_ | *Foci* |
|  |  | C9 | C35+ | SS14+ | C35- | SS14- | C26 |  |
|  | Identity, % | | | | | | | |
| *Foa*R2_C9 | 82 |  |  |  |  |  |  |  |
| *Foa*R3_C35:182653+ | 84 | 87 |  |  |  |  |  |  |
| *Foa*R4_SS14:278932+^d^ | 84 | 87 | 100 |  |  |  |  |  |
| *Foa*R3_C35:135680- | 82 | 85 | 86 | 86 |  |  |  |  |
| *Foa*R4_SS14:228927-^d^ | 82 | 85 | 86 | 86 | 100 |  |  |  |
| *Foa*R2_C26 | 82 | 86 | 85 | 85 | 85 | 85 |  |  |
| *Foci*3-2_C15 | 79 | 80 | 82 | 82 | 79 | 79 | 79 |  |
| *Foci*GL306_C17 | 79 | 80 | 82 | 82 | 79 | 79 | 79 | 100 |

^a^*Foa* race 2 (*Foa*R2), *Foa* race 3 (*Foa*R3), and *Foa* race 4 (*Foa*R4) have a *SIX1* in two locations. There are two *Foci* strains (*Foci*3-2 and *Foci*GL306), each with an identical, single ortholog.

^b^Locations of the contig (C) or superscaffold (SS) are shown. Both *Foa* race 3 and 4 orthologs are on the same contig but are on different strands ~500 kbp apart. This is noted with a “+” and “-“ for positive and negative strands respectively.

^c^Ref, Reference *F. oxysporum* f. sp. *melonis SIX1* (GenBank KR811364.1)

^d^Based on RNA TagSeq, both of the *Foa* race 4 *SIX1* orthologs were highly expressed *in planta*, but not *in vitro*. The two *Foa* race 4 copies are on a single host-specific accessory contig. The *Foci*3-2 *SIX1* ortholog is also on a host-specific accessory contig.
