# Supplementary material for: Genomic differences between the new Fusarium oxysporum f. sp. apii (Foa) race 4 on celery, the less virulent Foa races 2 and 3, and the avirulent on celery f. sp. coriandrii
Source: BMC Genomics. 2020 Oct 20;21:730. doi: 10.1186/s12864-020-07141-5 (PMC7576743; doi:10.1186/s12864-020-07141-5)
Supplement: Supplementary file 18 — Additional file 18 Test of PCR primers for Foa races 2 and 4 and Foci on a diversity of Fusarium spp. [file 12864_2020_7141_MOESM18_ESM.docx]

| **Additional file 18** Test of PCR primers for *Foa* races 2 and 4 and *Foci* on a diversity of *Fusarium* spp.^a^ | | | | | | | | |
| --- | --- | --- | --- | --- | --- | --- | --- | --- |
| *Fusarium* taxon^b^ | Isolate ID(s)^c^ | Host ^d^ | Previously published primers [Epstein et al. 2017] | | New primers | | | |
|  |  |  | N4851 for *Foa* race 2 | N3875 for *Foa* race 4 | FOA R2-76k for *Foa* race 2 | FOA R4-447 for *Foa* race 4 | FOCI2-21 for *Foci* | FOCI-g_c31 for *Foci* |
|  |  |  | Amplicon produced?^e^ | | | | | |
| *F. commune,* non-pathogenic on celery (two isolates)^f^ | 250-10A, 250-12A | Celery | **yes** (faint) | no | no | no | no | no |
| *F. commune,* non-pathogenic on celery (two isolates) ^f^ | 252, 271-4 | Celery | no | no | no | no | no | no |
| *F. commune,* non-pathogenic on celery | 270-C | Celery | no | no | no | no | no | no |
| *F. commune,* non-pathogenic on celery | 271-5 | Celery | **yes** | no | no | no | no | no |
| *F. foetens* | NRRL 31852 (=CBS 110286) | Begonia | no | no | no | no | no | NT |
| *F. foetens* | NRRL 38302 | Pine seedling | no | no | no | no | no | NT |
| *F. oxysporum*^j^ | NRRL 54002, (=Fo47) | Soil (Biocontrol strain) | no | no | no | no | no | NT^i^ |
| *F. oxysporum*^j^ | NRRL 32931 | Human | no | no | no | no | no | NT^i^ |
| *F. oxysporum*, non-pathogenic on celery | 295 | Celery | no | no | no | no | no | no |
| *F. oxysporum*, non-pathogenic on celery | 274-3A | Celery | no | no | no | no | no | no |
| *F. oxysporum*, non-pathogenic on celery | 273-2B | Celery | no | **yes** | no | no | no | no |
| *F. oxysporum*, non-pathogenic on celery | 273-1B | Celery | no | **yes** | no | no | no | no |
| *F. oxysporum*, non-pathogenic on celery | 270-B | Celery | no | no | no | no | no | no |
| *F. oxysporum*, non-pathogenic on celery | 258-1A | Celery | no | no | no | no | no | no |
| *F. oxysporum*, non-pathogenic on celery | 251-2 | Celery | no | no | no | no | no | no |
| *F. oxysporum*, non-pathogenic on celery | 249-1A | Celery | yes | no | no | no | no | no |
|  |  |  |  |  |  |  |  |  |
| *F. oxysporum*, non-pathogenic on celery | 223-3B | Celery | no | no | no | no | no | no |
| *F. oxysporum*, non-pathogenic on celery | 222-1B | Celery | no | no | no | no | no | no |
| *F. oxysporum*, non-pathogenic on celery | 220-C | Celery | no | no | no | no | no | no |
| *F. oxysporum*, non-pathogenic on celery | 210-A | Celery | no | no | no | no | no | no |
| *F. oxysporum*, non-pathogenic on celery | 017 | Celery | yes | no | no | no | no | no |
| *F. oxysporum*, non-pathogenic on celery | 221-B | Celery | no | no | no | no | no | no |
| *F. oxysporum*, non-pathogenic on celery | 261-1A | Celery | no | no | no | no | no | no |
| *F. oxysporum*, non-pathogenic on celery | 223-1A | Celery | no | no | no | no | no | no |
| *F. oxysporum*, non-pathogenic on celery | 226-1A | Celery | no | no | no | no | no | no |
| *F. oxysporum*, non-pathogenic on celery | 226-1B | Celery | no | no | no | no | no | no |
| *F. oxysporum*, non-pathogenic on celery | 241-1A | Celery | no | no | no | no | no | no |
| *F. oxysporum* f. sp. *apii* (race 1 presumed) | NRRL 22534 (=CBS 175.35) | Celery | no | no | no | no | no | NT |
| *F. oxysporum* f. sp. *apii* (race 1 presumed) (2 isolates)^f^ | NRRL 36287 (=CBS 176.35), NRRL 36316 (=CBS 184.38) | Celery | no | no | no | no | no | NT |
| *F. oxysporum* f. sp. *apii* (race 1 presumed) | NRRL 36312 (=CBS 183.38) | Celery | no | no | no | no | no | NT |
| *F. oxysporum* f. sp. *apii* race 1 | 268-2 | Celery | no | no | no | no | no | no |
| *F. oxysporum* f. sp. *apii* race 1 | 250-7 | Celery | no | no | no | no | no | no |
| *F. oxysporum* f. sp. *apii* race 1-like haplotype (same as isolate 250-7), but non-pathogenic on celery | 270-A | Celery | no | no | no | no | no | no |
| *F. oxysporum* f. sp. *apii* race 2 (22 isolates)^f^ | 003, 004, 013, 018, 032, 051, 062, 067, 073, 202, 207-A, 225-2A, 226-2A, 240-B, 243-1A, 247-1A, 249-2A, 250-9, 251-1, 258-1B, 269, 272-5A | Celery | yes | no | yes | no | no | no |
| *F. oxysporum* f. sp. apii race 2-like haplotype, but non-pathogenic on celery (18 isolates)^f^ | 005, 071, 074, 205-C, 222-2A, 232, 239-B, 235-A, 241-2C, 244-1A, 244-2B, 246-C, 251-4, 256-1, 262-1, 265-1A, 260-1, 272-2B | Celery | yes | no | yes | no | no | no |
| *F. oxysporum* f. sp. *apii* race 3 | NRRL 38295 | Celery | no | no | no | no | no | no |
| *F. oxysporum* f. sp. *apii* race 4 (11 isolates)^f^ | 274.AC, 273-1A, 273-1C, 283-1.2, 283-4.1, 284-1, 284-5.1, 291, 292-B, 294-B, 296­­­­ | Celery | no | yes | no | yes | no | no |
| *F. oxysporum* f. sp. *apii* race 4, DNA variant^g^ | FoaR4V-313-2.2 | Celery | no | yes | no | yes | no | no |
| *F. oxysporum* f. sp. *apii* race 4, DNA variant^g^ | FoaR4V-7.5B | Coriander | no | yes | no | yes | no | no |
| F. oxysporum f. sp. *coriandrii* (six isolates)^f^ | *Foci*3-2, *Foci*GL306, *Foci*8.1a, *Foci*10E, *Foci*11, *Foci*12A | Coriander | no | **yes** | no | no | yes | yes |
| F. oxysporum f. sp. *coriandrii* (one isolate) | *Foci*10T | Coriander | no | **yes** | no | **yes^h^** | **no^h^** | **no^h^** |
| *F. oxysporum* f. sp. *canariensis* | NRRL 26035 | Date palm | no | no | no | no | no | NT |
| *F. oxysporum* f. sp. *conglutinans* race 2^j^ | NRRL 54008 (=PHW808) | Cabbage | no | no | no | no | no | NT^i^ |
| *F. oxysporum* f. sp. *cubense* | NRRL 36114  (=CBS 102025) | Banana | no | no | no | no | no | NT |
| *F. oxysporum* f. sp. *cubense* tropical race 4^j^ | NRRL 54006 (=IL5) | Banana | no | no | no | no | no | ­­­ NT^i^ |
| *F. oxysporum* f. sp. *fragariae*^j^ | GL-1080 | Strawberry | no | no | no | no | no | NT^i^ |
| *F. oxysporum* f. sp. *lycopersici* race 2^j^ | NRRL 34936 (=4287) | Tomato | no | no | no | no | no | NT^i^ |
| *F. oxysporum* f. sp. *lycopersici* race 3^j^ | NRRL 54003 (=MN25) | Tomato | no | no | no | no | no | NT^i^ |
| *F. oxysporum* f. sp. *lycopersici* | NRRL 26380 | Tomato | no | no | no | no | no | NT |
| *F. oxysporum* f. sp. *lycopersici* | NRRL 36379 (=CBS 249.52, =ETH 5414) | Tomato | no | no | no | no | no | NT |
| *F. oxysporum* f. sp. *lycopersici* | NRRL 36423 (=  CBS 305.91) | Tomato | no | no | no | no | no | NT |
| *F. oxysporum* f. sp. *lycopersici* | NRRL 36425 (=  CBS 307.91) | Tomato | no | no | no | no | no | NT |
| *F. oxysporum* f. sp. *melonis* ^j^ | NRRL 26406 | Melon | no | no | no | no | no | NT^i^ |
| *F. oxysporum* f. sp. *pisi* ^j^ | NRRL 54007 (=HDV247) | Pea | no | no | no | no | no | NT^i^ |
| *F. oxysporum* f. sp. *radicis-lycopersici* ^j^ | NRRL 26381, (=CL57) | Tomato | no | no | no | no | no | NT^i^ |
| *F. oxysporum* f. sp. *radicis-lycopersici* ^j^ | NRRL 26379 | Tomato | no | no | no | no | no | NT |
| *F. oxysporum* f. sp. *raphani* ^j^ | NRRL 54005 (=PHW815) | Radish | no | no | no | **yes^h^** | no | NT^i^ |
| *F. oxysporum* f. sp. *vasinfectum* ^j^ | NRRL 25433 | Cotton | no | no | no | **yes^h^** | no | NT^i^ |

^a^*Foa, F. oxysporum* f. sp. *apii*; *Foci, F. oxysporum* f. sp. *coriandrii*. Primers are described in Table 5. *Foa* are defined as as pathogenic on celery and, to a lesser extent, on coriander. *Foci* are defined as pathogenic on coriander and not on celery.

^b^Except for the five isolates from celery that were in the NRRL collection (which were all collected before 1981), all isolates from celery were from plants that had symptoms of Fusarium yellows. From each symptomatic plant, we typically isolated one isolate that was pathogenic, i.e., that could be classified as a *Foa* race and one from a diversity of *Fusarium oxysporum* species complex Clades 3 and 2 that were non-pathogenic on the celery cultivars that were used for race testing [3].

^c^Isolates with a NRRL number were from the USDA ARS collection. GL-1080 and FociGL306 were obtained from T. Gordon, UC Davis. All other isolates were collected by the authors. Details about the isolates in the authors’ collection from celery are in Epstein et al. [3].

^d^Except for the five isolates from celery that were in the NRRL collection, all isolates from celery were ef1- and igs-sequenced and bioassayed for *Foa* race. All isolates from coriander were ef1- and igs-sequenced and bioassayed for pathogenicity on coriander and celery.

^e^Tests for all isolates included a positive control with either ITS1F-ITS4 or EF1-EF2 and a no-template negative control.

^f^Rows that have multiple isolates in the multiple isolate column had an identical ef1 and igs haplotype, and were tested by PCR individually. The ef1 and igs GenBank accession numbers of all isolates are either in O’Donnell et al. [2], Epstein et al. [3], or in this manuscript.

^g^The “*F. oxysporum* f. sp*. apii* race 4, DNA variant” was isolated from one symptomatic celery and one symptomatic coriander. These isolates are pathogenic on celery cv. Challenger and on coriander cv. Longstanding, and have an indentical ef1 and igs haplotype. That haplotype has one SNP in the ef1; all the other *Foa* races 3 and 4, and six of the seven *Foci* have an identical ef1-igs haplotype without the SNP.

^h^Undesired positives are indicated in bold. However, an isolate from symptomatic celery can be identified as a true positive for *Foa* race 4 based on a positive result with both the FOA R4-447 and the N3875 primers. Identification of the *Foci* from symptomatic coriander is more complex; the majority of isolates from symptomatic coriander were positive with both the *Foci* primers. Amongst the *Foci,* which are defined as pathogenic on coriander and not on celery, isolate 10T was negative for both *Foci* primers and positive for both *Foa* race 4 primers. One isolate from coriander, was a *bona fide* *Foa* race 4, i.e., was pathogenic on celery and on coriander. Differentiation of a *Foci*10T-type from a *Foa* race 4 isolate requires a pathogenicity test.

^i^NT, not tested empirically. For those indicated as NT^i^, there are whole-genome sequences at NCBI; an *in silico* analysis indicated that none would produce this amplicon.

^j^*F. oxysporum* strains that were tested empirically with DNA for the indicated primers and *in silico* from whole genome sequences included the following (GenBank Assemblies in Bold): Fo47, NRRL 54002, [**GCA_000260175.2**](https://www.ncbi.nlm.nih.gov/assembly/GCA_000260175.2)**;** NRRL 32931, [**GCA_000271745.2**](https://www.ncbi.nlm.nih.gov/assembly/GCA_000271745.2)**;** f. sp. *conglutinans* race 2, NRRL 54008 (=PHW808), [**GCA_000260215.2**](https://www.ncbi.nlm.nih.gov/assembly/GCA_000260215.2)**;** f. sp. *cubense* tropical race 4, NRRL 54006 (=IL5), [**GCA_000260195.2**](https://www.ncbi.nlm.nih.gov/assembly/GCA_000260195.2)**;** f. sp. *fragariae* GL-1080, **WIMO00000000 version 2**; *f. sp. lycopersici* race 2, NRRL 34936 (=4287), [**GCA_003315725.1**](https://www.ncbi.nlm.nih.gov/assembly/GCA_003315725.1)**;** *f. sp. lycopersici* race 3, NRRL 54003 (=MN25), [**GCA_000259975.2**](https://www.ncbi.nlm.nih.gov/assembly/GCA_000259975.2)**;** f. sp. *melonis,* NRRL 26406, [**GCA_002318975.1**](https://www.ncbi.nlm.nih.gov/assembly/GCA_002318975.1)**;** f. sp. *pisi,* NRRL 54007 (=HDV247), [**GCA_000260075.2**](https://www.ncbi.nlm.nih.gov/assembly/GCA_000260075.2)**;** f. sp. *radicis-lycopersici,* NRRL 26381, (=CL57), [**GCA_000260155.3**](https://www.ncbi.nlm.nih.gov/assembly/GCA_000260155.3)**;** f. sp. *raphani,* NRRL 54005 (=PHW815), [**GCA_000260235.2**](https://www.ncbi.nlm.nih.gov/assembly/GCA_000260235.2)**;** and f. sp. *vasinfectum,* NRRL 25433, [**GCA_000260175.2**](https://www.ncbi.nlm.nih.gov/assembly/GCA_000260175.2)
