# Supplementary material for: Genomic differences between the new Fusarium oxysporum f. sp. apii (Foa) race 4 on celery, the less virulent Foa races 2 and 3, and the avirulent on celery f. sp. coriandrii
Source: BMC Genomics. 2020 Oct 20;21:730. doi: 10.1186/s12864-020-07141-5 (PMC7576743; doi:10.1186/s12864-020-07141-5)
Supplement: Supplementary file 19 — Additional file 19 Whole genome-sequenced Fusarium spp. included in analyses in GenBank wgs [file 12864_2020_7141_MOESM19_ESM.docx]

Additional file 19: Whole genome-sequenced *Fusarium* spp. included in analyses in GenBank wgs

| Fusarium species | Strain | Assembly ID | No. contigs | Total length, Mbp |
| --- | --- | --- | --- | --- |
| F. acuminatum | F829 | JABEEU01 | 4438 | 46.9 |
| F. acuminatum CS5907 | CS5907 | CBMG01 | 3907 | 44.0 |
| F. acutatum | NRRL 13308 | JAADJF01 | 982 | 43.3 |
| F. agapanthi | NRRL 54464 | LTDI01 | 1842 | 42.0 |
| F. agapanthi | NRRL 31653 | LUFC02 | 2350 | 41.3 |
| F. albidum | NRRL_22152 | JABFEP01 | 5413 | 36.9 |
| F. albosuccineum | NRRL 20459 | JAADYS01 | 4197 | 50.9 |
| F. algeriense | NRRL 66648 | PVPY01 | 3323 | 50.0 |
| F. algeriense | NRRL 66647 | PVPZ01 | 3535 | 48.9 |
| F. ambrosium | NRRL 20438 | NIZV01 | 1366 | 49.0 |
| F. anguioides | NRRL 25385 | JAALXK01 | 743 | 38.9 |
| F. anthophilum | NRRL 25214 | JABEVY01 | 1118 | 45.8 |
| F. armeniacum | NRRL 25141 | JABFER01 | 429 | 37.1 |
| F. asiaticum | NRRL6101 | LHTY01 | 265 | 36.5 |
| F. asiaticum | NRRL28720 | LHTZ01 | 353 | 36.4 |
| F. asiaticum | NRRL 26156 | JABFEQ01 | 490 | 36.5 |
| F. austroafricanum | NRRL 53441 | JAADJG01 | 1963 | 45.7 |
| F. austroamericanum | 28FRS | VSSU01 | 534 | 37.5 |
| F. austroamericanum | 3FSP | VSSV01 | 619 | 37.3 |
| F. austroamericanum | NRRL 2903 | JAAMOD01 | 899 | 36.9 |
| F. avenaceum | Fa05001 | JPYM01 | 83 | 41.6 |
| F. avenaceum | FaLH03 | JQGD01 | 105 | 42.7 |
| F. avenaceum | FaLH27 | JQGE01 | 78 | 43.2 |
| F. avenaceum | KA13 | JABCRA01 | 34 | 41.7 |
| F. avenaceum | NRRL 13321 | JABSSY01 | 778 | 41.7 |
| F. aywerte | NRRL 25410 | JABCQV01 | 911 | 36.0 |
| F. azukicola | NRRL 54364 | MAEG01 | 32428 | 51.3 |
| F. babinda | NRRL 25533 | JAALXL01 | 1090 | 43.8 |
| F. babinda | NRRL 25539 | JABCKA01 | 2053 | 42.9 |
| F. bactridioides | NRRL 66639 | JACCKW01 | 1826 | 42.9 |
| F. begoniae | NRRL 25300 | JAAOAG01 | 1002 | 43.9 |
| F. beomiforme | NRRL 25174 | PVQB02 | 1868 | 46.5 |
| F. brasilicum | NRRL 31281 | JABCJS01 | 627 | 36.8 |
| F. brasiliense | NRRL 31757 | MAEC01 | 22229 | 49.4 |
| F. brevicatenulatum | NRRL 25447 | JABEEJ01 | 1763 | 42.5 |
| F. bulbicola | NRRL 22947 | JAAOAH01 | 3116 | 42.3 |
| F. bulbicola | NRRL 25176 | JAAOAI01 | 1719 | 43.6 |
| F. burgessii | NRRL 66654 | PVQA01 | 3437 | 49.3 |
| F. caatingaense | NRRL 66470 | JABKKP01 | 1238 | 37.8 |
| F. camptoceras | NRRL 13381 | QGED01 | 467 | 36.8 |
| F. cerealis | isolate Fcer1134NY13 | JAAVVS01 | 3537 | 37.2 |
| F. circinatum | GL1327 | JRVE01 | 909 | 42.5 |
| F. circinatum | FSP 34 | AYJV02 | 585 | 43.9 |
| F. circinatum | isolate KS17 | LQBB01 | 6033 | 46.3 |
| F. circinatum | isolate V | JABACP01 | 426 | 46.3 |
| F. circinatum | isolate S | JABACQ01 | 481 | 46.1 |
| F. circinatum | isolate LB | JABAYB01 | 207 | 45.3 |
| F. circinatum | NRRL 25331 | JAAQPE01 | 1223 | 42.6 |
| F. citri | NRRL 66334 (ITEM 10392) | QHHH01 | 445 | 37.9 |
| F. clavum | CS3069 | CBMI01 | 5111 | 38.1 |
| F. clavum | NRRL 66337 (ITEM 11348) | QGEC01 | 854 | 38.6 |
| F. coffeatum | FIESC_28 | QKXC01 | 550 | 37.8 |
| F. coffeatum | NRRL 66322 (ITEM 1616) | QGDX01 | 654 | 37.0 |
| F. coicis | NRRL 66233 | JAAOAJ01 | 1267 | 42.7 |
| F. commune | JCM 11502 | BCHB01 | 19 | 46.2 |
| F. commune | NRRL 28387 | JABFES01 | 1931 | 48.1 |
| F. compactum | NRRL 13829 | JABFET01 | 516 | 37.9 |
| F. concolor | NRRL 13459 | JABCJY01 | 3171 | 49.6 |
| F. continuum | NRRL 66286 | JABCKB01 | 2038 | 38.1 |
| F. cortaderiae | 1FP | VSSW01 | 573 | 37.3 |
| F. cortaderiae | NRRL 29297 | JABCJT01 | 639 | 36.7 |
| F. culmorum |  | FJUU01 | 2 | 5.0 |
| F. culmorum | PV | PVEM01 | 36 | 38.1 |
| F. culmorum | NRRL 25475 | JABFEU01 | 799 | 36.6 |
| F. culmorum CS7071 | CS7071 | CBMH01 | 2274 | 37.7 |
| F. cuneirostrum | NRRL 31157 | MAEA01 | 18692 | 49.0 |
| F. decemcellulare | NRRL 13412 | JAAGWO01 | 3482 | 53.7 |
| F. denticulatum | NRRL 25311 | JAAOAK01 | 909 | 43.2 |
| F. dimerum | NRRL 20691 | JABGLY01 | 2223 | 37.6 |
| F. dlaminii | NRRL 13164 | JAAOAL01 | 484 | 44.1 |
| F. domesticum | NRRL 29976 | JABFEV01 | 3048 | 32.6 |
| F. equiseti | CS5819 | MTPY01 | 267 | 39.6 |
| F. equiseti | isolate D25-1 | QOHM01 | 16 | 40.8 |
| F. equiseti | D25-1 | QJGT01 | 205 | 40.6 |
| F. equiseti | NRRL 66338 (ITEM 11363) | QGEB01 | 643 | 40.0 |
| F. euwallaceae | HFEW-16-IV-019 | NHTE02 | 287 | 48.3 |
| F. euwallaceae | UCR1854 | MIKF01 | 1773 | 50.6 |
| F. falciforme | NRRL 43529 | JABEEK01 | 3427 | 48.2 |
| F. flagelliforme | NRRL 13405 | PXXK01 | 1073 | 39.6 |
| F. flagelliforme | NRRL 66336 (ITEM 11294) | QHHI01 | 1363 | 40.4 |
| F. foetens | NRRL 38302 | JABFMM01 | 3842 | 46.3 |
| F. fracticaudum | CBS 137234 | PDNT01 | 50 | 46.3 |
| F. fractiflexum | NRRL 43689 | JABSTE01 | 1442 | 44.4 |
| F. fujikuroi | FGSC 8932 | JRVF01 | 835 | 43.1 |
| F. fujikuroi | KSU X-10626 | JRVG01 | 187 | 43.1 |
| F. fujikuroi | KSU 3368 | JRVH01 | 2959 | 43.5 |
| F. fujikuroi | B14 | FMSL01 | 66 | 44.0 |
| F. fujikuroi | FSU48 | FMSM01 | 182 | 46.1 |
| F. fujikuroi |  | FMJS01 | 318 | 44.3 |
| F. fujikuroi | E282 | FMJT01 | 227 | 46.1 |
| F. fujikuroi | C1995 | FMJU01 | 86 | 45.8 |
| F. fujikuroi | m567 | FMJV01 | 241 | 44.0 |
| F. fujikuroi | MRC2276 | FMJW01 | 28 | 45.0 |
| F. fujikuroi | NCIM1100 | FMJX01 | 240 | 45.3 |
| F. fujikuroi | FUS01 | NCQQ02 | 881 | 49.1 |
| F. fujikuroi | isolate F250 | MBPO01 | 3872 | 42.5 |
| F. fujikuroi | ke1 | VJME01 | 502 | 48.7 |
| F. fujikuroi | NRRL 66331 | JABSTJ01 | 1506 | 42.5 |
| F. fujikuroi B14 | B14 | ANFV01 | 455 | 43.8 |
| F. gaditjirri | NRRL 45417 | JABFAI01 | 834 | 41.9 |
| F. globosum | NRRL 26131 | JAAQPF01 | 1696 | 44.6 |
| F. graminearum | CS3005 | JATU01 | 424 | 36.6 |
| F. graminearum | 233423 | LAJZ01 | 869 | 36.5 |
| F. graminearum | 241165 | LAKA01 | 486 | 36.6 |
| F. graminearum | DAOM180378 | LHUC01 | 520 | 36.4 |
| F. graminearum | NRRL28336 | LHUD01 | 303 | 36.7 |
| F. graminearum | ITEM 124 | NQOC01 | 67 | 36.9 |
| F. graminearum | FN009 | UCPH01 | 715 | 36.8 |
| F. graminearum | MDC_Fg1 | UIHA01 | 96 | 36.8 |
| F. graminearum | MDC_Fg13 | CABDWO01 | 283 | 36.8 |
| F. graminearum | FG078 | VIGE01 | 335 | 36.8 |
| F. graminearum | TaB10 | JABCRB01 | 54 | 36.8 |
| F. graminearum PH-1 | PH-1; NRRL 31084 | AACM02 | 435 | 36.3 |
| F. guttiforme | NRRL 22945 | JAAQRL01 | 1384 | 43.3 |
| F. hainanense | NRRL 66475 | JABFEW01 | 667 | 36.6 |
| F. heterosporum | NRRL 20692 | JAAGWP01 | 977 | 35.0 |
| F. heterosporum | NRRL 20693 | JAAGWQ01 | 793 | 35.7 |
| F. hostae | Hy14 | NJCQ01 | 3725 | 54.4 |
| F. hostae | Hy9 | NJCR01 | 3686 | 54.6 |
| F. hostae | NRRL 29888 | JABCJX01 | 3090 | 46.0 |
| F. humuli | NRRL 66335 (ITEM 10395) | QHHG01 | 602 | 39.0 |
| F. humuli | NRRL 66339 (ITEM 11401) | QHKN01 | 735 | 39.2 |
| F. humuli | NRRL 66681 | JABSTA01 | 1121 | 39.5 |
| F. illudens | NRRL 22090 | JABFEX01 | 3457 | 40.3 |
| F. incarnatum | MOD1-FUNGI18 | RBBZ01 | 340 | 38.3 |
| F. incarnatum | MOD1-FUNGI17 | RBJE01 | 325 | 38.3 |
| F. incarnatum | NRRL 66325 (ITEM 7155) | QGDZ01 | 381 | 37.5 |
| F. irregulare | NRRL 31160 | QGEA01 | 627 | 37.4 |
| F. kuroshium | isolate UCR3666 | NKUJ01 | 1403 | 46.6 |
| F. kyushuense | NRRL 25348 | JABCJU01 | 325 | 36.0 |
| F. langsethiae | Fl201059 | JXCE01 | 1586 | 37.5 |
| F. longipes | NRRL 20695 | PXOG01 | 544 | 35.3 |
| F. longipes | NRRL 13317 | JABFEY01 | 285 | 35.3 |
| F. longipes | NRRL 13368 | JABFEZ01 | 492 | 35.5 |
| F. longipes | NRRL 13374 | JACCKV01 | 393 | 35.3 |
| F. luffae | NRRL 66473 | JABCJV01 | 1049 | 37.4 |
| F. mangiferae | MRC7560 | FCQH01 | 254 | 46.3 |
| F. mangiferae | NRRL 25226 | JABSTF01 | 2060 | 48.6 |
| F. meridionale | NRRL28721 | LHUA01 | 676 | 36.5 |
| F. meridionale | NRRL28723 | LHUB01 | 287 | 36.4 |
| F. meridionale | 38FSP | VSSX01 | 694 | 36.9 |
| F. metavorans | FSSC_6 | LWBZ01 | 103 | 46.9 |
| F. mexicanum | NRRL 53147 | JAAOAM01 | 958 | 44.0 |
| F. mundagurra | NRRL 66235 | JAAOAN01 | 1616 | 49.1 |
| F. musae | NRRL 25059 | JACCKU01 | 1114 | 42.5 |
| F. nanum | NRRL 66324 (ITEM 6748) | QGDY01 | 530 | 37.2 |
| F. napiforme | NRRL 25196 | JAAOAO01 | 1411 | 42.1 |
| F. nematophilum | NRRL 54600 | JABFFA01 | 5027 | 52.5 |
| F. neocosmosporiellum | NRRL 22166 | SSHR01 | 5494 | 54.0 |
| F. newnesense | NRRL 66241 | JABCJW01 | 5456 | 48.8 |
| F. nisikadoi | NRRL 25179 | JABFFB01 | 1085 | 40.9 |
| F. nurragi | NRRL 36452 | JAALXI01 | 854 | 35.5 |
| F. nygamai | MRC8546 | LBNR01 | 409 | 51.6 |
| F. nygamai | CS10214 | MTQA01 | 991 | 50.2 |
| F. nygamai | NRRL 66327 | JAAOAP01 | 2753 | 48.5 |
| F. odoratissimum | race 4 | AMGQ01 | 3834 | 48.8 |
| F. odoratissimum NRRL 54006 | 54006 | AGND01 | 716 | 46.4 |
| F. oxysporum | UASWS AC1 | JNNQ01 | 1166 | 50.6 |
| F. oxysporum | FoMN14 | MALU01 | 1978 | 49.0 |
| F. oxysporum | IMV 00293 | MSJJ02 | 876 | 51.3 |
| F. oxysporum | Tu58 | NJBT01 | 1277 | 50.4 |
| F. oxysporum | KOD888 | NJBU01 | 11382 | 59.4 |
| F. oxysporum | KOD887 | NJBV01 | 1275 | 50.4 |
| F. oxysporum | KOD886 | NJBW01 | 488 | 47.2 |
| F. oxysporum | V64-1 | FMJY01 | 49 | 49.1 |
| F. oxysporum | CS5870 | MTPZ01 | 1295 | 51.4 |
| F. oxysporum | VEG-01C2 | PXUN01 | 3653 | 48.5 |
| F. oxysporum | VEG-01C1 | PXUO01 | 2969 | 48.8 |
| F. oxysporum | Fo_A13 | MRCX01 | 3121 | 54.8 |
| F. oxysporum | Fo_A28 | MRCY01 | 2373 | 53.0 |
| F. oxysporum | Fo_CB3 | MRCZ01 | 1719 | 50.5 |
| F. oxysporum | Fo_PG | MRDA01 | 920 | 50.3 |
| F. oxysporum | MOD1-FUNGI16 | RBCA01 | 944 | 44.4 |
| F. oxysporum | MOD1-FUNGI11 | RBCF01 | 1377 | 46.3 |
| F. oxysporum | MOD1-FUNGI9 | RBCG01 | 2370 | 47.5 |
| F. oxysporum | MOD1-FUNGI10 | RBJF01 | 1950 | 46.3 |
| F. oxysporum | ISS-F3 | QUWZ01 | 2964 | 53.1 |
| F. oxysporum | ISS-F4 | QUXA01 | 3405 | 53.3 |
| F. oxysporum | RBG5689 | WGOF01 | 4075 | 50.5 |
| F. oxysporum | RBG5714 | WGOG01 | 7964 | 55.7 |
| F. oxysporum | RBG5783 | WGOH01 | 8531 | 51.1 |
| F. oxysporum | RBG5831 | WGOI01 | 4723 | 51.9 |
| F. oxysporum | RBG5833 | WGOJ01 | 11714 | 57.2 |
| F. oxysporum | RBG5836 | WGOK01 | 7253 | 53.0 |
| F. oxysporum | RBG5844 | WGOL01 | 5223 | 52.4 |
| F. oxysporum | RBG5862 | WGOM01 | 4352 | 50.8 |
| F. oxysporum | RBG6301 | WGON01 | 5001 | 52.4 |
| F. oxysporum | RBG6309 | WGOO01 | 4303 | 48.7 |
| F. oxysporum | RBG6313 | WGOP01 | 3298 | 45.8 |
| F. oxysporum | RBG6324 | WGOQ01 | 12866 | 51.7 |
| F. oxysporum | RBG6358 | WGOR01 | 2995 | 50.9 |
| F. oxysporum | RBG6396 | WGOS01 | 5820 | 49.2 |
| F. oxysporum | RBG6397 | WGOT01 | 6674 | 51.8 |
| F. oxysporum | RBG6398 | WGOU01 | 2261 | 47.8 |
| F. oxysporum | RBG6400 | WGOV01 | 6304 | 49.8 |
| F. oxysporum | RBG6406 | WGOW01 | 23217 | 63.1 |
| F. oxysporum | RBG6416 | WGOX01 | 5786 | 50.4 |
| F. oxysporum | RBG6417 | WGOY01 | 5340 | 51.8 |
| F. oxysporum | RBG6418 | WGOZ01 | 5516 | 52.8 |
| F. oxysporum | RBG6419 | WGPA01 | 3403 | 49.1 |
| F. oxysporum | RBG6420 | WGPB01 | 3580 | 48.4 |
| F. oxysporum | RBG6421 | WGPC01 | 7415 | 50.4 |
| F. oxysporum | RBG6422 | WGPD01 | 5462 | 49.5 |
| F. oxysporum | RBG6423 | WGPE01 | 9155 | 60.7 |
| F. oxysporum | RBG6425 | WGPF01 | 4601 | 50.4 |
| F. oxysporum | RBG6429 | WGPG01 | 7453 | 49.5 |
| F. oxysporum | RBG6431 | WGPH01 | 2707 | 48.2 |
| F. oxysporum | RBG6433 | WGPI01 | 6441 | 53.7 |
| F. oxysporum | RBG6442 | WGPJ01 | 8036 | 52.3 |
| F. oxysporum | RBG6444 | WGPK01 | 9085 | 54.4 |
| F. oxysporum | RBG6448 | WGPL01 | 3571 | 48.0 |
| F. oxysporum | RBG6450 | WGPM01 | 5755 | 48.6 |
| F. oxysporum | RBG6454 | WGPN01 | 12368 | 53.0 |
| F. oxysporum | RBG6462 | WGPO01 | 4614 | 50.3 |
| F. oxysporum | RBG6464 | WGPP01 | 11735 | 56.2 |
| F. oxysporum | RBG6466 | WGPQ01 | 4619 | 49.3 |
| F. oxysporum | RBG6475 | WGPR01 | 7651 | 59.1 |
| F. oxysporum | RBG6477 | WGPS01 | 7838 | 49.4 |
| F. oxysporum | RBG6480 | WGPT01 | 7934 | 55.8 |
| F. oxysporum | RBG6494 | WGPU01 | 7564 | 50.6 |
| F. oxysporum | RBG6499 | WGPV01 | 8772 | 52.3 |
| F. oxysporum | RBG6503 | WGPW01 | 5720 | 52.0 |
| F. oxysporum | RBG6505 | WGPX01 | 12642 | 54.5 |
| F. oxysporum | RBG7064 | WGPY01 | 9049 | 51.2 |
| F. oxysporum | RBG7070 | WGPZ01 | 11791 | 54.1 |
| F. oxysporum | VPRI10351 | WGQA01 | 2962 | 47.7 |
| F. oxysporum | VPRI10358 | WGQB01 | 5509 | 53.8 |
| F. oxysporum | VPRI10403 | WGQC01 | 1488 | 48.0 |
| F. oxysporum | VPRI10405 | WGQD01 | 4639 | 52.1 |
| F. oxysporum | VPRI10408 | WGQE01 | 3187 | 49.1 |
| F. oxysporum | VPRI10605 | WGQF01 | 3019 | 51.4 |
| F. oxysporum | VPRI11235 | WGQG01 | 4901 | 50.4 |
| F. oxysporum | VPRI11409 | WGQH01 | 7718 | 53.4 |
| F. oxysporum | VPRI11681 | WGQI01 | 10624 | 50.2 |
| F. oxysporum | VPRI11762 | WGQJ01 | 892 | 47.5 |
| F. oxysporum | VPRI12300 | WGQK01 | 4286 | 54.1 |
| F. oxysporum | VPRI13039 | WGQL01 | 4932 | 51.3 |
| F. oxysporum | VPRI16234 | WGQM01 | 5786 | 54.5 |
| F. oxysporum | VPRI16235 | WGQN01 | 7972 | 55.4 |
| F. oxysporum | VPRI16963 | WGQO01 | 3454 | 49.6 |
| F. oxysporum | VPRI17577 | WGQP01 | 1626 | 48.3 |
| F. oxysporum | VPRI17796 | WGQQ01 | 2778 | 50.0 |
| F. oxysporum | VPRI19293 | WGQR01 | 3353 | 52.5 |
| F. oxysporum | VPRI31638 | WGQS01 | 4144 | 53.6 |
| F. oxysporum | VPRI32264 | WGQT01 | 6149 | 54.6 |
| F. oxysporum | VPRI32287 | WGQU01 | 2446 | 45.4 |
| F. oxysporum | VPRI32288 | WGQV01 | 1495 | 46.9 |
| F. oxysporum | VPRI32289 | WGQW01 | 24336 | 57.9 |
| F. oxysporum | VPRI32441 | WGQX01 | 3584 | 46.3 |
| F. oxysporum | VPRI32442 | WGQY01 | 6771 | 54.9 |
| F. oxysporum | VPRI41207 | WGQZ01 | 2808 | 46.9 |
| F. oxysporum | VPRI41208 | WGRA01 | 1929 | 46.1 |
| F. oxysporum | VPRI41778 | WGRB01 | 6155 | 54.0 |
| F. oxysporum | VPRI41836 | WGRC01 | 6179 | 54.5 |
| F. oxysporum | VPRI41884 | WGRD01 | 4931 | 52.7 |
| F. oxysporum | VPRI41920 | WGRE01 | 4233 | 49.9 |
| F. oxysporum | VPRI42109 | WGRF01 | 2063 | 48.0 |
| F. oxysporum | VPRI42117 | WGRG01 | 3897 | 49.2 |
| F. oxysporum | VPRI42118 | WGRH01 | 4826 | 54.2 |
| F. oxysporum | VPRI42119 | WGRI01 | 5714 | 55.5 |
| F. oxysporum | VPRI42176 | WGRJ01 | 4898 | 48.0 |
| F. oxysporum | VPRI42180 | WGRK01 | 6639 | 52.4 |
| F. oxysporum | VPRI42181 | WGRL01 | 1976 | 45.8 |
| F. oxysporum | VPRI42190 | WGRM01 | 2110 | 50.1 |
| F. oxysporum | VPRI42198 | WGRN01 | 3244 | 50.4 |
| F. oxysporum | VPRI42252 | WGRO01 | 4801 | 51.7 |
| F. oxysporum | VPRI42253 | WGRP01 | 1101 | 48.7 |
| F. oxysporum | VPRI42327 | WGRQ01 | 7345 | 55.5 |
| F. oxysporum | VPRI42339 | WGRR01 | 3249 | 56.7 |
| F. oxysporum | VPRI42420 | WGRS01 | 5371 | 53.2 |
| F. oxysporum | VPRI42760 | WGRT01 | 2073 | 51.2 |
| F. oxysporum | VPRI42882 | WGRU01 | 3786 | 51.1 |
| F. oxysporum | VPRI42888 | WGRV01 | 5155 | 52.3 |
| F. oxysporum | VPRI42889 | WGRW01 | 2962 | 49.8 |
| F. oxysporum | VPRI43193 | WGRX01 | 6063 | 51.1 |
| F. oxysporum | VPRI43194 | WGRY01 | 3931 | 50.3 |
| F. oxysporum | VPRI43195 | WGRZ01 | 2843 | 49.9 |
| F. oxysporum | MRL8996 | VLJC01 | 252 | 50.1 |
| F. oxysporum | EtdFoc-1 | WESF01 | 4639 | 59.0 |
| F. oxysporum | EtdFoc-10 | WESG01 | 2289 | 54.5 |
| F. oxysporum | EtdFoc-102 | WESH01 | 14465 | 54.6 |
| F. oxysporum | EtdFoc-109 | WESI01 | 5364 | 53.4 |
| F. oxysporum | EtdFoc-113 | WESJ01 | 24914 | 61.1 |
| F. oxysporum | EtdFoc-13 | WESK01 | 3196 | 53.3 |
| F. oxysporum | EtdFoc-136 | WESL01 | 6480 | 54.3 |
| F. oxysporum | EtdFoc-139 | WESM01 | 34591 | 50.2 |
| F. oxysporum | EtdFoc-155 | WESN01 | 1944 | 55.0 |
| F. oxysporum | EtdFoc-158 | WESO01 | 3013 | 52.1 |
| F. oxysporum | EtdFoc-16 | WESP01 | 5985 | 58.3 |
| F. oxysporum | EtdFoc-160 | WESQ01 | 5118 | 54.3 |
| F. oxysporum | EtdFoc-167 | WESR01 | 1378 | 56.7 |
| F. oxysporum | EtdFoc-17 | WESS01 | 27789 | 63.1 |
| F. oxysporum | EtdFoc-174 | WEST01 | 25995 | 52.7 |
| F. oxysporum | EtdFoc-178 | WESU01 | 7839 | 59.6 |
| F. oxysporum | EtdFoc-18 | WESV01 | 29403 | 45.3 |
| F. oxysporum | EtdFoc-183 | WESW01 | 6667 | 56.2 |
| F. oxysporum | EtdFoc-184 | WESX01 | 2161 | 59.3 |
| F. oxysporum | EtdFoc-185 | WESY01 | 4759 | 59.1 |
| F. oxysporum | EtdFoc-186 | WESZ01 | 1515 | 59.3 |
| F. oxysporum | EtdFoc-19 | WETA01 | 2428 | 59.2 |
| F. oxysporum | EtdFoc-190 | WETB01 | 6304 | 62.7 |
| F. oxysporum | EtdFoc-193 | WETC01 | 7090 | 60.8 |
| F. oxysporum | EtdFoc-195 | WETD01 | 2768 | 59.4 |
| F. oxysporum | EtdFoc-198 | WETE01 | 10886 | 63.5 |
| F. oxysporum | EtdFoc-2 | WETF01 | 12359 | 52.8 |
| F. oxysporum | EtdFoc-203 | WETG01 | 2697 | 55.4 |
| F. oxysporum | EtdFoc-204 | WETH01 | 3888 | 60.5 |
| F. oxysporum | EtdFoc-208 | WETI01 | 2429 | 57.0 |
| F. oxysporum | EtdFoc-209 | WETJ01 | 8039 | 56.5 |
| F. oxysporum | EtdFoc-213 | WETK01 | 9839 | 52.5 |
| F. oxysporum | EtdFoc-216 | WETL01 | 1797 | 52.5 |
| F. oxysporum | EtdFoc-218 | WETM01 | 1864 | 53.0 |
| F. oxysporum | EtdFoc-219 | WETN01 | 1690 | 52.4 |
| F. oxysporum | EtdFoc-22 | WETO01 | 1060 | 50.7 |
| F. oxysporum | EtdFoc-221 | WETP01 | 2944 | 53.6 |
| F. oxysporum | EtdFoc-222 | WETQ01 | 3761 | 59.4 |
| F. oxysporum | EtdFoc-223 | WETR01 | 1903 | 53.6 |
| F. oxysporum | EtdFoc-228 | WETS01 | 18787 | 54.5 |
| F. oxysporum | EtdFoc-229 | WETT01 | 2792 | 52.6 |
| F. oxysporum | EtdFoc-23 | WETU01 | 1933 | 53.4 |
| F. oxysporum | EtdFoc-233 | WETV01 | 5514 | 56.8 |
| F. oxysporum | EtdFoc-234 | WETW01 | 1561 | 52.2 |
| F. oxysporum | EtdFoc-236 | WETX01 | 1906 | 53.9 |
| F. oxysporum | EtdFoc-237 | WETY01 | 23533 | 50.5 |
| F. oxysporum | EtdFoc-240 | WETZ01 | 272 | 40.2 |
| F. oxysporum | EtdFoc-243 | WEUA01 | 6542 | 57.9 |
| F. oxysporum | EtdFoc-245 | WEUB01 | 3400 | 53.8 |
| F. oxysporum | EtdFoc-247 | WEUC01 | 7745 | 62.3 |
| F. oxysporum | EtdFoc-248 | WEUD01 | 2494 | 50.5 |
| F. oxysporum | EtdFoc-25 | WEUE01 | 30013 | 46.0 |
| F. oxysporum | EtdFoc-250 | WEUF01 | 11764 | 62.8 |
| F. oxysporum | EtdFoc-253 | WEUG01 | 15576 | 50.2 |
| F. oxysporum | EtdFoc-256 | WEUH01 | 2941 | 53.7 |
| F. oxysporum | EtdFoc-258 | WEUI01 | 1685 | 51.4 |
| F. oxysporum | EtdFoc-259 | WEUJ01 | 2294 | 53.1 |
| F. oxysporum | EtdFoc-29 | WEUK01 | 11694 | 55.8 |
| F. oxysporum | EtdFoc-30 | WEUL01 | 1766 | 52.2 |
| F. oxysporum | EtdFoc-34 | WEUM01 | 18766 | 51.9 |
| F. oxysporum | EtdFoc-35 | WEUN01 | 18965 | 52.7 |
| F. oxysporum | EtdFoc-38 | WEUO01 | 2323 | 59.1 |
| F. oxysporum | EtdFoc-39 | WEUP01 | 1287 | 51.7 |
| F. oxysporum | EtdFoc-4 | WEUQ01 | 15289 | 52.3 |
| F. oxysporum | EtdFoc-46 | WEUR01 | 2393 | 53.1 |
| F. oxysporum | EtdFoc-47 | WEUS01 | 3804 | 52.1 |
| F. oxysporum | EtdFoc-48 | WEUT01 | 2806 | 54.5 |
| F. oxysporum | EtdFoc-49 | WEUU01 | 3907 | 52.4 |
| F. oxysporum | EtdFoc-5 | WEUV01 | 2295 | 52.2 |
| F. oxysporum | EtdFoc-52 | WEUW01 | 8137 | 53.5 |
| F. oxysporum | EtdFoc-55 | WEUX01 | 3236 | 54.8 |
| F. oxysporum | EtdFoc-58 | WEUY01 | 3653 | 61.4 |
| F. oxysporum | EtdFoc-6 | WEUZ01 | 4160 | 57.5 |
| F. oxysporum | EtdFoc-61 | WEVA01 | 3836 | 52.5 |
| F. oxysporum | EtdFoc-63 | WEVB01 | 5198 | 61.1 |
| F. oxysporum | EtdFoc-68 | WEVC01 | 3070 | 59.9 |
| F. oxysporum | EtdFoc-69 | WEVD01 | 23250 | 51.1 |
| F. oxysporum | EtdFoc-7 | WEVE01 | 3798 | 52.8 |
| F. oxysporum | EtdFoc-72 | WEVF01 | 5193 | 53.9 |
| F. oxysporum | EtdFoc-75 | WEVG01 | 34308 | 54.0 |
| F. oxysporum | EtdFoc-79 | WEVH01 | 2717 | 53.2 |
| F. oxysporum | EtdFoc-8 | WEVI01 | 1900 | 52.6 |
| F. oxysporum | EtdFoc-86 | WEVJ01 | 6322 | 53.7 |
| F. oxysporum | EtdFoc-87 | WEVK01 | 2186 | 52.9 |
| F. oxysporum | EtdFoc-88 | WEVL01 | 1790 | 52.8 |
| F. oxysporum | EtdFoc-92 | WEVM01 | 12352 | 59.3 |
| F. oxysporum | EtdFoc-94 | WEVN01 | 10092 | 53.1 |
| F. oxysporum | EtdFoc-99 | WEVO01 | 17061 | 52.2 |
| F. oxysporum | EthFoc-10 | WEVP01 | 14538 | 53.1 |
| F. oxysporum | EthFoc-100 | WEVQ01 | 23909 | 46.8 |
| F. oxysporum | EthFoc-101 | WEVR01 | 2006 | 52.8 |
| F. oxysporum | EthFoc-102 | WEVS01 | 3240 | 53.4 |
| F. oxysporum | EthFoc-106 | WEVT01 | 2217 | 51.8 |
| F. oxysporum | EthFoc-11 | WEVU01 | 1648 | 52.5 |
| F. oxysporum | EthFoc-111 | WEVV01 | 1785 | 52.7 |
| F. oxysporum | EthFoc-113 | WEVW01 | 1727 | 51.8 |
| F. oxysporum | EthFoc-116 | WEVX01 | 8532 | 60.0 |
| F. oxysporum | EthFoc-118 | WEVY01 | 2031 | 53.4 |
| F. oxysporum | EthFoc-119 | WEVZ01 | 906 | 49.2 |
| F. oxysporum | EthFoc-12 | WEWA01 | 2584 | 52.8 |
| F. oxysporum | EthFoc-120 | WEWB01 | 1842 | 52.4 |
| F. oxysporum | EthFoc-122 | WEWC01 | 9926 | 57.4 |
| F. oxysporum | EthFoc-124 | WEWD01 | 1779 | 52.0 |
| F. oxysporum | EthFoc-125 | WEWE01 | 2239 | 52.8 |
| F. oxysporum | EthFoc-126 | WEWF01 | 1635 | 52.2 |
| F. oxysporum | EthFoc-127 | WEWG01 | 1819 | 52.2 |
| F. oxysporum | EthFoc-129 | WEWH01 | 2050 | 52.3 |
| F. oxysporum | EthFoc-13 | WEWI01 | 2151 | 53.2 |
| F. oxysporum | EthFoc-130 | WEWJ01 | 1523 | 51.3 |
| F. oxysporum | EthFoc-131 | WEWK01 | 1696 | 51.1 |
| F. oxysporum | EthFoc-133 | WEWL01 | 360 | 42.7 |
| F. oxysporum | EthFoc-135 | WEWM01 | 2778 | 51.9 |
| F. oxysporum | EthFoc-136 | WEWN01 | 1654 | 52.6 |
| F. oxysporum | EthFoc-138 | WEWO01 | 31481 | 40.3 |
| F. oxysporum | EthFoc-139 | WEWP01 | 27775 | 46.1 |
| F. oxysporum | EthFoc-144 | WEWQ01 | 13369 | 58.6 |
| F. oxysporum | EthFoc-146 | WEWR01 | 19291 | 49.6 |
| F. oxysporum | EthFoc-148 | WEWS01 | 5198 | 55.0 |
| F. oxysporum | EthFoc-158 | WEWT01 | 31261 | 51.1 |
| F. oxysporum | EthFoc-159 | WEWU01 | 34674 | 41.5 |
| F. oxysporum | EthFoc-16 | WEWV01 | 2209 | 52.5 |
| F. oxysporum | EthFoc-164 | WEWW01 | 26531 | 41.4 |
| F. oxysporum | EthFoc-165 | WEWX01 | 36743 | 40.4 |
| F. oxysporum | EthFoc-167 | WEWY01 | 22139 | 49.8 |
| F. oxysporum | EthFoc-18 | WEWZ01 | 21751 | 54.0 |
| F. oxysporum | EthFoc-2 | WEXA01 | 1348 | 52.2 |
| F. oxysporum | EthFoc-20 | WEXB01 | 20233 | 50.5 |
| F. oxysporum | EthFoc-21 | WEXC01 | 1840 | 53.1 |
| F. oxysporum | EthFoc-22 | WEXD01 | 2133 | 53.4 |
| F. oxysporum | EthFoc-23 | WEXE01 | 2310 | 54.2 |
| F. oxysporum | EthFoc-25 | WEXF01 | 2529 | 53.6 |
| F. oxysporum | EthFoc-26 | WEXG01 | 20993 | 49.2 |
| F. oxysporum | EthFoc-27 | WEXH01 | 5931 | 54.4 |
| F. oxysporum | EthFoc-28 | WEXI01 | 18218 | 51.9 |
| F. oxysporum | EthFoc-29 | WEXJ01 | 17492 | 53.0 |
| F. oxysporum | EthFoc-3 | WEXK01 | 2410 | 52.7 |
| F. oxysporum | EthFoc-30 | WEXL01 | 7643 | 58.2 |
| F. oxysporum | EthFoc-34 | WEXM01 | 2105 | 54.4 |
| F. oxysporum | EthFoc-36 | WEXN01 | 1380 | 51.9 |
| F. oxysporum | EthFoc-36a | WEXO01 | 33971 | 45.0 |
| F. oxysporum | EthFoc-38 | WEXP01 | 3317 | 56.3 |
| F. oxysporum | EthFoc-4 | WEXQ01 | 2152 | 53.2 |
| F. oxysporum | EthFoc-40 | WEXR01 | 1508 | 51.6 |
| F. oxysporum | EthFoc-41 | WEXS01 | 1853 | 52.2 |
| F. oxysporum | EthFoc-42 | WEXT01 | 2498 | 53.6 |
| F. oxysporum | EthFoc-43 | WEXU01 | 3406 | 53.2 |
| F. oxysporum | EthFoc-44 | WEXV01 | 1573 | 52.3 |
| F. oxysporum | EthFoc-45 | WEXW01 | 31527 | 46.3 |
| F. oxysporum | EthFoc-48 | WEXX01 | 2674 | 53.1 |
| F. oxysporum | EthFoc-49 | WEXY01 | 9150 | 53.6 |
| F. oxysporum | EthFoc-52 | WEXZ01 | 1720 | 52.3 |
| F. oxysporum | EthFoc-55 | WEYA01 | 12910 | 58.3 |
| F. oxysporum | EthFoc-56 | WEYB01 | 2088 | 52.1 |
| F. oxysporum | EthFoc-57 | WEYC01 | 38587 | 40.0 |
| F. oxysporum | EthFoc-61 | WEYD01 | 2107 | 52.5 |
| F. oxysporum | EthFoc-62 | WEYE01 | 1316 | 43.0 |
| F. oxysporum | EthFoc-7 | WEYF01 | 2205 | 55.4 |
| F. oxysporum | EthFoc-75 | WEYG01 | 2057 | 53.6 |
| F. oxysporum | EthFoc-77 | WEYH01 | 2138 | 58.7 |
| F. oxysporum | EthFoc-78 | WEYI01 | 1889 | 51.6 |
| F. oxysporum | EthFoc-8 | WEYJ01 | 2097 | 55.6 |
| F. oxysporum | EthFoc-81 | WEYK01 | 3090 | 55.5 |
| F. oxysporum | EthFoc-83 | WEYL01 | 1937 | 53.0 |
| F. oxysporum | EthFoc-84 | WEYM01 | 2091 | 53.1 |
| F. oxysporum | EthFoc-86 | WEYN01 | 3099 | 59.4 |
| F. oxysporum | EthFoc-88 | WEYO01 | 8064 | 52.6 |
| F. oxysporum | EthFoc-90 | WEYP01 | 2235 | 54.3 |
| F. oxysporum | EthFoc-93 | WEYQ01 | 10282 | 52.4 |
| F. oxysporum | EthFoc-95 | WEYR01 | 2075 | 51.8 |
| F. oxysporum | EthFoc-98 | WEYS01 | 20735 | 49.9 |
| F. oxysporum | EthFoc-99 | WEYT01 | 3200 | 53.3 |
| F. oxysporum | EthFoc-DSP1 | WEYU01 | 22007 | 44.7 |
| F. oxysporum | EthFoc-DSP12 | WEYV01 | 8308 | 52.9 |
| F. oxysporum | EthFoc-DSP2 | WEYW01 | 23318 | 51.8 |
| F. oxysporum | EthFoc-DSP9 | WEYX01 | 27833 | 49.0 |
| F. oxysporum | EthFoc-35 | WEYY01 | 2273 | 54.6 |
| F. oxysporum | EthFoc-173 | WEYZ01 | 33788 | 40.1 |
| F. oxysporum | EthFoc-DSP6 | WEZA01 | 35863 | 38.2 |
| F. oxysporum | EthFoc-168 | WEZB01 | 34434 | 38.9 |
| F. oxysporum | EthFoc-152 | WEZC01 | 35107 | 38.1 |
| F. oxysporum | EthFoc-142 | WEZD01 | 36102 | 38.4 |
| F. oxysporum | EtdFoc-81 | WEZE01 | 6341 | 64.4 |
| F. oxysporum | EtdFoc-89 | WIKT01 | 30490 | 56.7 |
| F. oxysporum | EtdFoc-85 | WIKU01 | 5081 | 59.5 |
| F. oxysporum | EtdFoc-3 | WIKV01 | 1596 | 58.1 |
| F. oxysporum | EtdFoc-220 | WIKW01 | 5717 | 58.0 |
| F. oxysporum | EtdFoc-214 | WIKX01 | 860 | 58.1 |
| F. oxysporum | EtdFoc-206 | WIKY01 | 5774 | 60.6 |
| F. oxysporum | EtdFoc-20 | WIKZ01 | 25892 | 52.1 |
| F. oxysporum | EtdFoc-192 | WILA01 | 1870 | 58.3 |
| F. oxysporum | EtdFoc-191 | WILB01 | 1576 | 58.1 |
| F. oxysporum | EtdFoc-189 | WILC01 | 3207 | 58.6 |
| F. oxysporum | EtdFoc-182 | WILD01 | 28859 | 52.9 |
| F. oxysporum | EtdFoc-181 | WILE01 | 8664 | 55.3 |
| F. oxysporum | EtdFoc-176 | WILF01 | 24937 | 55.2 |
| F. oxysporum | EtdFoc-143 | WILG01 | 28349 | 52.1 |
| F. oxysporum | Fus259 | JAALGK01 | 821 | 49.3 |
| F. oxysporum | Fus250 | JAALGL01 | 1139 | 50.1 |
| F. oxysporum | Fus191 | JAALGM01 | 387 | 47.6 |
| F. oxysporum | Fus187 | JAALGN01 | 998 | 49.2 |
| F. oxysporum | Fus017 | JAALGS01 | 1518 | 51.4 |
| F. oxysporum | NRRL 39464 | JAAFOW01 | 5286 | 47.0 |
| F. oxysporum | isolate Fo74 | JAAMUY01 | 1912 | 52.5 |
| F. oxysporum | isolate Fo68 | JAAMUZ01 | 1371 | 51.5 |
| F. oxysporum | isolate Fo59 | JAAMVA01 | 680 | 48.8 |
| F. oxysporum | isolate Fo14 | JAAMVB01 | 2740 | 53.6 |
| F. oxysporum | isolate Fo9 | JAAMVC01 | 530 | 47.8 |
| F. oxysporum | isolate Fo7 | JAAMVD01 | 1809 | 53.4 |
| F. oxysporum | isolate Fo5 | JAAMVE01 | 2423 | 53.6 |
| F. oxysporum | isolate Fo47 | WXUE01 | 60 | 50.2 |
| F. oxysporum | isolate Fo75 | WXUF01 | 721 | 49.8 |
| F. oxysporum | isolate Fo69 | WXUG01 | 1419 | 53.3 |
| F. oxysporum | isolate Fo65 | WXUH01 | 2173 | 53.6 |
| F. oxysporum | isolate Fo63 | WXUI01 | 1510 | 52.3 |
| F. oxysporum | isolate Fo58 | WXUJ01 | 672 | 49.8 |
| F. oxysporum | isolate Fo57 | WXUK01 | 779 | 49.9 |
| F. oxysporum | isolate Fo54 | WXUL01 | 1094 | 50.8 |
| F. oxysporum | isolate Fo53 | WXUM01 | 1307 | 50.4 |
| F. oxysporum | isolate Fo52 | WXUN01 | 1523 | 51.2 |
| F. oxysporum | isolate Fo49 | WXUO01 | 1272 | 51.5 |
| F. oxysporum | isolate Fo48 | WXUP01 | 1272 | 51.5 |
| F. oxysporum | isolate Fo46 | WXUQ01 | 948 | 50.7 |
| F. oxysporum | isolate Fo45 | WXUR01 | 1443 | 50.5 |
| F. oxysporum | isolate Fo44 | WXUS01 | 856 | 49.3 |
| F. oxysporum | isolate Fo41 | WXUT01 | 1576 | 51.3 |
| F. oxysporum | isolate Fo39 | WXUU01 | 1175 | 50.1 |
| F. oxysporum | isolate Fo35 | WXUV01 | 616 | 48.1 |
| F. oxysporum | isolate Fo29 | WXUW01 | 1176 | 50.7 |
| F. oxysporum | isolate Fo28 | WXUX01 | 1834 | 52.1 |
| F. oxysporum | isolate Fo26 | WXUY01 | 1677 | 52.3 |
| F. oxysporum | isolate Fo25 | WXUZ01 | 1492 | 50.5 |
| F. oxysporum | isolate Fo24 | WXVA01 | 2026 | 52.6 |
| F. oxysporum | isolate Fo20 | WXVB01 | 1346 | 52.1 |
| F. oxysporum | isolate Fo18 | WXVC01 | 2144 | 54.9 |
| F. oxysporum | isolate Fo17 | WXVD01 | 1190 | 51.8 |
| F. oxysporum | isolate Fo16 | WXVE01 | 1476 | 51.7 |
| F. oxysporum | isolate Fo15 | WXVF01 | 1047 | 48.7 |
| F. oxysporum | isolate Fo13 | WXVG01 | 1253 | 50.8 |
| F. oxysporum | isolate Fo12 | WXVH01 | 1218 | 50.2 |
| F. oxysporum | isolate Fo11 | WXVI01 | 1086 | 49.9 |
| F. oxysporum | isolate Fo10 | WXVJ01 | 1683 | 52.7 |
| F. oxysporum | isolate Fo8 | WXVK01 | 1464 | 50.5 |
| F. oxysporum | isolate Fo6 | WXVL01 | 2072 | 52.6 |
| F. oxysporum | isolate Fo4 | WXVM01 | 1911 | 53.7 |
| F. oxysporum | isolate Fo3 | WXVN01 | 2910 | 56.7 |
| F. oxysporum | isolate Fo2 | WXVO01 | 616 | 47.5 |
| F. oxysporum | isolate Fo1 | WXVP01 | 723 | 49.1 |
| F. oxysporum f. sp. albedinis | Foa 133 | JAAVJG01 | 3325 | 56.2 |
| F. oxysporum f. sp. capsici | 14003 | JACYOB01 | 739 | 47.5 |
| F. oxysporum f. sp. cepae | FoC_Fus2 | MRCU01 | 34 | 53.4 |
| F. oxysporum f. sp. cepae | FoC_125 | MRCV01 | 2119 | 51.4 |
| F. oxysporum f. sp. cepae | FoC_A23 | MRCW01 | 1997 | 51.0 |
| F. oxysporum f. sp. ciceris | 38-1 | MEHF01 | 1482 | 54.8 |
| F. oxysporum f. sp. conglutinans | 1 | LPZQ01 | 13202 | 52.5 |
| F. oxysporum f. sp. conglutinans | 58385 | NRHZ01 | 5119 | 55.9 |
| F. oxysporum f. sp. conglutinans | FGL03-6 | NRIA02 | 1079 | 61.1 |
| F. oxysporum f. sp. conglutinans | Fo5176 | JACDXP01 | 19 | 68.0 |
| F. oxysporum f. sp. conglutinans | race 1 | JABTBS01 | 64 | 71.1 |
| F. oxysporum f. sp. conglutinans race 2 54008 | 54008 | AGNF01 | 3350 | 53.2 |
| F. oxysporum f. sp. cubense | C1HIR_9889 | MBFV01 | 1370 | 46.7 |
| F. oxysporum f. sp. cubense | 160527 | SRMI01 | 12 | 51.1 |
| F. oxysporum f. sp. cubense | isolate UK0001, TR4 | VMNF01 | 15 | 48.6 |
| F. oxysporum f. sp. cubense | TC1-1 | VLOF01 | 2635 | 48.6 |
| F. oxysporum f. sp. cubense | BC2-4 | VYQK01 | 4022 | 47.0 |
| F. oxysporum f. sp. cubense race 1 | race 1 | AMGP01 | 2185 | 46.9 |
| F. oxysporum f. sp. cucumerinum | Foc013 | MABJ01 | 1129 | 48.9 |
| F. oxysporum f. sp. cucumerinum | Foc015 | MABK01 | 1743 | 52.0 |
| F. oxysporum f. sp. cucumerinum | Foc021 | MABL01 | 3634 | 56.9 |
| F. oxysporum f. sp. cucumerinum | Foc018 | MABM01 | 3866 | 57.4 |
| F. oxysporum f. sp. cucumerinum | Foc030 | MABN01 | 3557 | 56.8 |
| F. oxysporum f. sp. cucumerinum | Foc035 | MABO01 | 1501 | 51.6 |
| F. oxysporum f. sp. cucumerinum | Foc037 | MABP01 | 1314 | 48.4 |
| F. oxysporum f. sp. cucumerinum | Foc011 | MABT01 | 1133 | 48.7 |
| F. oxysporum f. sp. cucumerinum | Foc001 | MAKZ01 | 1325 | 51.4 |
| F. oxysporum f. sp. gladioli | G76 | NJCK01 | 3146 | 56.1 |
| F. oxysporum f. sp. gladioli | G2 | NJCL01 | 2454 | 53.2 |
| F. oxysporum f. sp. gladioli | G14 | NJCM01 | 2859 | 56.3 |
| F. oxysporum f. sp. lagenariae | Lag1-1 | NJCG01 | 2079 | 54.6 |
| F. oxysporum f. sp. lagenariae | Lag3-1 | NJCH01 | 764 | 47.9 |
| F. oxysporum f. sp. lagenariae | 03-05118 | NJCI01 | 2650 | 56.1 |
| F. oxysporum f. sp. lagenariae | 01-03008 | NJCJ01 | 1728 | 53.9 |
| F. oxysporum f. sp. lilii | Fol39 | NJCF01 | 1677 | 53.4 |
| F. oxysporum f. sp. lini | 39 | WHMS01 | 35 | 59.2 |
| F. oxysporum f. sp. lini | 39 | JABJUA01 | 26 | 69.5 |
| F. oxysporum f. sp. lini | F282 | JABJUB01 | 185 | 48.0 |
| F. oxysporum f. sp. lini | F287 | JABJUC01 | 180 | 47.6 |
| F. oxysporum f. sp. lini | F324 | JABJUD01 | 35 | 47.5 |
| F. oxysporum f. sp. lini | F329 | JABJUE01 | 197 | 46.9 |
| F. oxysporum f. sp. luffae | Fol-167 | NJCD01 | 590 | 48.3 |
| F. oxysporum f. sp. luffae | Fol-114 | NJCE01 | 589 | 48.2 |
| F. oxysporum f. sp. lycopersici | Fol004 | MALH01 | 3433 | 51.9 |
| F. oxysporum f. sp. lycopersici | Fol007 | MALI01 | 1999 | 51.3 |
| F. oxysporum f. sp. lycopersici | Fol014 | MALJ01 | 2589 | 50.1 |
| F. oxysporum f. sp. lycopersici | Fol026 | MALK01 | 3302 | 51.8 |
| F. oxysporum f. sp. lycopersici | Fol018 | MALL01 | 2140 | 49.0 |
| F. oxysporum f. sp. lycopersici | Fol016 | MALM01 | 2336 | 49.6 |
| F. oxysporum f. sp. lycopersici | Fol029 | MALN01 | 2270 | 50.0 |
| F. oxysporum f. sp. lycopersici | Fol038 | MALO01 | 3188 | 51.3 |
| F. oxysporum f. sp. lycopersici | Fol069 | MALP01 | 1888 | 49.1 |
| F. oxysporum f. sp. lycopersici | Fol072 | MALQ01 | 1859 | 50.1 |
| F. oxysporum f. sp. lycopersici | Fol073 | MALR01 | 3317 | 51.8 |
| F. oxysporum f. sp. lycopersici | Fol074 | MALS01 | 3410 | 51.5 |
| F. oxysporum f. sp. lycopersici | Fol075 | MALT01 | 2009 | 50.6 |
| F. oxysporum f. sp. lycopersici | Fol002 | MAMG01 | 3451 | 51.7 |
| F. oxysporum f. sp. lycopersici | isolate D11, race 3 | RBXW01 | 39 | 57.3 |
| F. oxysporum f. sp. lycopersici 4287 | 4287 | AAXH01 | 1371 | 60.0 |
| F. oxysporum f. sp. lycopersici 4287 | 4287 | MALV01 | 4285 | 51.1 |
| F. oxysporum f. sp. lycopersici 4287 | 4287 | MALW01 | 1955 | 51.0 |
| F. oxysporum f. sp. lycopersici 4287 | 4287 | QESU01 | 499 | 53.9 |
| F. oxysporum f. sp. lycopersici MN25 | MN25 | AGBH01 | 801 | 48.5 |
| F. oxysporum f. sp. matthiolae | PHW726 | WJXY01 | 583 | 57.3 |
| F. oxysporum f. sp. medicaginis | isolate Fom-5190a | LSNI01 | 5373 | 49.2 |
| F. oxysporum f. sp. melongenae | 14004 | MPIL01 | 1631 | 54.0 |
| F. oxysporum f. sp. melongenae | J-71 | NJCC01 | 1726 | 52.3 |
| F. oxysporum f. sp. melonis | Fom004 | MALX01 | 1300 | 57.7 |
| F. oxysporum f. sp. melonis | Fom005 | MALY01 | 2472 | 54.5 |
| F. oxysporum f. sp. melonis | Fom006 | MALZ01 | 2225 | 52.8 |
| F. oxysporum f. sp. melonis | Fom009 | MAMA01 | 1727 | 57.7 |
| F. oxysporum f. sp. melonis | Fom010 | MAMB01 | 3384 | 53.7 |
| F. oxysporum f. sp. melonis | Fom011 | MAMC01 | 2590 | 52.9 |
| F. oxysporum f. sp. melonis | Fom012 | MAMD01 | 2283 | 52.6 |
| F. oxysporum f. sp. melonis | Fom013 | MAME01 | 2256 | 51.9 |
| F. oxysporum f. sp. melonis | Fom016 | MAMF01 | 2257 | 52.9 |
| F. oxysporum f. sp. melonis 26406 | 26406 | AGNE01 | 1825 | 53.8 |
| F. oxysporum f. sp. melonis 26406 | 26406 | NJCY01 | 96 | 60.7 |
| F. oxysporum f. sp. momordicae | 90NF2-1 | NJCA01 | 1089 | 51.0 |
| F. oxysporum f. sp. momordicae | NRRL26413 | NJCB01 | 1317 | 51.5 |
| F. oxysporum f. sp. narcissi | Na5 | NJCV01 | 3497 | 55.5 |
| F. oxysporum f. sp. narcissi | N139 | MQTW01 | 4349 | 57.5 |
| F. oxysporum f. sp. nicotianae | Ft-Rob | NJBX01 | 1196 | 49.8 |
| F. oxysporum f. sp. nicotianae | 10913 | NJBY01 | 638 | 50.0 |
| F. oxysporum f. sp. nicotianae | FON-1 | NJBZ01 | 682 | 50.2 |
| F. oxysporum f. sp. nicotianae | Ft-1512 | NJCU01 | 989 | 51.0 |
| F. oxysporum f. sp. niveum | Fon005 | MAKY01 | 3511 | 54.7 |
| F. oxysporum f. sp. niveum | Fon002 | MALA01 | 2191 | 52.0 |
| F. oxysporum f. sp. niveum | Fon010 | MALB01 | 3377 | 55.5 |
| F. oxysporum f. sp. niveum | Fon013 | MALC01 | 3008 | 54.2 |
| F. oxysporum f. sp. niveum | Fon015 | MALD01 | 2383 | 52.1 |
| F. oxysporum f. sp. niveum | Fon020 | MALE01 | 3604 | 55.1 |
| F. oxysporum f. sp. niveum | Fon037 | MALF01 | 1737 | 51.3 |
| F. oxysporum f. sp. niveum | Fon021 | MALG01 | 3081 | 54.8 |
| F. oxysporum f. sp. niveum | Fon019 | MAMH01 | 1758 | 49.7 |
| F. oxysporum f. sp. niveum | R3 | JACRUX01 | 7170 | 55.2 |
| F. oxysporum f. sp. niveum | R1 | JACRUY01 | 8668 | 61.2 |
| F. oxysporum f. sp. niveum | R2 | JACRUZ01 | 6119 | 54.1 |
| F. oxysporum f. sp. pisi HDV247 | HDV247 | AGBI01 | 1744 | 54.5 |
| F. oxysporum f. sp. radicis-cucumerinum | Forc024 | MABR01 | 824 | 49.3 |
| F. oxysporum f. sp. radicis-cucumerinum | Forc031 | MABS01 | 879 | 49.3 |
| F. oxysporum f. sp. radicis-cucumerinum | Forc016 | MABQ02 | 33 | 52.9 |
| F. oxysporum f. sp. radicis-lycopersici 26381 | 26381 | AGNB01 | 725 | 49.3 |
| F. oxysporum f. sp. raphani 54005 | 54005 | AGNG01 | 2323 | 52.9 |
| F. oxysporum f. sp. spinaciae | Fus254 | JAALGI01 | 279 | 56.3 |
| F. oxysporum f. sp. spinaciae | Fus322 | JAALGJ01 | 4003 | 56.1 |
| F. oxysporum f. sp. spinaciae | Fus173 | JAALGO01 | 3021 | 56.2 |
| F. oxysporum f. sp. spinaciae | Fus167 | JAALGP01 | 3748 | 56.7 |
| F. oxysporum f. sp. spinaciae | Fus059 | JAALGQ01 | 4007 | 56.1 |
| F. oxysporum f. sp. spinaciae | Fus057 | JAALGR01 | 4042 | 56.2 |
| F. oxysporum f. sp. spinaciae | Fus001 | JAALGT01 | 3098 | 56.2 |
| F. oxysporum f. sp. tulipae | Tu67 | NJBS01 | 2263 | 54.3 |
| F. oxysporum f. sp. vasinfectum | isolate TF1 | VINL01 | 17 | 50.0 |
| F. oxysporum f. sp. vasinfectum | isolate LA3B | VINM01 | 17 | 50.4 |
| F. oxysporum f. sp. vasinfectum | isolate LA1E | VINN01 | 26 | 53.5 |
| F. oxysporum f. sp. vasinfectum | isolate 14-004 | VINO01 | 18 | 51.7 |
| F. oxysporum f. sp. vasinfectum | isolate 89-1A | VINP01 | 94 | 63.3 |
| F. oxysporum f. sp. vasinfectum | NRRL 25420 | JAANYJ01 | 581 | 52.0 |
| F. oxysporum f. sp. vasinfectum | NRRL 25434 | JAANYK01 | 861 | 67.8 |
| F. oxysporum f. sp. vasinfectum | NRRL 25432 | JAANYL01 | 634 | 68.7 |
| F. oxysporum f. sp. vasinfectum | NRRL 31665 | JAANYM01 | 433 | 54.9 |
| F. oxysporum f. sp. vasinfectum 25433 | 25433 | AGNC01 | 1947 | 52.5 |
| F. oxysporum Fo47 | Fo47 | AFMM01 | 419 | 49.4 |
| F. oxysporum Fo5176 | Fo5176 | AFQF01 | 7858 | 54.8 |
| F. oxysporum NRRL 32931 | NRRL 32931 | AFML01 | 545 | 47.2 |
| F. penzigii | NRRL 20711 | JABFFC01 | 1247 | 36.3 |
| F. phaseoli | NRRL 31156 | MAEB01 | 16072 | 50.5 |
| F. phaseoli | NRRL 22396 | JABEEL01 | 1483 | 46.1 |
| F. phyllophilum | NRRL 13617 | JAAOAQ01 | 1628 | 43.4 |
| F. pininemorale | CMW 25243 | NFZR01 | 153 | 47.8 |
| F. poae | 2516 | LYXU01 | 181 | 46.5 |
| F. poae | NRRL 26941 | JABFFD01 | 2426 | 37.8 |
| F. praegraminearum | NRRL 39664 | LXHY01 | 481 | 35.3 |
| F. proliferatum | CF-295141 | MBPS01 | 237 | 44.2 |
| F. proliferatum | NRRL62905 | FCQG01 | 155 | 43.2 |
| F. proliferatum | Fol3 | NJCT01 | 10475 | 64.2 |
| F. proliferatum | COH1152 | QBDQ01 | 2117 | 47.7 |
| F. proliferatum | ITEM 2341 | PKMI01 | 104 | 45.5 |
| F. proliferatum | Fp_A8 | MRDB01 | 581 | 45.7 |
| F. proliferatum | MOD1-FUNGI19 | RBBY01 | 449 | 43.1 |
| F. proliferatum | MOD1-FUNGI15 | RBCB01 | 578 | 43.3 |
| F. proliferatum | MOD1-FUNGI14 | RBCC01 | 563 | 43.1 |
| F. proliferatum | MOD1-FUNGI13 | RBCD01 | 1006 | 43.1 |
| F. proliferatum | MOD1-FUNGI12 | RBCE01 | 562 | 43.1 |
| F. proliferatum | MOD1-FUNGI8 | RBJG01 | 1159 | 43.2 |
| F. proliferatum | NRRL 66682 | JABSTG01 | 1501 | 45.7 |
| F. proliferatum | NRRL 43689 | JABSTK01 | 1442 | 44.4 |
| F. proliferatum ET1 | ET1 | FJOF01 | 32 | 45.2 |
| F. protoensiforme | NRRL 22178 | JAANQP01 | 1682 | 45.5 |
| F. pseudoanthophilum | NRRL 25211 | JAAOAR01 | 1816 | 42.8 |
| F. pseudocircinatum | NRRL 36939 | JAAOAS01 | 1040 | 43.3 |
| F. pseudograminearum | CS3270 | JTGB02 | 89 | 37.1 |
| F. pseudograminearum | RBG5266 | JTGC01 | 735 | 36.3 |
| F. pseudograminearum CS3096 | CS3096 | AFNW01 | 685 | 36.9 |
| F. pseudograminearum CS3220 | CS3220 | CBMC01 | 1479 | 37.3 |
| F. pseudograminearum CS3427 | CS3427 | CBMD01 | 1715 | 37.2 |
| F. pseudograminearum CS3487 | CS3487 | CBME01 | 2589 | 37.1 |
| F. pseudograminearum CS5834 | CS5834 | CBMF01 | 1396 | 37.6 |
| F. pseudonygamai | NRRL 13592 | JAAQRH01 | 2619 | 42.2 |
| F. ramigenum | NRRL 25208 | JAAOAT01 | 1577 | 46.5 |
| F. sacchari | NRRL 66326 | JABSTH01 | 515 | 42.8 |
| F. sambucinum | F-4 | LSRD01 | 961 | 37.8 |
| F. sarcochroum | NRRL 20472 | JABEXW01 | 1849 | 46.4 |
| F. scirpi | NRRL 66328 | QHHJ01 | 587 | 39.6 |
| F. secorum | NRRL 62593 | JABEEM01 | 6851 | 51.2 |
| F. setosum | NRRL 36526 | JABFFE01 | 3402 | 46.2 |
| F. solani | JS-169 | NGZQ01 | 17 | 45.8 |
| F. solani | isolate S2_018_000R2 | SCGO01 | 2622 | 44.9 |
| F. solani | isolate S2_009_000R2b | SCGU01 | 4372 | 31.1 |
| F. solani | LQ1 | WXYL01 | 1305 | 51.9 |
| F. solani | IISc-1 | JABJVU01 | 117 | 48.8 |
| F. sp. AF-3 | NRRL62606 | NKCL01 | 1719 | 47.4 |
| F. sp. AF-4 | NRRL62579 | NKCK01 | 1171 | 48.7 |
| F. sp. AF-6 | NRRL62590 | NKCJ01 | 1182 | 43.1 |
| F. sp. AF-8 | NRRL62584 | NKCI01 | 1006 | 47.5 |
| F. sp. BWC | BWC | SWCQ01 | 435 | 42.1 |
| F. sp. DS 682 | DS 682 | JACYFE01 | 11597 | 56.1 |
| F. sp. JS1030 | JS1030 | JWIW01 | 107 | 53.7 |
| F. sp. JS626 | JS626 | JWIV01 | 63 | 42.9 |
| F. sp. KOD 1611 | KOD 1611 | JABKKQ01 | 5049 | 38.9 |
| F. sp. Na10 | Na10 | NJCS01 | 971 | 46.6 |
| F. sp. NRRL 22101 | NRRL 22101 | JABELF01 | 4875 | 65.6 |
| F. sp. NRRL 25184 | NRRL 25184 | JABSSZ01 | 2609 | 48.4 |
| F. sp. NRRL 25303 | NRRL 25303 | JAAOAY01 | 941 | 43.7 |
| F. sp. NRRL 29148 | NRRL 29148 | JABSTL01 | 2621 | 40.7 |
| F. sp. NRRL 47473 | NRRL 47473 | JABSTM01 | 1043 | 43.4 |
| F. sp. NRRL 52700 | NRRL 52700 | JAAQRM01 | 528 | 43.0 |
| F. sp. NRRL 53293 | NRRL 53293 | JABSTN01 | 859 | 44.0 |
| F. sp. NRRL 53497 | NRRL 53497 | JABCJZ01 | 882 | 44.2 |
| F. sp. NRRL 6227 | NRRL 6227 | JABFEC01 | 599 | 37.3 |
| F. sp. NRRL 62610 | NRRL 62610 | JABCKE02 | 12062 | 46.1 |
| F. sp. NRRL 62941 | NRRL 62941 | JAALXN01 | 4472 | 48.1 |
| F. sp. NRRL 62944 | NRRL 62944 | JABCKC01 | 8878 | 48.0 |
| F. sp. NRRL 62957 | NRRL 62957 | JAALXM01 | 18524 | 43.8 |
| F. sp. NRRL 66088 | NRRL 66088 | JABCKD01 | 4445 | 48.7 |
| F. sp. NRRL 66182 | NRRL 66182 | JABFAK01 | 16970 | 48.0 |
| F. sp. QHM | QHM | SWCP01 | 435 | 42.1 |
| F. sporotrichioides | NRRL 3299 | PXOF01 | 446 | 37.4 |
| F. sterilihyphosum | NRRL 25623 | JAAOAU01 | 2178 | 46.9 |
| F. subglutinans | RC 528 | JAAIFQ01 | 1355 | 44.1 |
| F. subglutinans | RC 298 | JAAIFR01 | 3933 | 51.7 |
| F. subglutinans | NRRL 66333 | JAAOAV01 | 905 | 44.2 |
| F. sublunatum | NRRL 13384 | JABFFF01 | 986 | 35.7 |
| F. subtropicale | NRRL 66764 | QRAA01 | 1080 | 35.4 |
| F. succisae | NRRL 13298 | JAAOAW01 | 538 | 45.4 |
| F. tanahbumbuense | NRRL 66471 | JAALXH01 | 1049 | 37.8 |
| F. temperatum | CMWF389 | LJGR01 | 43 | 45.5 |
| F. temperatum | RC 2914 | JAAIFN01 | 667 | 43.4 |
| F. temperatum | KFI 615 | JACOPY01 | 20 | 45.2 |
| F. temperatum | KFI 660 | JACOPZ01 | 18 | 45.0 |
| F. thapsinum | NRRL 22049 | JAAOAX01 | 384 | 40.9 |
| F. tjaetaba | NRRL 66243 | JAAQRI01 | 867 | 43.1 |
| F. torulosum | NRRL 22747 | JABFMN01 | 1339 | 41.3 |
| F. transvaalense | NRRL 31008 | JABFFG01 | 1088 | 37.9 |
| F. tricinctum | T6 | PTXX01 | 617 | 42.7 |
| F. tricinctum | INRA104 | QFZF01 | 23 | 42.8 |
| F. tricinctum |  | OVTS02 | 23 | 42.8 |
| F. tricinctum | NRRL 25481 | JAALXJ01 | 1032 | 41.0 |
| F. tuaranense | NRRL 46518 | JABEEN01 | 3977 | 48.9 |
| F. tucumaniae | NRRL 31096 | MAED01 | 15093 | 48.4 |
| F. tucumaniae | NRRL 31781 | MAEE01 | 22979 | 49.7 |
| F. tucumaniae | NRRL 34546 | MAEF01 | 54001 | 49.9 |
| F. tupiense | NRRL 53984 | JABEEO01 | 1664 | 45.5 |
| F. udum | F-02845 | NIFK01 | 712 | 56.4 |
| F. udum | NRRL 25194 | JAAQPG01 | 2353 | 44.6 |
| F. vanettenii 77-13-4 | isolate 77-13-4 | ACJF01 | 233 | 51.2 |
| F. venenatum | NRRL 66329 | JABFFH01 | 616 | 38.0 |
| F. verrucosum | NRRL 22566 | JABFFJ01 | 767 | 34.2 |
| F. verticillioides | BRIP14953 | QFXM01 | 1060 | 42.5 |
| F. verticillioides | BRIP53263 | QJUS01 | 931 | 42.3 |
| F. verticillioides | BRIP53590 | QKXB01 | 1009 | 42.2 |
| F. verticillioides | NRRL 20984 | JABSTI01 | 857 | 41.9 |
| F. verticillioides 7600 | 7600 | AAIM02 | 213 | 41.8 |
| F. virguliforme | Clinton-1B | MADX01 | 25997 | 50.9 |
| F. virguliforme | LL0009 | MADY01 | 25286 | 50.2 |
| F. virguliforme | NRRL 34551 | MADZ01 | 20982 | 49.4 |
| F. virguliforme | NRRL 31041 | JABEEP01 | 3852 | 45.4 |
| F. virguliforme Mont-1 | Mont-1 | AEYB01 | 3098 | 50.4 |
| F. xylarioides | K1 | RXHO01 | 1132 | 55.1 |
| F. xylarioides | KSU18978 | SRZU01 | 424 | 55.2 |
| F. xylarioides | NRRL 25486 | JABFFK01 | 2987 | 44.0 |
| F. xyrophilum | NRRL 66890 | VYWY01 | 5402 | 38.1 |
| F. xyrophilum | NRRL 62721 | VYWZ01 | 5229 | 39.6 |
| F. xyrophilum | NRRL 62710 | VYXA01 | 5060 | 38.9 |
| F. zanthoxyli | NRRL 66285 | JABFFL01 | 2368 | 40.9 |
| F. zealandicum | NRRL 22465 | JABEYC01 | 1836 | 33.5 |

^a^Downloaded from GenBank on 30 September 2020.
