# Supplementary material for: Genomic differences between the new Fusarium oxysporum f. sp. apii (Foa) race 4 on celery, the less virulent Foa races 2 and 3, and the avirulent on celery f. sp. coriandrii
Source: BMC Genomics. 2020 Oct 20;21:730. doi: 10.1186/s12864-020-07141-5 (PMC7576743; doi:10.1186/s12864-020-07141-5)
Supplement: Supplementary file 20 — Additional file 20 Diagnostic PCR primers for Foa race 2 haplogroup, Foa race 4, and Foci [file 12864_2020_7141_MOESM20_ESM.docx]

| **Additional file 20.** Diagnostic PCR primers for *Foa* race 2 haplogroup, *Foa* race 4, and *Foci*^a^*.*   \| Target strain \| Primer name \| Forward primer (5’3’) sequence \| Reverse primer (5’3’) sequence \| Amplicon size, bp \| Annealing temp, C \| *F. oxysporum* strains that produce an undesired positive or an undersired negative based on either a NCBI GenBank wgs BLAST (D) or empirical testing (E)^b^ \| Location of amplicon:bp start-end in either core genome, host-specific (HS) accessory or lineage-specific (LS) accessory genome \| \| \| \| --- \| --- \| --- \| --- \| --- \| --- \| --- \| --- \| --- \| --- \| \| *Foa*  race 2 \| FOA R2-76k^c,d^ \| TGTTGAGTTGTCGGAGTTCTGC \| TCCTTGTGTTTCTCGGTTCCTC \| 226 \| 63 \| None detected \| Contig_9:76612-76837 (Accessory) \| \| \| \| *Foa*  race 4 \| FOAR4-447^d^ \| AACCCAAGGTCTCGACGATCTG \| ACCTTCCGTGCAGTCCTCATTC \| 196 \| 63 \| Undesired Positives: *Foci*10T (E); f. sp. *vasinfectum* NRRL 25433 (D,E) and race 4, 89-1A (D), f. sp. *raphanini* NRRL 54005 (D,E), f. sp. *gladioli* G76 (D); *Fo* strains VPRI31638, VPRI16235 VPRI10405 and Fo9 (D), and *F. secorum* NRRL 62593 (D) \| In *Foa* race 4, in Superscaffold_5: 4476883-4477078 (Core, Chromosome 11)^f^ \| \| \| \| \| *Foa*  race 4 \| N3875-2^e^ \| AGGACTTGAATCACGGCTCG \| CCTGCCACTCGCTTTTTGAG \| 189 \| 60 \| Undesired Positives: *Foci*GL306 and *Foci*3-2 (D,E), *Foci*8.1A, *Foci*10E, *Foci*11, *Foci*12A and *Foci*10T (E); *F. oxysporum* non-pathogens from symptomatic celery (273-1B and 273-2B) (E); highly similar to f. sp. *pisi* RBG6397 and RBG6454, and *F. oxysporum* VPRI42180 (D) \| In *Foa* race 4, two copies in Superscaffold_2:482422-482253 and 464741-464929 (LS Accessory); In single copies in *Foci*3-2_Contig 111586365-1586553 (LS Accessory) and in *Foci*GL306_ Contig 13:1059996-1060184 (Accessory) \| \| \| \| \| *Foci* \| FOCI2-21^d,f^ \| GTAGTATCGTGGGATTGGCGTTTG \| GGCCTCTTCTGAATTGTCGCATAC \| 374 \| 63 \| Undesired Positives: *Fo* strains VPRI11409 and f. sp. *albedinis* 133, similar to *Fo* strains Fo65 and Fo45, *F. mundagurra* NRRL 66235, *F. autroafricanum* NRRL 53441, and *Fo* strains from soil: EtdFoc-209, EtdFoc-208, RBG5714, RBG6313, Fo3, Fo4, and Fo5 (D)  Undesired Negatives: *Foci*10T (E) \| \| *Foc*i3-2_Contig_21:490260-490633 (HS Accessory);  *Foci*GL306_Contig_20:790595-790968 (HS Accessory) \| \| *Foci* \| FOCI-g_c31 \| TGGTTCATCTATCCCTCAAGGAGTATC \| AGCCTTTATTCTCGTCCATCATAAGTTC \| 402 \| 60 \| Undesired Positives: *Fo* strains from soil: Fo3 and Fo5 (D).  Undesired Negatives: *Foci*10T (E) \| \| *Foci*3-2_Contig 12:962890-962489 (HS Accessory) *Foci*GL306_Contig 31: 120557-120156 (HS Accessory) \|   ^a^*Foa, F. oxysporum* f. sp. *apii*; *Foci, F. oxysporum* f. sp. *coriandrii*.  ^b^For *in silico* testing, the amplicons were BLASTed with the GenBank database on 30 September 2020 with 1) the nr/nt database, and had no significant results, and 2) on the *Fusarium* wgs databases with 437 *F. oxysporum* isolates and 329 other *Fusarium* *spp*; significant hits are noted. A list of the 772 *Fusarium spp*. in the wgs database is shown in Additional file 19. For empirical testing, we included those in our *F. oxysporum* collection (including non-pathogens and pathogens from celery [3] and isolates from coriander (Additional file 18).  ^c^Primer pair FOA_R2-76 is suitable for cultures. For analyses with plant debris, we include a fluorescently labeled probe, 5’56-FAM/TCTGCTTCT/ZEN/AGGCTGTGCTCGAAAGCT/3IAbkFQ/-3’ (IDT, San Jose, CA); the probe eliminates cross reaction with celery tissue.  ^d^These primer pairs can be reacted in a multiplex with SYBR Green.  ^e^The primer sequences and the undesired positives with two non-pathogenic *F. oxysporum* strains from California celery were previously published in Epstein et al.[3].  ^f^As shown in Fig. 3D, the homolog of *F. oxysporum* f. sp. *lycopersici* 4287 core chromosome 11 in *Foa* race 4 (SS5) has regions that are not conserved. |
| --- | --- | --- | --- | --- | --- | --- | --- | --- | --- | --- | --- | --- | --- | --- | --- | --- | --- | --- | --- | --- | --- | --- | --- | --- | --- | --- | --- | --- | --- | --- | --- | --- | --- | --- | --- | --- | --- | --- | --- | --- | --- | --- | --- | --- | --- | --- | --- | --- | --- | --- | --- | --- | --- | --- | --- | --- | --- | --- | --- | --- |
