# Supplementary material for: Genomic differences between the new Fusarium oxysporum f. sp. apii (Foa) race 4 on celery, the less virulent Foa races 2 and 3, and the avirulent on celery f. sp. coriandrii
Source: BMC Genomics. 2020 Oct 20;21:730. doi: 10.1186/s12864-020-07141-5 (PMC7576743; doi:10.1186/s12864-020-07141-5)
Supplement: Supplementary file 21 — Additional file 21 Percentage of the genome with transposons and repeats in Foa, Foci, and the Fol reference [file 12864_2020_7141_MOESM21_ESM.docx]

**Additional file 21.**  Percentage of the genome with transposons and repeats in *Foa, Foci,* and the *Fol* reference^a^

|  | Type of transposable element | | | | | | Total interspersed repeats |
| --- | --- | --- | --- | --- | --- | --- | --- |
|  | DNA elements | LTR elements | LINES | | SINES | Unclassified |  |
|  | Percentage of genome | | | | | | |
| *Foa* race 4 | 6.24 | 4.94 | | 2.13 | 0.00 | 6.25 | 19.56 |
| *Foa* race 3 | 5.68 | 4.22 | | 2.11 | 0.01 | 8.06 | 20.07 |
| *Foci*3-2 | 6.21 | 3.50 | | 1.86 | 0.00 | 6.64 | 18.22 |
| *Foci*GL306 | 7.26 | 3.66 | | 2.00 | 0.01 | 5.67 | 18.60 |
| *Foa* race 2 | 7.59 | 3.64 | | 1.79 | 0.01 | 5.67 | 18.70 |
| *Fol*4287 | 7.04 | 1.98 | | 2.00 | 0.01 | 5.76 | 16.79 |

^a^All elements were identified with RepeatMasker 4.0.
